# Genome-wide analyses of miniature inverted-repeat transposable elements reveals new insights into the evolution of the *Triticum-Aegilops* group

**DOI:** 10.1371/journal.pone.0204972

**Published:** 2018-10-24

**Authors:** Danielle Keidar-Friedman, Inbar Bariah, Khalil Kashkush

**Affiliations:** Department of Life Sciences, Ben-Gurion University, Beer-Sheva, Israel; Universiteit Gent, BELGIUM

## Abstract

The sequence drafts of wild emmer and bread wheat facilitated high resolution, genome-wide analysis of transposable elements (TEs), which account for up to 90% of the wheat genome. Despite extensive studies, the role of TEs in reshaping nascent polyploid genomes remains to be fully understood. In this study, we retrieved miniature inverted-repeat transposable elements (MITEs) from the recently published genome drafts of *Triticum aestivum*, *Triticum turgidum* ssp. *dicoccoides*, *Aegilops tauschii* and the available genome draft of *Triticum urartu*. Overall, 239,126 MITE insertions were retrieved, including 3,874 insertions of a newly identified, wheat-unique MITE family that we named “*Inbar*”. The *Stowaway* superfamily accounts for ~80% of the retrieved MITE insertions, while *Thalos* is the most abundant family. MITE insertions are distributed in the seven homologous chromosomes of the wild emmer and bread wheat genomes. The remarkably high level of insertions in the B sub-genome (~59% of total retrieved MITE insertions in the wild emmer genome draft, and ~41% in the bread wheat genome draft), emphasize its highly repetitive nature. Nearly 52% of all MITE insertions were found within or close (less than 100bp) to coding genes, and ~400 MITE sequences were found in the bread wheat transcriptome, indicating that MITEs might have a strong impact on wheat genome expression. In addition, ~40% of MITE insertions were found within TE sequences, and remarkably, ~90% of *Inbar* insertions were located in retrotransposon sequences. Our data thus shed new light on the role of MITEs in the diversification of allopolyploid wheat species.

## Introduction

The origin of wheat (*Triticum-Aegilops* group) dates back some 4 million years ago, with the divergence of three ancestral species from a common progenitor, namely *Triticum urartu* (donor of the A genome), an unknown *Aegilops* species from the *sitopsis* section (donor of the B genome) and *Aegilops tauschii* (donor of the D genome) [1]. The first allopolyploidization event included hybridization of *T*. *urartu* with an *Aegilops* species, leading to the formation of the tetraploid *Triticum turgidum* (wild emmer, genome AB) around 500,000 years ago [2]. The second event included hybridization between *T*. *turgidum* and *Ae*. *tauschii*, resulting in the formation of the hexaploid *T*. *aestivum* (bread wheat, genome ABD) around 10,000 years ago [1, 3]. Newly formed allopolyploid species reveal massive genome reorganizations and epigenetic modifications that affect the regulation of gene expression, causing the activation of some transposon families and the silencing of others [2, 4, 5].

Transposable elements (TEs) are DNA fragments that can change their location and proliferate within the host genome and are found in all organisms investigated to date. Over 80% of the wheat genome comprises TEs [6–9]. Plant TEs can be divided into two main classes. Class I TEs, termed RNA elements or retrotransposons, move via a “copy and paste” mechanism involving an RNA intermediate. Class II TEs, termed DNA transposons, translocate via a “cut and paste” mechanism without the involvement of any intermediate molecules [4]. These TE classes can be sub-divided into superfamilies and families, with a given genome possibly consisting of hundreds or thousands of different families. Superfamilies within the same order share a replication strategy but differ in terms of their DNA sequence and target site duplication (TSD) size. Families, on the other hand, are defined by DNA sequence conservation [4, 10]. Finally, active TEs can affect genome structure and function [11–13].

Recent studies showed that the non-autonomous miniature elements termed miniature inverted-repeat transposable elements (MITEs) are one of the most active TEs in eukaryotes [14–17]. MITEs belong to the order TIR (Tandem Inverted Repeat) of the DNA transposons. MITEs are short DNA elements, comprising tens to several hundred base pairs, and are found only in eukaryotic genomes [4, 18]. MITEs are found in high copy numbers in some plant species, such as rice [19] and maize [18, 20], and were shown to be in strong association with genes [10, 21–24]. MITEs were also shown to be active in plants [14, 15, 19, 25], where they affect the expression of genes by insertion into introns, promotors or other gene regulatory sequences [26–29].

In a previous study, ~18,000 insertions of 18 *Stowaway-like* MITE families in wheat were analyzed [30] using the shotgun sequence draft of a 454-pyrosequence of *T*. *aestivum* [31]. The availability of genome drafts for four *Triticum* and *Aegilops* species, specifically the updated sequence draft of *T*. *aestivum* [7] and the recently published genome drafts of *T*. *turgidum* ssp. *dicoccoides* [8] and *Aegilops tauschii* [32], allowed for genome-wide high-resolution analysis of TEs. In this study, we performed genome-wide analysis of all known MITE families in wheat in four genome drafts, namely *T*. *aestivum* (genome ABD), *T*. *turgidum dicoccoides* (genome AB), *Ae*. *tauschii* (genome D) and *T*. *urartu* (genome A). The known MITE superfamilies in wheat genomes are *Stowaway*, characterized by very short length (70–350 bp) elements and a TSD corresponding to the TA dinucleotide [33], *Tourist*, corresponding to a size of 100–400 bp and a TSD of TWA (W = a/t), and *Mutator*, characterized by sequences sized 100–700 bp and a long and varying TSD (7–10 bp) [18]. We retrieved 235,252 known MITE insertions from these four wheat genome drafts, mostly belonging to the *Stowaway* superfamily. In addition, we discovered a new wheat-unique MITE family, termed *Inbar*, and retrieved 3,874 such insertions from the 4 genome drafts. The impact of MITEs on genome structure and function is discussed.

## Materials and methods

### Wheat genomic and transcriptomic sources

In this study, we used genome drafts of four *Triticum* and *Aegilops* species. *T*. *urartu*, the donor of A genome, was sequenced by Illumina using a whole-genome paired-end shotgun approach. The sequenced reads were assembled using SOAP denovo and the sequence depth of most (96%) reads was over 20x, with a peak at 85x (http://plants.ensembl.org/Triticum_urartu/Info/Index) [34]. *Ae*. *tauschii* ssp *strangulata* accession AL8/78, a close relative of the D genome donor, was sequenced using BAC and whole-genome shotgun approaches. These sequences were assembled to a create genome draft of 4.3 Gbp covering 95.2% of the genome sequence [32]. *T*. *turgidum* ssp *dicoccoides* is wild emmer wheat (WEWseq: http://wewseq.wix.com/consortium). The full genome draft of emmer wheat (*Triticum turgidum* ssp. *dicoccoides*) containing sorted chromosomes was sequenced using paired-end and mate-pair shotgun sequencing to a depth of 190x, and was recently published [8]. Sequenced reads were assembled using the DeNovoMAGIC tool (created by NRGene) to cover ~95% of the emmer wheat genome. The genome of *T*. *aestivum*, the hexaploid bread wheat, was sequenced in June, 2016 and can be found at *EnsemblPlants* [7] (pre.plants.ensembl.org/Triticum_aestivum/Info/Index). This updated *T*. *aestivum* assembly was generated by The Genome Analysis Center in Norwich (TGACv1). The 2016 update of *T*. *aestivum* assembly, TGACv1, covers 13.4 Gbp of the genome with an N50 of 88.8 kbp. Scaffolding was carried on using SOAPdenovo and CSS reads [9].

#### RNA-seq database

We used the updated publicly available RNA-seq database of *T*. *aestivum* found at *Ensemblplants* [7]. The library includes cDNA, CDS and ncRNA sequences (plants.ensembl.org/info/website/ftp/index.html). We also used the RNA-seq database of *T*. *turgidum* ssp *dicoccoides* [8] found at (https://wheat.pw.usda.gov/GG3/wildemmer, http://wewseq.wixsite.com/consortium).

### Computer-assisted analysis

#### Retrieval of MITE insertions

The sequences of 35 previously characterized MITE families belonging to the *Stowaway*, *Tourist*, *Mutator* and unknown superfamilies were retrieved from the 4 genome drafts, using MITE analysis kit (MAK) software (http://labs.csb.utoronto.ca/yang/MAK/) [35, 36]. MAK is a homology-based software, meaning it uses a consensus MITE sequence as query and the BLASTN algorithm with global alignment. The publicly available consensus sequence of each MITE family (TREP database at http://wheat.pw.usda.gov/ggpages/Repeats/ and GIRI database at http://www.girinst.org/repbase/update/browse.php) was used as an input (query sequence) in the MAK software. We used an e-value of 1e^-3^ and an end mismatch tolerance of 20 nucleotides. In addition, flanking sequences (500 bp from each end) were retrieved, together with each MITE insertion, to molecularly characterize the insertion sites. A rice-specific MITE called *mPing* served as a negative control in the MAK analysis. No *mPing-*related sequences were retrieved from any of the 4 genome drafts. Redundant sequences were detected with NCBI BLAST+ software [37] standalone version (https://blast.ncbi.nlm.nih.gov/Blast.cgi?CMD=Web&PAGE_TYPE=BlastNews#1). The BLASTN function for insertion duplicates (MAK errors) was used for comparing each family sequence and exclusion of the paired element from each couple of sequences that were found to share 100% identity. It is important to mention that in this analysis, we considered truncated elements (at one of the terminal sequences) as being nearly intact elements.

#### Statistical analysis, sequence conservation, and target site preference

The Galaxy online platform was employed for sequence characterization and statistical analyses [38, 39], using the “Fasta manipulation” and “Statistics” programs. Calculation of average TE lengths for each MITE family was done using the ‘Compute Sequence Length’ function that calculates nucleotide sequences length in a FASTA file, the ‘Count’ function that calculates the copy numbers of a TE family and the ‘Summary Statistics’ function that calculates the summation, mean and standard deviation of sequence lengths in Galaxy. Output files were edited with the “regular expression” function of Textpad 7.4 to separate an element sequence from its flanking sequences before further analysis.

Levels of sequence conservation in each MITE family were analyzed using the MAFFT 7.245 package for multiple sequence alignment, using default parameters and output as fasta files [40], and DNAsp 5.10.1 software [41]. MAFFT creates a multiple sequence alignment for a transposable element family in a FASTA format file which was then analyzed with DNAsp to find sequence similarities and conserved regions, using the ‘haplotype diversity’ function and viewing the sequence alignments.

Analysis of target site preference for each TE family was done using the publicly available online WebLogo 3.0 package [42]. Target site duplications were retrieved using MAK software during TE analysis, adjusted to fit the same length (by adding Ns) for each family and then analyzed at the WebLogo website (http://weblogo.threeplusone.com/create.cgi). The WebLogo 3.0 software generates graphical presentations for each transposable element family sequence and target site preferences. Each logo comprises a stack of letters (different nucleotides) for each nucleotide position in a sequence. The height of each letter in the stack represents its relative frequency at the specific position, while stack width represents the relative fraction of valid nucleotides at that position.

#### Annotation of MITE-flanking sequences

Annotation of MITE flanking sequences was performed using the complementary-DNA (cDNA), coding sequences (CDS) and non-coding RNA (ncRNA) databases of wheat species taken from *EnsemblPlants* (http://plants.ensembl.org/index.html) and the TE databases of plant transposable elements taken from TREP (botserv2.uzh.ch/kelldata/trep-db/index.html). Annotation was performed using BLAST+ standalone version 2.2.3 with an e-value of 1e-10. The merged 5’ and 3’ flanking sequences, as well as the transposable elements themselves, were used as query against the mentioned databases. The best annotation hit of each flanking sequence was chosen to determine the specific protein product, ncRNA or TE family. Furthermore, genes that contain TE sequences were analyzed for association of TEs with wheat genes (i.e., located in an intron, exon, up to 100 bp downstream or upstream to a given gene), using the *EnsemblPlants* database. Association between a MITE and gene was considered when the MITE was inserted into or adjacent to (up to 100 bp upstream or downstream) the gene.

### Plant material

We used various *Triticum* and *Aegilops* species, and synthetic allohexaploids (S1 Table), namely the A genome (*T*. *urartu*, 2 accessions; *T*. *monoccocum*, 1 accession), the B genome (*Ae*. *speltoides*, *Ae*. *searsii*, *Ae*. *sharonensis* and *Ae*. *longissima*), the D genome (*Ae*. *tauschii*, 3 accessions), and the allopolyploid species *T*. *turgidum* (3 accessions of *dicoccoides* and 3 of *durum*) and *T*. *aestivum* (3 accessions of bread wheat, and 4 accessions of synthetic generations of ABD, S1-S4 generations) [43]. Accessions details are found in S1 Table.

### DNA isolation and site-specific PCR analysis

DNA was extracted from young leaves (4 weeks post-germination) using a DNeasy plant kit (Qiagen). PCR validation of the existence of the newly discovered MITE family (*Inbar*) was performed using primers, designed using PRIMER3 version 4.0.0 (bioinfo.ut.ee/primer3/) from flanking sequences of the insertion. Each PCR reaction contained 12.5 μl ultrapure water (Biological Industries), 2 μl of 10xC *Taq* DNA polymerase buffer (EURX), 1.5 μl of 25 mM MgCl_2_ (EURX), 0.8 μl of 2.5 mM dNTPs, 0.2 μl *Taq* DNA polymerase (5 U μl-1, EURX), 1 μl of each site-specific primer (50 ng/μl) and 1 μl of template genomic DNA (approximately 50 ng/μl). The PCR conditions were 94°C for 3 min, 30 cycles of 94° C for 1 min, 60°C for 1 min and 72°C for 1 min, and 72°C for 3 min. For sequence validation, PCR products were extracted from agarose gels using a QIAquick PCR Purification Kit (Qiagen) and subjected to sequencing.

Primer sequences can be found in S6 Table.

## Results

### Assessing MITE composition and chromosomal distribution in diploid and polyploid genomes

In addition to the genome drafts of two diploid genome donors (A and D genomes), the updated genome drafts of the polyploid wild emmer wheat and bread wheat facilitated detailed analyses of the content, chromosome location and distribution of MITE families and allowed comparative analysis of MITE composition among *Triticum* and *Aegilops* species from different ploidy levels in the present study. The consensus sequences of all characterized MITE families in *Triticum* and *Aegilops* species were used as queries in MAK software to retrieve MITE insertions, together with flanking sequences (500 bp from each side), from the genome drafts of *T*. *urartu* (donor of genome A), *Ae*. *tauschii* (donor of genome D), *T*. *turgidum* ssp. *dicoccoides* (genome AB, wild emmer, the “mother” of wheat) and *T*. *aestivum* (genome ABD). We included *mPing*, a rice-unique MITE family [14], in this analysis as a negative control; no *mPing* sequences were retrieved from any of the *Triticum* or *Aegilops* genome drafts. In addition, all of the retrieved sequences corresponded to nuclear DNA, with no sequences being retrieved from the mitochondrial and/or chloroplast genomes.

### MITE composition in the *T*. *urartu* genome

**Fig 1:**
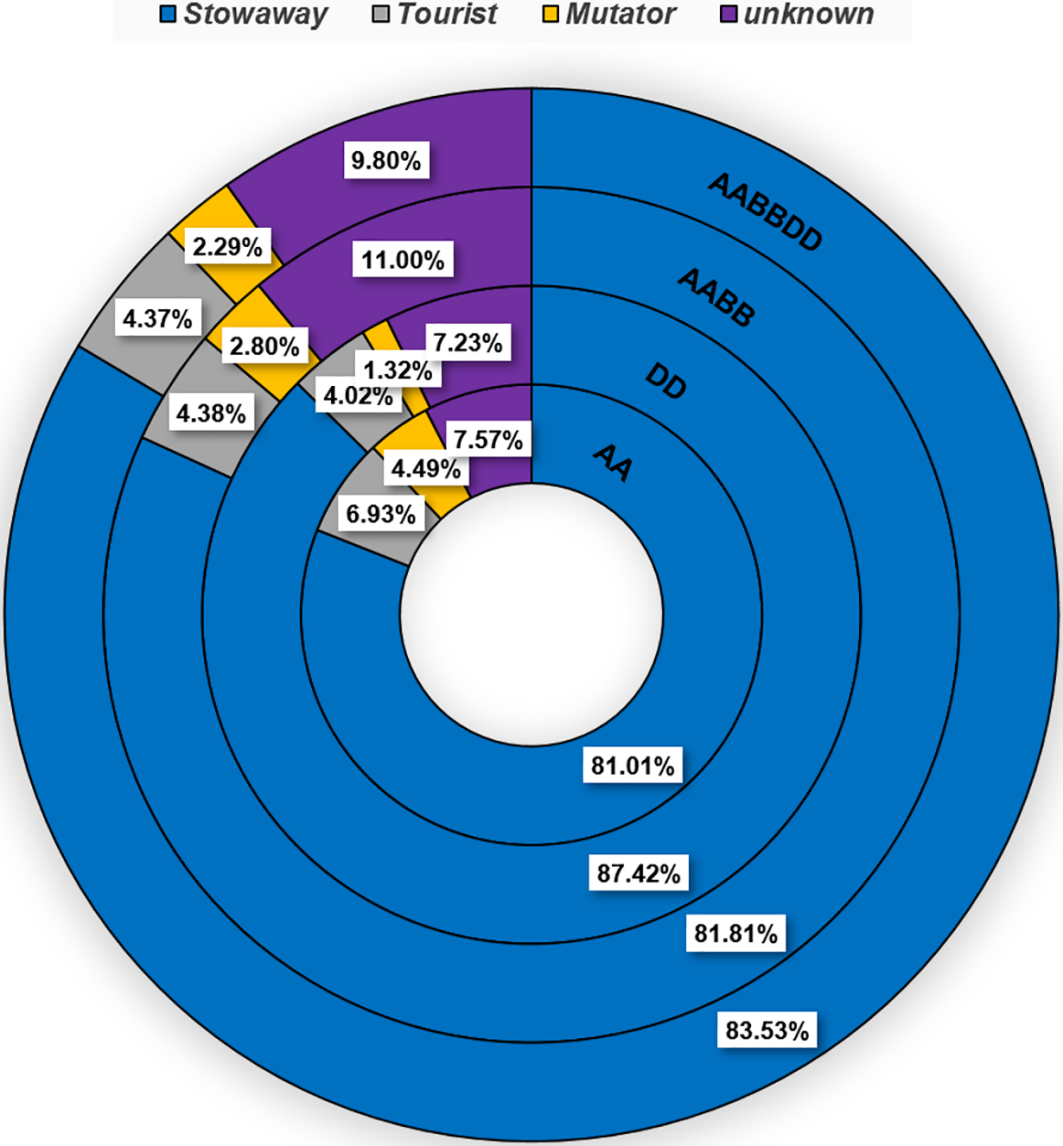
Proportions of MITE insertions by superfamilies in *Triticum* and *Aegilops* genomes. From outer to inner circles: *T*. *aestivum* (AABBDD), *T*. *turdigum* ssp *dicoccoides* (AABB), *Ae*. *tauschii* (DD) and *T*. *urartu* (AA). Percentages denote the fraction of each superfamily from the total number of MITE insertions.

We retrieved 15,513 insertions belonging to 35 MITE families (Table 1) from the *T*. *urartu* genome using MAK software, which account for ~2.62 Mbp (0.053%) of the total ~4,940 Mbp [34]. Most of the retrieved MITE families (19 of the 35 families) belong to the *Stowaway* superfamily (12,567 insertions or 81% of total MITE insertions; Fig 1). However, 5 families belong to the *Tourist* superfamily (1,075 insertions or 6.93% of total MITE insertions), 7 families belong to the *Mutator* superfamily (696 insertions or 4.5% of total MITE insertions) and 4 families belong to unknown superfamilies (1,175 insertions or 7.57% of total MITE insertions). The *Thalos* family (*Stowaway* superfamily) was found to present the highest copy numbers (5249 insertions), while the *Polyphemus* family (*Stowaway* superfamily) was found to present the lowest copy numbers (only 1 insertion). Other families presenting relatively high copy numbers were *Athos* (2,314, *Stowaway* superfamily), *Pan* (1,407, *Stowaway* superfamily), *Belus* (929, unknown superfamily), *Icarus* (694, *Stowaway* superfamily), *Hades* (643, *Stowaway* superfamily), and *Minos* (636, *Stowaway* superfamily).

**Table 1:** Characterization of MITE families in four wheat species: Consensus element size, target site preference (TSD), copy number and % of total MITE content.

Family name	Super family name^1^	Consensus Element Size (bp)^2^	Target Site Preference (TSD)^3^	Copy number				% of total MITEs			
				*T*. *aestivum*	*T*. *turgidum*	*Ae*. *tauschii*	*T*. *urartu*	*T*. *aestivum*	*T*. *turgidum*	*Ae*. *tauschii*	*T*. *urartu*
*Thalos*	*Stowaway*	158	TA	42321	27946	12557	5249	36.26	39.11	41.38	34.04
*Athos*	*Stowaway*	81	TA	19359	10637	5297	2314	19.47	14.89	17.46	15.01
*Pan*	*Stowaway*	123	TA	14296	11108	3838	1407	11.68	15.54	12.65	9.13
*Icarus*	*Stowaway*	108	TA	6795	4512	1649	694	5.74	6.31	5.43	4.50
*Hades*	*Stowaway*	92	TA	3656	2635	898	643	4.54	3.69	2.96	4.17
*Eos*	*Stowaway*	344	TC	3264	2150	974	465	1.42	3.01	3.21	3.02
*Xados*	*Stowaway*	112	AG	1937	1425	445	344	2.40	1.99	1.47	2.23
*Minos*	*Stowaway*	236	TA	1253	1022	164	636	1.26	1.43	0.54	4.13
*Aison*	*Stowaway*	215	TA	852	692	115	166	1.04	0.97	0.38	1.08
*Stolos*	*Stowaway*	255	TA	720	509	197	232	0.93	0.71	0.65	1.51
*Fortuna*	*Stowaway*	349	TA	509	440	35	112	0.28	0.62	0.12	0.73
*Oleus*	*Stowaway*	146	TA	407	258	130	106	0.56	0.36	0.43	0.69
*Antonio*	*Stowaway*	104	TA	320	226	84	84	0.40	0.32	0.28	0.55
*Minimus*	*Stowaway*	51	TA	320	206	86	76	0.38	0.29	0.28	0.49
*Tantalos*	*Stowaway*	253	CC	105	73	31	28	0.14	0.10	0.10	0.18
*Phoebus*	*Stowaway*	319	CG	34	26	7	3	0.05	0.04	0.02	0.02
*Polyphemus*	*Stowaway*	237	TC	22	11	8	1	0.02	0.02	0.03	0.01
*Jason*	*Stowaway*	256	TA	18	10	7	4	0.02	0.01	0.02	0.03
*Orpheus*	*Tourist*	272	TAA	1912	1474	299	546	2.36	2.06	0.99	3.54
*Kerberos*	*Tourist*	285	TA	1594	808	689	100	1.04	1.13	2.27	0.65
*Coeus*	*Tourist*	273	TTA	777	612	53	163	0.93	0.86	0.17	1.06
*Xenon*	*Tourist*	305	AG	556	422	122	221	0.74	0.59	0.40	1.43
*Victor*	*Tourist*	276	GCA	194	106	57	45	0.20	0.15	0.19	0.29
*Gerald*	*Mutator*	345	AAAAATTAA	1119	925	191	401	1.14	1.29	0.63	2.60
*Rhea*	*Mutator*	561	TACAAAAAA	473	346	124	194	0.51	0.48	0.41	1.26
*Spring*	*Mutator*	223	GGGGAACC	319	270	40	17	0.39	0.38	0.13	0.11
*Argus*	*Mutator*	327	TTTAATTAA	306	280	6	10	0.31	0.39	0.02	0.07
*Vacuna*	*Mutator*	464	TTT	197	141	29	40	0.20	0.20	0.10	0.26
*Gabriel*	*Mutator*	407	CCTC	16	14	5	7	0.01	0.02	0.02	0.05
*Belus*	*unknown*	173	CATG	8522	6114	2014	929	4.56	8.56	6.64	6.03
*Keres*	*unknown*	71	CGGTCCG	640	510	93	100	0.47	0.71	0.31	0.65
*Gorgon*	*unknown*	56	GC	217	123	64	53	0.35	0.17	0.21	0.34
*Inbar*	*unknown*	58	TA	1911	1846	24	93	1.66	2.36	0.08	0.60
*Remus*	*Mutator*	829	CG	211	213	7	27	0.17	0.30	0.02	0.18
*Marius*	*Stowaway*	691	TA	15	11	3	3	0.02	0.02	0.01	0.02
*Murray*	*Mutator*	937	TACTGCTCC	2	1	0	0	0.00	0.00	0.00	0.00
Total				115169	78102	30342	15513				

^1^Based on: http://wheat.pw.usda.gov/ITMI/Repeats

^2^Based on the TREP database (http://wheat.pw.usda.gov/ggpages/Repeats/) and the GIRI database (http://www.girinst.org/repbase/update/browse.php)

^3^see S1 Text

*Stowaway* and *Tourist* MITEs in plants are known to be short length elements (100–500 bp), while *Mutator* MITEs tend to be longer (100–700) [18]. Our analysis showed that *Stowaway* MITEs had an average length of 214 bp, with element length ranging from around 40 to 691 bp. *Tourist* MITEs lengths ranged around 269–296 bp, with an average length of 278 bp. *Mutator* MITEs lengths were found to be longer than those of *Stowaway* and *Tourist* MITEs, ranging from around 221 to 826 bp, with an average length of 446 bp. The 4 MITE families that belong to unknown superfamilies had very short lengths, ranging from around 51 to 171 bp.

**Fig 2:**
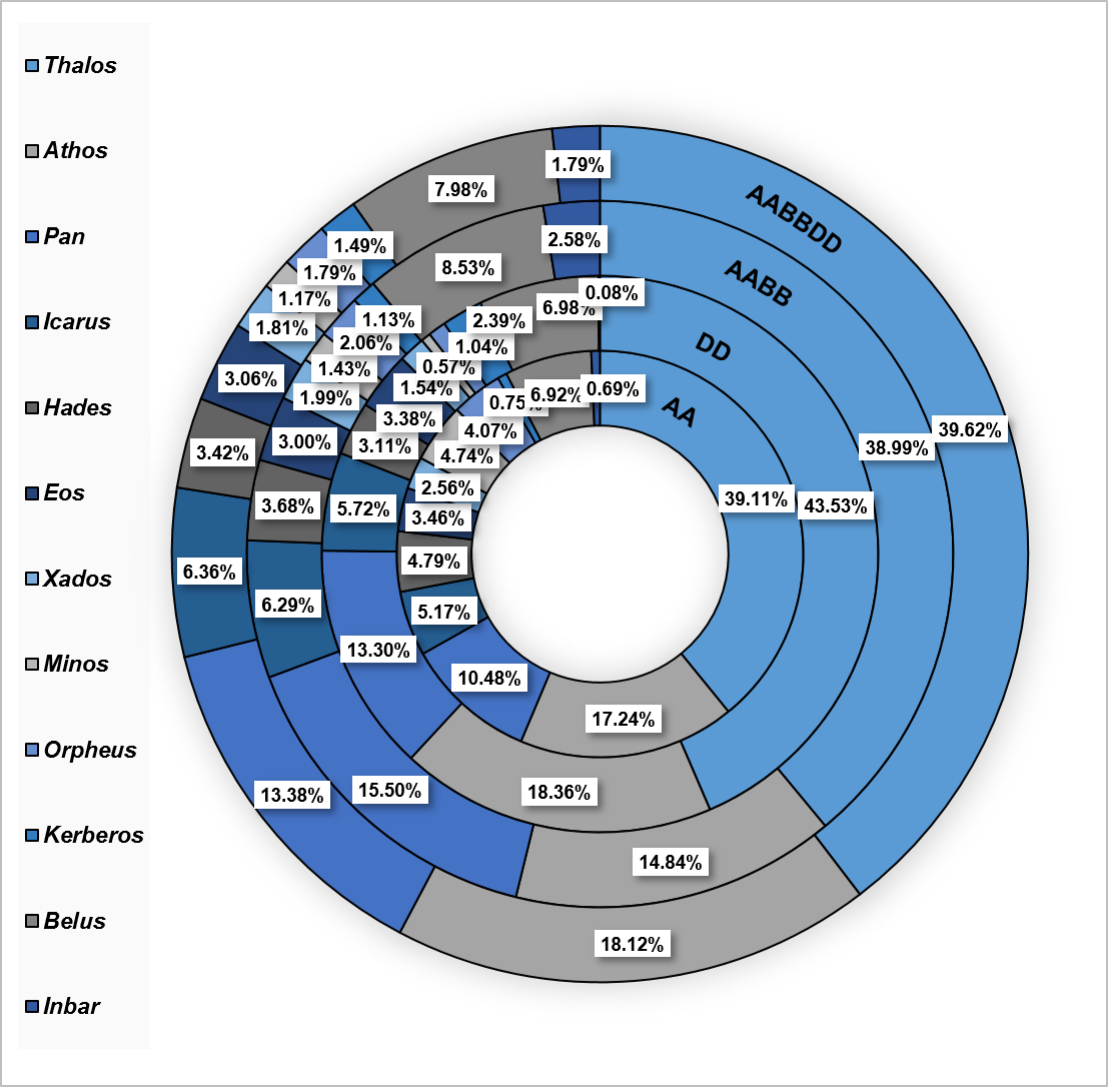
Proportion of MITE insertions by families in *Triticum* and *Aegilops* genomes. From outer to inner circles: *T*. *aestivum* (AABBDD), *T*. *turdigum* ssp *dicoccoides* (AABB), *Ae*. *tauschii* (DD) and *T*. *urartu* (AA). MITE families are indicated by different colours (left). Percentages denote the fraction of each family from the total number of MITE insertions. Note that only values for the 12 most abundant families (according to abundance in the polyploids) are shown due to space limitations.

We also analyzed the relative fraction of each MITE family from the total number of insertions found in the genome (Table 1, Fig 2). The *Thalos* family (*Stowaway* superfamily) had the highest fraction, corresponding to 34% af all MITEs, followed by *Athos* (*Stowaway* superfamily; 15%), *Pan* (*Stowaway* superfamily; 9%), *Belus* (unknown superfamily; 6%), *Icarus* (*Stowaway* superfamily; 4.5%, *Hades* (*Stowaway* superfamily; 4.1%). The other families presented fractions ranging from 0.006% to 3%. This shows that most MITE insertions within the genome of *T*. *urartu* belong to 3 *Stowaway* families, *Thalos*, *Athos* and *Pan*, which account for approximately 58% of total MITE insertions.

#### MITE composition in the *Ae*. *tauschii* genome

Overall, 30,342 insertions belonging to 35 MITE families (Table 1) were retrieved from the *Ae*. *tauschii* genome draft, which account for ~4.57 Mbp (0.1%) of the total ~4,360 Mbp [44]. The retrieved MITE families included 19 families assigned to the *Stowaway* superfamily (12,012 insertions or 87.4% of total MITE insertions), 5 families of the *Tourist* superfamily (818 insertions or 4% of total MITE insertions), 7 families of the *Mutator* superfamily (394 insertions or 1.32% of total MITE insertions) and 4 families of unknown superfamilies (948 insertions or 7.23% of total MITE insertions) (Fig 1).

*Thalos* presented the highest copy number (12,557 insertions), while *Marius* presented the lowest copy number (3 insertions only). Other families presenting high copy numbers were *Athos* (5,297, *Stowaway* superfamily), *Pan* (3,838, *Stowaway* superfamily), *Belus* (2,014, unknown superfamily), *Icarus* (1,649, *Stowaway* superfamily) and *Hades* (898, *Stowaway* superfamily). Analysis of the relative fraction of each family of insertions from the total number of MITE insertions revealed that the *Thalos* family (*Stowaway* superfamily) had the highest fraction (41.4% of all MITEs), followed by *Athos* (*Stowaway* superfamily; 17.5%), *Pan* (*Stowaway* superfamily; 12.6%), *Belus* (unknown superfamily; 6.6%), *Icarus* (*Stowaway* superfamily; 5.4%) and *Hades* (*Stowaway* superfamily; 3%). Fractions from other families ranged from 0.01% to 3.21% (Fig 2). This showed, similarly to what was found for the *T*. *urartu* genome, that the most abundant families are *Thalos*, *Athos* and *Pan*, which account for approximately 71.5% of total MITE insertions.

#### MITEs composition in the *T*. *turgidum* ssp. *dicoccoides genome*

Overall, 78,102 insertions belonging to 36 MITE families were retrieved from the tetraploid *T*. *turgidum* ssp. *dicoccoides* genome draft (Table 1), which account for ~ 12 Mbp (0.1%) of the total ~ 12,000 Mbp [8]. The retrieved MITE families included 19 families of the *Stowaway* superfamily (63,897 insertions or ~81.8% of total MITE insertions), 5 families of the *Tourist* superfamily (3,422 insertions or 4.38% of total MITE insertions), 9 families of the *Mutator* superfamily (2,190 insertions or 2.8% of total MITE insertions) and 4 families of unknown superfamilies with 8,593 insertions or 11% of total MITE insertions (Fig 1).

*Thalos* had the highest copy number (27,946 insertions), while *Murray* only had a single insertion (Table 1). Other families with high copy numbers were *Pan* (11108, *Stowaway* superfamily), *Athos* (10637, *Stowaway* superfamily), *Belus* (6114, unknown superfamily) *Icarus* (4512, *Stowaway* superfamily), *Hades* (2635, *Stowaway* superfamily), *Eos* (2150, *Stowaway* superfamily), *Xados* (1425, *Stowaway* superfamily) and *Orpheus* (1474, *Tourist* superfamily). Analysis of the relative fraction of each family from the total number of MITEs showed that the *Thalos* family (*Stowaway* superfamily) had the highest fraction, with 35.8% of all MITE insertions, followed by *Pan* (*Stowaway* superfamily;14.2%), *Athos* (*Stowaway* superfamily; 13.6%, *Belus* (unknown superfamily; 7.8%), *Icarus* (*Stowaway* superfamily 5.8%), *Hades* (*Stowaway* superfamily; 3.4%) and the other families, with fractions of 0.001% to 3% (Table 1, Fig 2). The three *Stowaway*-MITE families *Thalos*, *Athos* and *Pan* are the most abundant in the wild emmer wheat genome and account for ~64% of the total MITE insertions.

#### Chromosomal distribution of MITE insertions in *T*. *turgidum ssp*. *dicoccoides*

**Fig 3:**
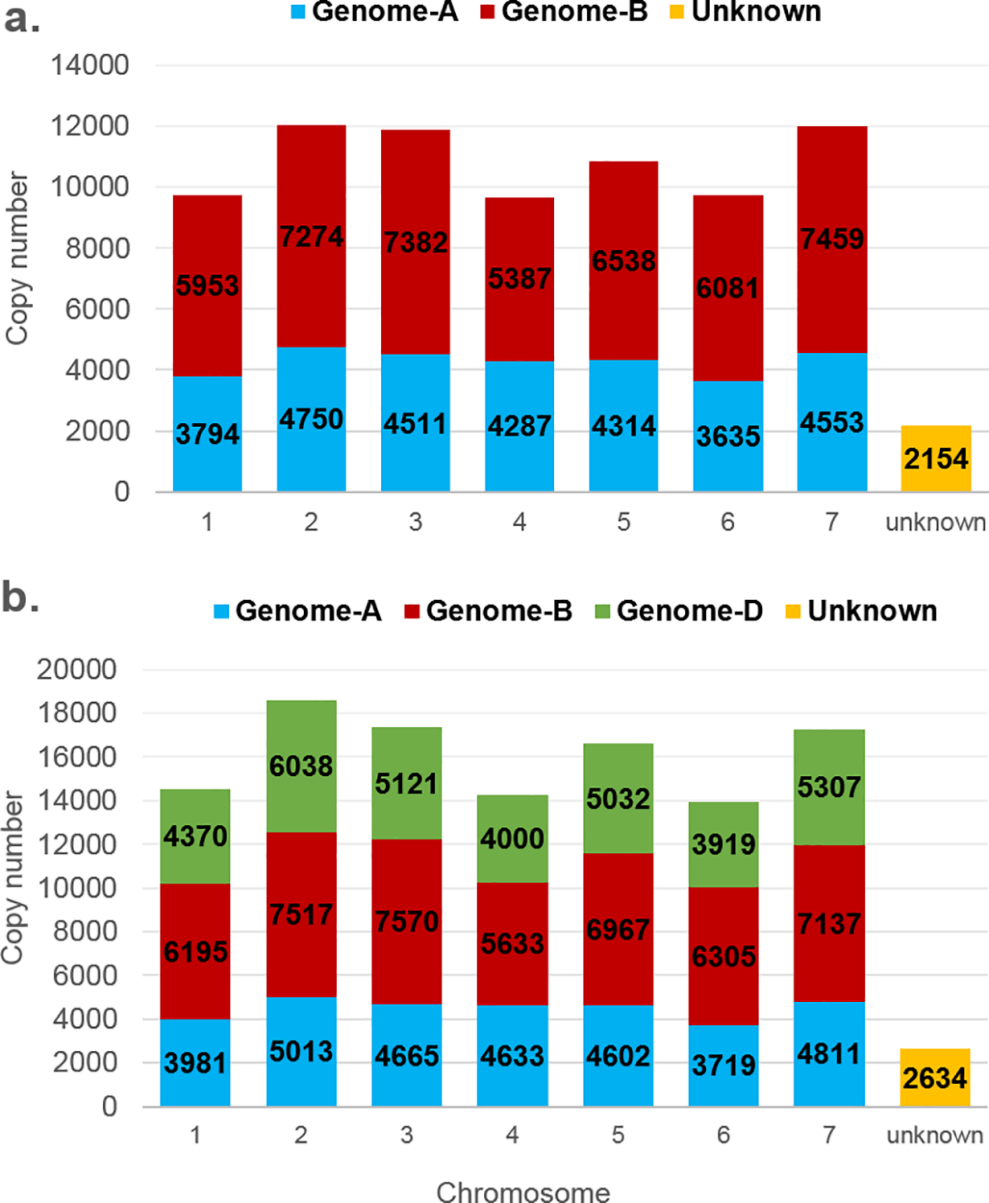
Distribution of MITE insertions within the seven homologous chromosomes. Each chromosome is defined by its genome (AA, BB, and DD, indicated by different colours; top) and number (1–7). **a.** Distribution of MITE insertions within chromosomes of the tetraploid *T*. *turgidum* ssp. *dicoccoides* (genome AABB). **b.** Distribution of MITE insertions within chromosomes of the hexaploid *T*. *aestivum* (genome AABBDD).

The newly available genome draft of emmer wheat (*T*. *turgidum* ssp. *dicoccoides*) facilitated analysis of MITE distribution in the two sub-genomes and 7 homoeologous chromosomes (Fig 3A). We found that 59% of the insertions (46,074 of 78,102 insertions) were located within the B sub-genome, as compared to 38.2% (29,844) insertions located within the A sub-genome (S2 Table). Note that 2.8% (2,154) of the insertions could not be mapped to either sub-genomes, and were thus listed as “unknown” (Fig 3). These data could indicate different proliferation levels of MITEs in the *T*. *dicoccoides* A and B sub-genomes (See S1 Fig for distribution of each family). For example, 62.7% of *Thalos* insertions (17,522) were found in the B sub-genome, 34.67% (9,689) of the insertions were found in the A sub-genome and only 2.63% were found in unidentified genome regions (S1A Fig, S2 Table). However, some families showed a different trend in terms of copy numbers. For example, 80.92% of *Minos* insertions were found in the A sub-genome (827), while only 16.34% of such insertions were found in the B sub-genome (167); 2.74% (28) *Minos* insertions were found in unidentified genome regions (S1H Fig). At the chromosome level, the highest fraction of MITE insertions was 12,024 elements found within group-2 chromosomes. The highest fraction of MITE elements was found within chromosome 7B (7,459 insertions), accounting for 9.5% of total MITE insertions.

#### MITEs composition in the *T*. *aestivum* genome

Overall, 115,169 insertions belonging to 36 MITE families were retrieved from the hexaploid *T*. *aestivum* genome draft (Table 1), which account for ~17.6 Mbp (0.1%) of the total ~17,000 Mbp [7, 9]. The retrieved MITE families included 19 families belonging to the *Stowaway* superfamily (96,203 insertions or 83.53% of total MITE insertions), 5 families belonging to the *Tourist* superfamily (5,033 insertions or 4.37% of total MITE insertions), 9 families belonging to the *Mutator* superfamily (with 2,643 insertions or 2.29% of total MITE insertions) and 4 families belonging to unknown superfamilies (11,290 insertions or 9.8% of total MITE insertions) (Fig 1).

*Thalos* showed the highest copy number (42,321 insertions), while only 2 *Murray* insertions were found. Other families with high copy numbers were *Athos* (19,359, *Stowaway* superfamily), *Pan* (14,296, *Stowaway* superfamily), *Belus* (8,522, unknown superfamily), *Icarus* (6,795, *Stowaway* superfamily), *Hades* (3,656, *Stowaway* superfamily), and *Eos* (3,264, *Stowaway* superfamily). As in the *T*. *turgidum* ssp. *dicoccoides* genome, copy numbers varied remarkably between the different MITE families, with the majority of high copy number families belonging to the *Stowaway* superfamily. Analysis of the relative fraction of each family from the total MITEs population showed that the *Thalos* family (*Stowaway* superfamily) had the highest fraction at ~37%, followed by *Athos* (*Stowaway* superfamily; ~17%), *Pan* (*Stowaway* superfamily; ~12%), *Belus* (unknown superfamily; 7.5%), *Icarus* (*Stowaway* superfamily; 6%), and other families, with fractions spanning 0.01% to ~3% (Table 1, Fig 2). As in the diploid and tetraploid species, the highest copy numbers of MITE families in the hexaploid genome were *Thalos*, *Athos* and *Pan*, which account for approximately 66% of total MITE insertions, indicating that these 3 families contained the most active MITEs throughout wheat evolution.

#### Chromosomal distribution of MITE insertions in *T*. *aestivum*

The updated genome draft of the hexaploid wheat genome (publicly available on *EnsemblPlants* since June, 2016) allowed us to analyze the distribution of MITE insertions in the three sub-genomes and 7 homologous chromosomes of wheat (Fig 3B). We found that 41% of MITE insertions were located within the B sub-genome (47,324 of 115,169 total MITE insertions), as compared to 29.3% (33,787) of the insertions in the D sub-genome, 27.3% (31,424) of the insertions in the A sub-genome and 2.28% insertions being unmapped (S2 Table). Most MITE families presented a sub-genome-specific proliferation profile (see S2 Fig for distribution of each family), meaning that they were not equally distributed across all three sub-genomes. For example, 41% (17,202) of *Thalos* insertions were found in the B sub-genome, as compared to 34.5% (14,600) of such insertions in the D sub-genome and 23% (9,699) in the A sub-genome (S2A Fig, S2 Table). Another such example was the *Minos* family, where ~70% (871) of the insertions were found in the A sub-genome, as compared to ~15% (194) in the D sub-genome) and only 12% (151) in the B sub-genome (S2H Fig).

At the chromosome level, the highest fraction of MITE insertions were the 17,356 elements found within group-3 chromosomes, which account for 15% of all elements. At the combined chromosome and genome levels, the highest fraction of MITE elements was found within chromosome 3B (7,570 insertions, accounting for 6.5% of total MITE insertions), which is also the largest wheat chromosome (995 Mbp) [45] and was the first fully assembled chromosome of wheat [9].

### Comparative analysis of MITE composition in *Triticum* and *Aegilops* genomes

All MITE families found in the hexaploid *T*. *aestivum* and the tetraploid *T*. *turgidum* ssp. *dicoccoides* were also found within the diploid *Ae*. *tauschii* and *T*. *urartu* genomes, except for the *Murray* family (*Mutator*) that presented only 1 and 2 copies in the hexaploid and the tetraploid genomes, respectively (Table 1). The *Stowaway* superfamily was the most abundant (~80% of insertions) MITE superfamily in the wheat genome. The relative fraction of *Stowaway* MITE insertions is 81.01% in *T*. *urartu*, 81.81% in *T*. *turgidum ssp*. *dicoccoides*, 87.42% in *Ae*. *tauschii*, and 83.53% in *T*. *aestivum*. The other analyzed families belong to *Tourist* (5 families), *Mutator* (8 families) or to unknown (4 families) superfamilies. The relative fraction of *Tourist* MITE insertions varied from 4.02% in *Ae*. *tauschii*, 4.37% in *T*. *aestivum*, and 4.38% in *T*. *turgidum* ssp. *dicoccoides* to 6.93% in *T*. *urartu*. *Mutator* insertion fractions varied from 1.32% in *Ae*. *tauschii*, 2.29% in *T*. *aestivum*, and 2.8% in *T*. *turgidum* ssp. *dicoccoides* to 4.49% in *T*. *urartu*. The unknown superfamilies insertion fractions varied from 7.23% in *Ae*. *tauschii*, 7.57% in *T*. *urartu*, and 9.8% in *T*. *aestivum* to 11% in *T*. *turgidum* ssp. *dicoccoides* (Fig 1). In all four species, the *Thalos* family presented the highest copy number of all MITE families examined.

**Fig 4:**
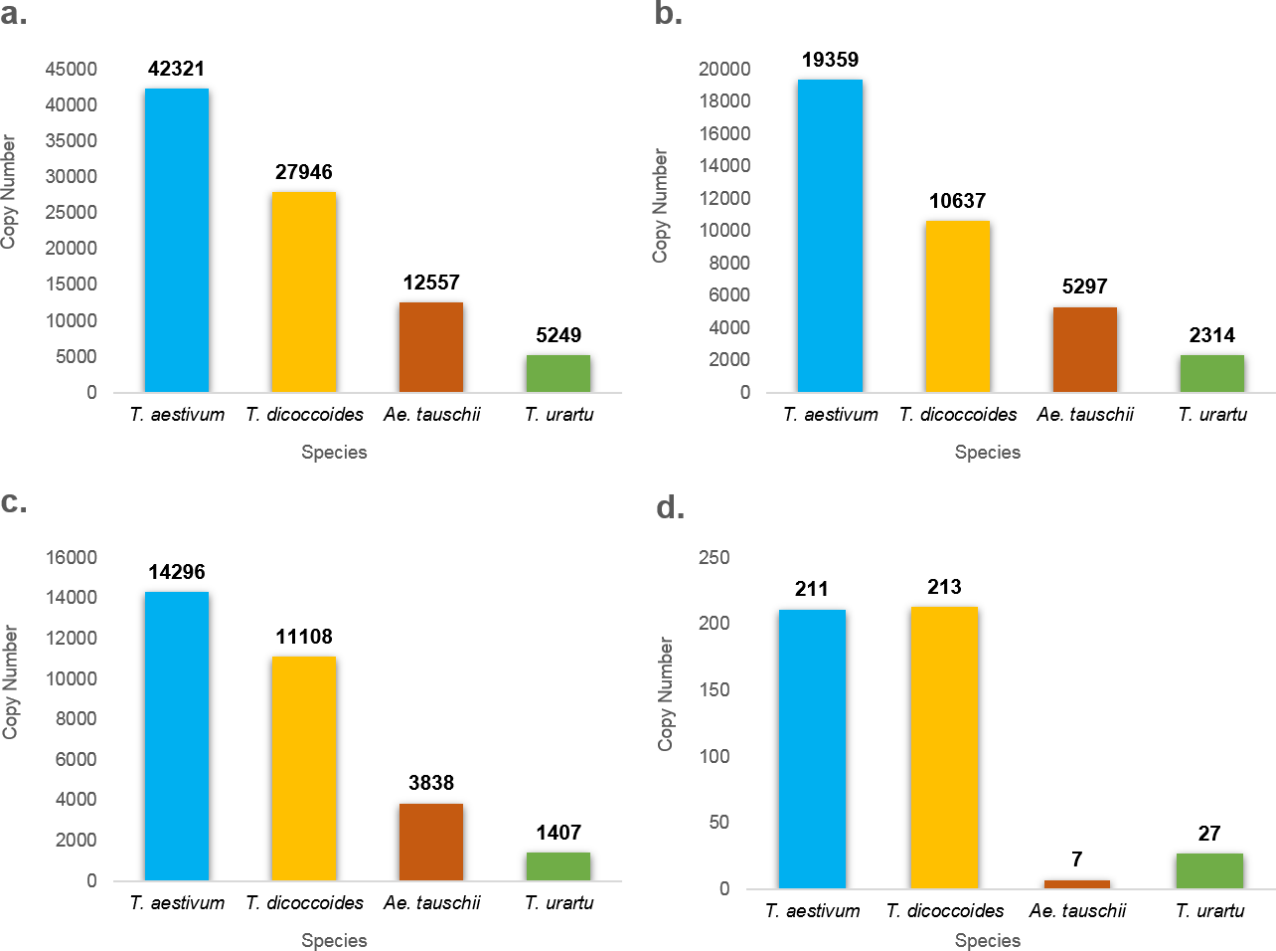
Copy number of four MITE families in *Triticum* and *Aegilops* genomes. Copy numbers are indicated on top of each bar. **a.** *Thalos* (*Stowaway*), **b.** *Athos* (*Stowaway*)**, c.** *Pan* (*Stowaway*)**, d.** *Oleus*.

We observed variations in MITE insertion copy numbers between polyploid and diploid species (Table 1, S3 Fig). Almost all MITE families show patterns of variation that can be explained either by differences in genome size and composition or as the result of different activity levels [30]. Common insertion analysis (comparison of TE insertions with their flanking sequences) of four MITE families (*Thalos*, *Athos*, *Pan* and *Belus*) comparing hexaploid and tetraploid insertions showed that only ~30–47% (34–41%, 30–36%, 35–41% and 37–47% in each chromosome of a family, respectively) of each family of insertions were common to both polyploids, meaning insertions that were inherited from tetraploid to hexaploid wheat. This means that the other 53–70% of insertions are unique to either tetraploid or hexaploid wheat and might be the result of transpositions or rearrangements, such as recombination or deletion (e.g., deletion of a MITE-containing sequence in the hexaploid would result in a “unique” insertion found in the tetraploid). The *Thalos* family, for example, presented 42,321 insertions in the hexaploid (9,699 in the A sub-genome, 17,202 in the B sub-genome and 14,600 in the D sub-genome), 27,946 insertions in the tetraploid (9,689 in the A sub-genome, 17,522 in the B sub-genome) and 12,557 insertions in D genome donor (3:2:1 ratio; Fig 4A, S2 Table). This variation can be accounted as TE inheritance alone, althouh *Thalos* common insertion analysis showed ~59–66% of such insertions as being unique to either tetraploid or hexaploid wheat. In a previous study [46], we found that Class II transposons display different patterns of cytosine methylation in a synthetic allohexaploid, as opposed to a synthetic allotetraploid. The *Thalos* elements underwent massive hyper-methylation in the S1-S4 generations of the allohexaploid, while hypo-methylation was predominant in the S1-S5 generations of the allotetraploid. Hypo-methylation indicates a potential for rearrangement and possibly activation of *Thalos* elements following the allotetraploidization event. The relatively similar copy number in each sub-genome of the tetraploid and hexaploid suggests that a major part of *Thalos* activity in the A and B sub-genomes occurred following the tetraploidization event, as the copy number in the diploid A donor is much lower (5,249). Nevertheless, since MITEs are class II elements, transposition of elements might have occurred with no proliferation, as was shown for specific cases in a previous study from our lab [1, 47].

A similar pattern of insertions was also seen with *Athos* insertions, where we found 19,359 insertions in *T*. *aestivum*, 10,637 insertions in *T*. *turgidum* ssp. *dicoccoides*, 5,297 in *Ae*. *tauschii* and 2,512 insertions in *T*. *urartu* (~8:4:2:1 ratio; Fig 4B). Another unique pattern was observed in terms of *Pan* insertions, where we found 14,296 insertions in *T*. *aestivum*, 11,108 insertions in *T*. *turgidum* ssp. *dicoccoides*, 3,838 in *Ae*. *tauschii* and 1230 insertions in *T*. *urartu* (~12:9:3:1 ratio; Fig 4C).

*Remus* insertions presented a different pattern. We noted 213 insertions in *T*. *turgidum* ssp. *dicoccoides*, 211 insertions in *T*. *aestivum*, 7 insertions in *Ae*. *tauschii* and 4 insertions in *T*. *urartu* (~53:53:1:1 ratio; Fig 4D). In this case, there is a similar copy number in both polyploid genomes but only a very small amount in the diploid genomes. This finding, combined with the relatively low number of insertions, indicates that the reason for the observed *Remus* pattern is probably the different sizes of the genomes, as it seems that this family has not been too active.

**Fig 5:**
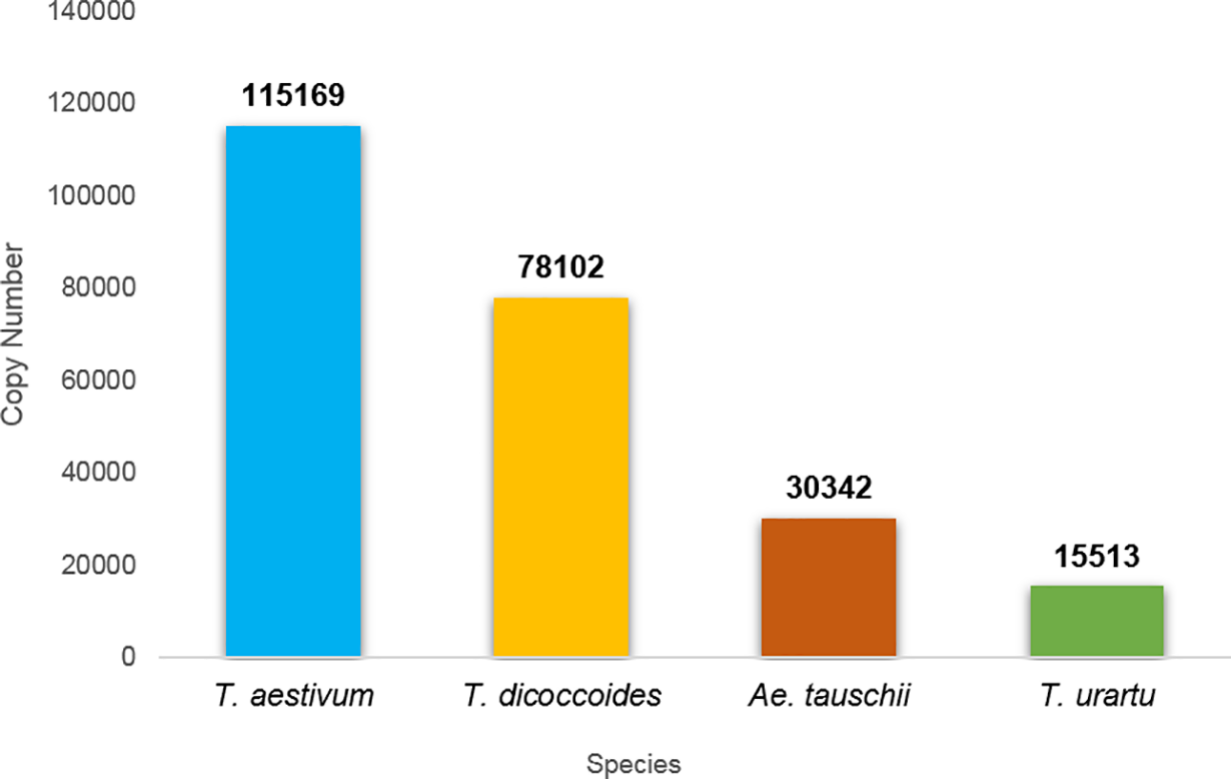
Total copy number of MITE insertions in *Triticum* and *Aegilops* genomes. Copy numbers are indicated on top of each bar.

A high level of MITE sequences was found in the wheat genome together with huge copy number variation among MITE families and among the 4 *Triticum* and *Aegilops* genome drafts (Table 1**)**. A total of 30,366 MITE insertions were found in the *Ae*. *tauschii* genome (D), 15,420 insertions were found in the *T*. *urartu* genome (A), 78,102 insertions were found in the *T*. *turgidum* ssp. *dicoccoides* genome (AB) and 115,169 insertions were found in the *T*. *aestivum* genome (ABD), (Fig 5).

### MITEs associations with genes and TEs

Analysis of MITE sequences with transcribed regions revealed that nearly 52% of the insertions were found within or close to (100 bp range) annotated protein-coding genes (S3 Table), while ~40% of the insertions were within or in close proximity to TE sequences. Of these 40%, 33.54% are found as Class II DNA transposons, mostly in CACTA elements of the *Jorge* family (or 31.49% of all TEs), while 5.34% are found as Class I retrotransposons. Other MITE insertions (~4%) were located in or near non-coding RNA sequences. The remainder (5%) was located in unidentified regions, most probably corresponding to non-coding sequences.

We noticed that many MITE insertions of the *Belus* (99.5%), *Icarus* (88.7%), *Fortuna* (67.27%), *Pan* (57.27%) and *Thalos* (42.45%) families were found within sequences of the Class II element called “*Jorge*”, in different regions of this element. *Jorge* is a large derivative of the CACTA superfamily found in wheat species (*T*. *monococcum*, *Ae*. *tauschii*, *T*. *aestivum*) and is considered to be non-autonomous and non-active, due to lack of transposases coding CACTA elements in the wheat genome [48]. In the case of the *Belus* family, a MITE family comprising ~173 bp-sized sequences assigned to an unknown superfamily, almost all insertions corresponded to *Jorge* elements, mostly at the same position (around positions 4426–4588 of the 15,800 bp sequence). This suggests that proliferation of *Belus* family was due to a past insertion into a *Jorge* element when the *Jorge* family was still active. In one case, *Belus/Jorge* became part of the coding sequence of a gene coding for a nitrate/chlorate transporter (mapped to exon 4 of an *Ae*. *tauschii* gene, acc. F775_11526, *EnsemblPlants*). Orthologous genes found on chromosomes 5A and 5B of the emmer genome (*GrainGenes*, acc. TRIDC1_5B|TRIDC5BG063580.2, Protein NRT1/ PTR FAMILY, acc. TRIDC1_5A|TRIDC5AG059350.2, Protein NRT1/ PTR FAMILY) lack the *Belus/Jorge* sequence, suggesting that domestication of the *Belus/Jorge* element led to this motif becoming a vital part of such genes. Since *Jorge* family is now, however, non-active, it is possible that the large number of MITE insertions found within *Jorge* elements are evidence of a high copy number of *Jorge* family members in the wheat genome.

In previous work in rice, MITEs were found mostly in genic regions or within other MITE sequences [11, 49]. To validate whether MITE sequences appear in transcribed sequences *in vivo*, we retrieved MITE insertions from the *T*. *aestivum* RNA-seq database using MAK software. Overall, 484 MITE-containing transcripts belonging to 364 different genes were retrieved. The most abundant MITE families found in this transcriptome were *Thalos*, *Athos* and *Pan*. Detailed analysis showed that ~70% of the insertions were located within 3’UTRs, ~17% were in 5’ UTRs and ~13% were within the coding region (CDS). In a previous study, we reported on intron retention of *Au* SINE, a non-LTR retrotransposon family that, in a similar manner as MITEs, is highly associated with wheat genes [50]. *Au* SINE was found to cause allelic variation in wheat protein-coding genes, and in some cases, insertions of *Au* SINE in introns led to intron retention and generation of alternative splice variants generating proteins of shorter lengths. *In silico* examination of 100 MITE-containing transcripts (S4 Table) revealed 24 cases of genes presenting alternative splice variants in which some contain MITEs yet others do not. In all cases, the MITE-containing transcript of a certain gene was longer than the other transcripts, altough protein size was not necessarily larger. In some cases, the protein retained the same sequence and size as encoded by all splice variants, while in other instances, the MITE-containing variant led to the generation of a shorter or a longer protein. For example, gene accession TRIAE_CS42_1AL_TGACv1_002619_AA0043720 (uncharacterized protein, *EnsemblPlants*) presents two splice variants, with one transcript lacking the MITE insertion being 1184 bp-long and coding for a 253 residue-containing protein and the other transcript being 1470 bp-long, containing an *Orpheus* insertion in its 3’ UTR and coding for a 305 residue-containing protein.

In addition to the bread wheat transcriptome, we analyzed the wild emmer wheat transcriptome and retrieved 164 MITE-containing transcripts derived from 72 genes, with 31 of these insertions being found, at least partially, in the CDS (~19%).

### *Inbar*, a new and unique *Stowaway*-like MITE family in wheat

**Fig 6:**
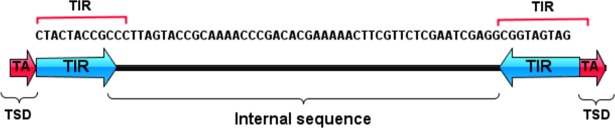
Schematic representation of *Inbar* consensus sequence. The sequences of the TIRs (blue) and the TSDs (red) are indicated.

**Fig 7:**
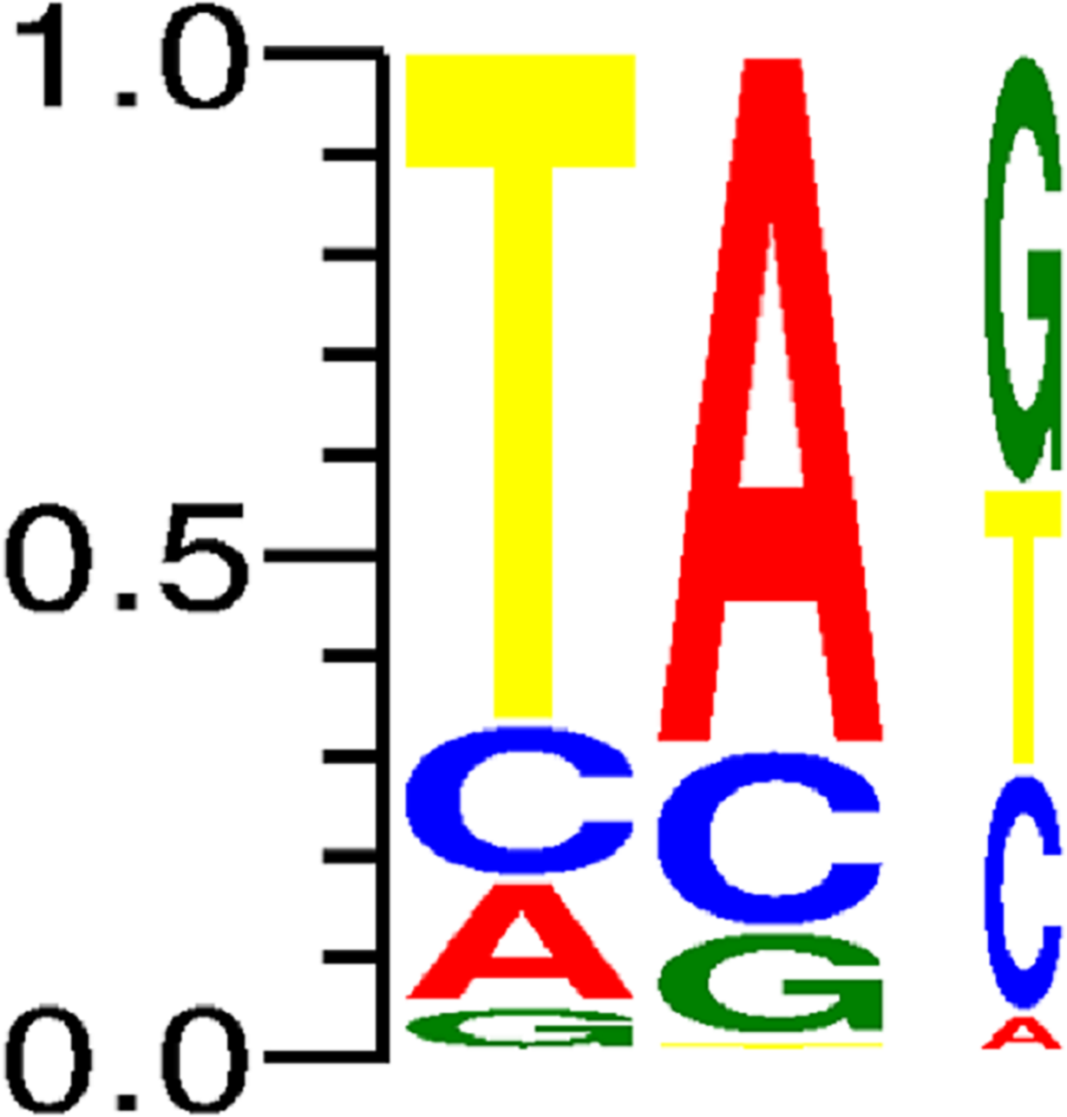
Target site preference (TA) of *Inbar*. Created by the WebLogo 3.0 package, based on MAK data output of target site duplications from all four *Triticum* and *Aegilops* genomes.

**Fig 8:**
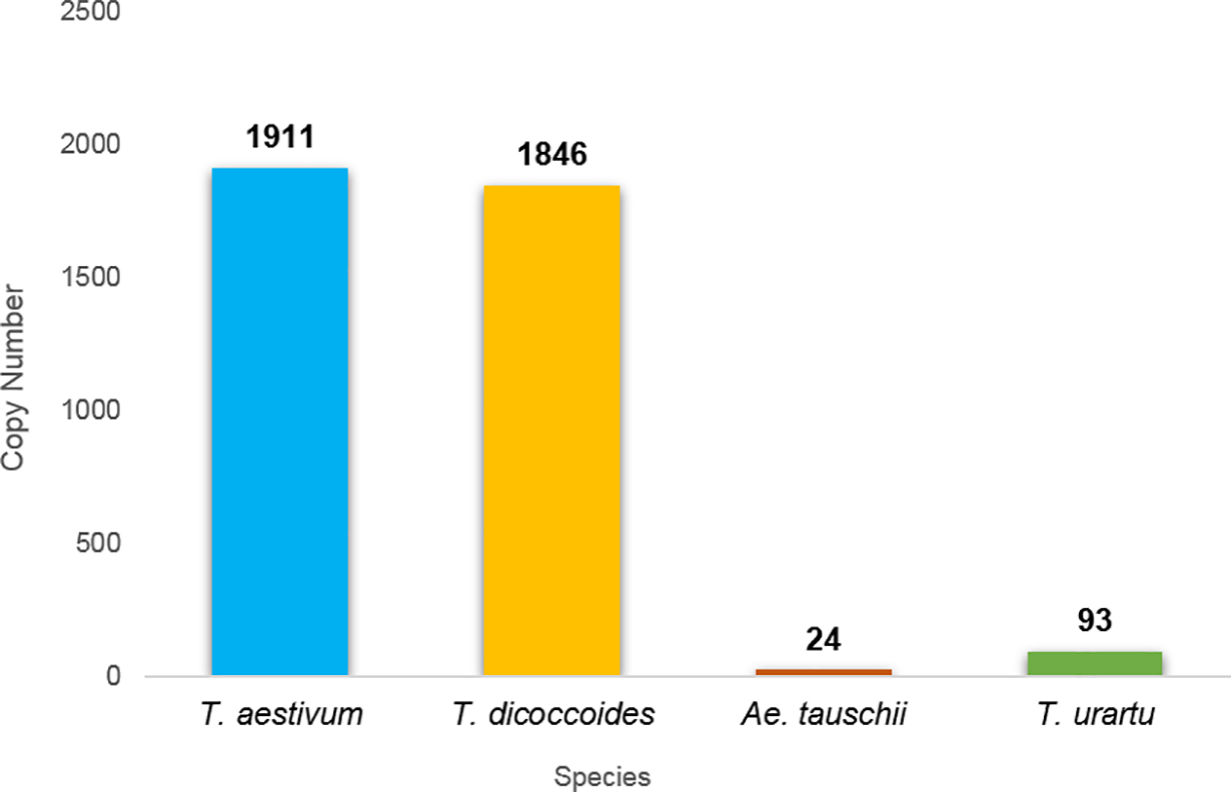
Copy number of the *Inbar* family in *Triticum* and *Aegilops* genomes. Copy numbers are indicated on top of each bar.

**Fig 9:**
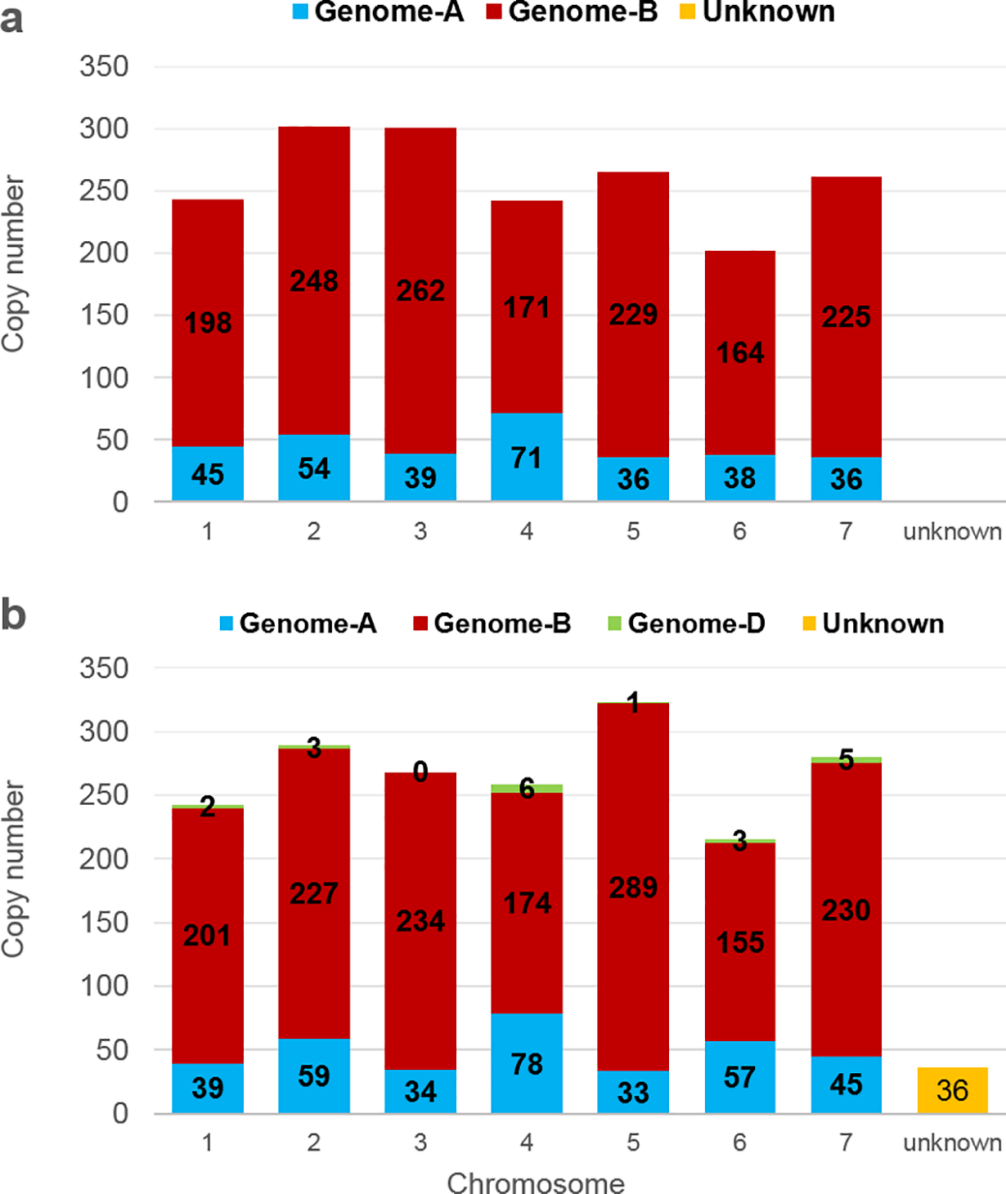
Distribution of *Inbar* insertions within the seven homologous chromosomes. Each chromosome is defined by its genome (AA, BB, and DD, indicated by different colors—top) and numbers (1–7). **a.** Distribution of MITE insertions within tetraploid *T*. *turgidum* ssp. *dicoccoides* (genome AABB) chromosomes. **b.** Distribution of MITE insertions within hexaploid *T*. *aestivum* (genome AABBDD) chromosomes.

Computer-assisted analysis revealed an unfamiliar sequence in chromosome 5B, 68 bp in length, containing TIRs of 11 bp and creating “TA” target site duplication, possibly indicative of an unidentified *Stowaway*-like MITE (Figs 6 and 7). BLAST analysis revealed that this MITE sequence is unique to wheat, as we were not able to detect it in other plant genomes. To assess the composition of *Inbar* in *Triticum* and *Aegilops* species, we used its sequence as query in MAK software and retrieved similar copies from the four genome drafts. Overall, 93 insertions were found in the *T*. *urartu* genome, 24 *in Ae*. *tauschii*, 1,846 in *T*. *turgidum* ssp *dicoccoides* and 1,911 in *T*. *aestivum* (Fig 8). Distribution analysis of the polyploid genomes revealed that *Inbar* elements were predominant in the B sub-genome (81% of the insertions in *T*. *turgidum* ssp *dicoccoides* and 79% of the insertions in *T*. *aestivum*; Fig 9). The massive copy number variation between the diploid and polyploid species indicates that *Inbar* might have been transpositionally activated following the process of allotetraploidization.

Annotation analysis of *Inbar* flanking sequences showed that ~90% of the insertions were in retrotransposon sequences, 7.42% were associated with protein-coding genes, 2.22% were inserted in Class II TEs, and the remaining were associated with non-coding RNA sequences or non-coding DNA (0.49% and 0.21%, respectively) (S5 Table). These data indicate that *Inbar* insertion occurred preferentially within retrotransposon sequences, explaining why it was not identified previously. Interestingly, 57.65% of *Inbar* insertions into retrotransposons were associated with the LTR-*Copia* superfamily and specifically with the sequences of the *Inga* and *Eugene* families, although such insertions were found at different locations within these elements.

**Fig 10:**
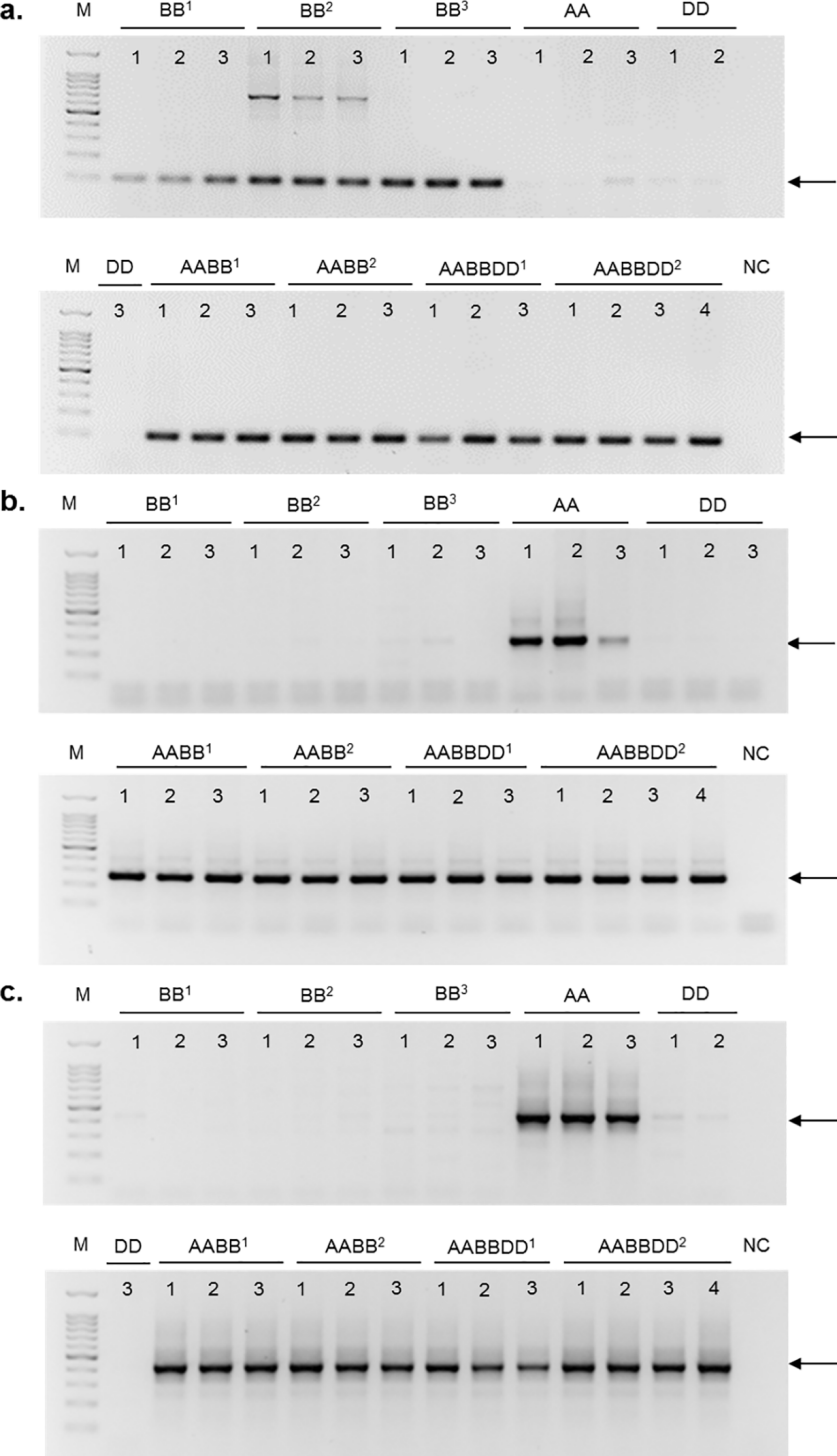
Site-specific PCR analysis using primers raised against *Inbar* flanking sequences. The arrows denote the expected PCR product, “M” denotes size markers, and “NC” denotes a negative control (when ddH_2_O served as PCR template). The PCR analysis was performed with DNA templates of the following accessions: BB^1^ = *Ae*. *searsii*, BB^2^ = *Ae*. *speltoides*, BB^3^ = *Ae*. *sharonensis* / *Ae*. *longissima*, AA = *T*. *urartu* / *T*. *monoccocum*, DD = *Ae*. *tauschii*, AABB^1^ = *T*. *turgidum* ssp *dicoccoides*, AABB^2^ = *T*. *turgidum* ssp *durum*, AABBDD^1^ = *T*. *aestivum*, AABBDD^2^ = synthetic generations of *T*. *aestivum*—S1, S2, S3, S4 (hybdriziation of *Ae*. *tauschii* and *T*. *turgidum* ssp *durum)*. **a.** *Inbar* insertion in a gene located in the BB genome of *Ae*. *searsii*, *Ae*. *speltoides*, *Ae*. *sharonensis*, *Ae*. *longissima*, *T*. *turgidum* ssp *dicoccoides*, *T*. *turgidum* ssp *durum* and *T*. *aestivum* (gene acc. TRIAE_CS42_2BL_TGACv1_129533_AA0387420, *EnsemblPlants*). PCR product size: 101 bp. **b.** *Inbar* insertion in a gene located in the AA genome of *T*. *urartu*, *T*. *turgidum* ssp *dicoccoides*, *T*. *turgidum* ssp *durum* and *T*. *aestivum* (gene acc. TRIAE_CS42_6AL_TGACv1_471379_AA1507990, *EnsemblPlants*). PCR product size: 258 bp. **c.** *Inbar* MITE insertion in a gene located in the AA genome of *T*. *urartu* (gene acc. TRIUR3_10383, *EnsemblPlants*), *T*. *turgidum* ssp *dicoccoides*, *T*. *turgidum* ssp *durum* and *T*. *aestivum* (gene acc. TRIAE_CS42_2AS_TGACv1_113196_AA0352860, ribulose bisphosphate carboxylase small chain *EnsemblPlants*). PCR product size: 426 bp.

We validated our computer-assisted analysis by site-specific PCR analysis using primers designed against *Inbar* flanking sequences. For additional validation, the PCR products were also sequenced. We analyzed *Inbar* insertions associated with genes in three scenarios. In the first case, we considered *Inbar* insertion within an intron of a *T*. *aestivum* gene located on 2B chromosome (identified *in silico* as accession TRIAE_CS42_2BL_TGACv1_129533_A0387420, *EnsemblPlants*, S5 Table). This gene is also found in the genome of B diploids (*Ae*. *searsii*, *Ae*. *speltoides*, *Ae*. *sharonesis* and *Ae*. *longisima*) and polyploids (*T*. *turgidum* ssp *durum*, *T*. *turgidum* ssp. *dicoccoides* and *T*. *aestivum*), meaning that it is probably an insertion unique to the B genome, inherited by the polyploids (Fig 10A). In addition, we addressed an *Inbar* insertion found downstream to a *T*. *aestivum* gene of unknown function located on 6A chromosome (identified in silico as accession TRIAE_CS42_6AL_TGACv1_471379_A1507990, S5 Table). PCR analysis showed this *Inbar* insertion to be found in *T*. *urartu* and in the polyploid genomes (*T*. *turgidum* ssp *durum*, *T*. *turgidum* ssp. *dicoccoides* and *T*. *aestivum*; Fig 10B), indicating this insertion as being unique to the A genome. Finally, an *Inbar* insertion found in an intron of a gene coding for ribulose bisphosphate carboxylase small chain, identified *in silico* in chromosome 2A in *T*. *urartu* (acc. TRIUR3_10383, *EnsemblPlants*, S5 Table), *T*. *turgidum* ssp. *dicoccoides* (acc. TRIDC2BG008560.1, *GrainGenes*) and *T*. *aestivum* (TRIAE_CS42_2AS_TGACv1_113196_A0352860, *EnsemblPlants*), was considered. PCR analysis showed that this *Inbar* insertion is found in *T*. *urartu* and the polyploid genomes (*T*. *turgidum* ssp *durum*, *T*. *turgidum* ssp. *dicoccoides* and *T*. *aestivum*; Fig 10C), indicating it to be specific to genome A. PCR analysis suggests these *Inbar* insertions into genes are ancient, dating back to the divergence of the A, B and D diploid species, an event that transpired ~4 MYA.

## Discussion

Allopolyploidization is considered stress on the plant genome, since this event is followed by massive genetic and epigenetic rearrangements, causing the new genome to act as a diploid, both cytologically, as demonstrated by pairing during meiosis, for example, and genetically, as reflected in gene expression orchestration. These rearrangements include the activation of some TEs and the deactivation of others [1, 2, 5, 51]. However, the underlying mechanism of genomic reorganization involving TEs remains poorly understood. Nonetheless, MITEs are considered as one of the most abundant and successful plant TE groups [18, 52–54].

In this study, we retrieved and analyzed 239,126 MITE insertions belonging to 36 different families from four *Triticum* and *Aegilops* species, including 3,874 members of a newly identified MITE family termed *Inbar*. Our efforts represent the most updated and detailed analysis of MITE composition in wheat genomes, including analysis of MITE distribution in the seven homologous chromosomes of the tetraploid and hexaploid wheat species, available to date. For comparison, in a previous work [30], we reported the analysis of ~18,000 *Stowaway*-like elements retrieved from the shotgun sequence draft of a 454-pyrosequence of *T*.*aestivum* [31]. In the present study, 15,420 insertions were detected in the *T*. *urartu* genome, 30,366 insertions were found in the *Ae*. *tauschii* genome, 78,102 insertions were noted in the *T*. *turgidum* ssp. *dicoccoides* genome, and 115,169 insertions were identified in the *T*. *aestivum* genome. For some MITE families, the hexaploid copy number is similar to the additive value of the parent copy numbers (*T*. *turgidum* ssp. *dicoccoides* + *Ae*. *tauschii*). However, as MITEs are class II elements, transposing using a “cut and paste” mechanism, transposition does not always result in increased copy number. Moreover, as we reported before [43, 47], MITEs activation following polyploidization is not necessarily followed by an increase in copy number. Analysis of common insertions between the A or B sub-genome of hexaploid and tetraploid wheats showed that around 30–47% of the insertions are common (meaning, they were inherited from the tetraploid to the hexaploid), while the rest are unique to either tetraploid or hexaploid wheat. These unique insertions might be the result of a species-specific activity in the tetraploid, transposition of MITEs following speciation of hexaploid wheat (hexaploidization) or due to different genomic rearrangements, such as deletion of MITE-containing sequences.

*Stowaway* is the most abundant MITE superfamily in the wheat genome, representing ~80% of insertions, much as was previously reported for other plant species [18, 55]. The largest number of MITEs is found in *T*. *aestivum* (ABD) with 113,258 known MITE insertions and 1,911 *Inbar* insertions, yielding a total of 115,169 MITE elements. This value is more than the number of MITEs counting during sequencing of the *T*. *aestivum* genome (102,275 insertions [7]).

All MITE families found in the polyploid genomes are also found in the diploid genomes, except for the *Murray* family that presents a single copy in wild emmer wheat and two copies in bread wheat, yet none in the diploid species (Table 1). The *Thalos*, *Athos* and *Pan* families (*Stowaway* superfamily) have the highest copy numbers in all four species, together representing around 60–70% of all MITEs in each genome. As such, it is likely that these were the t the most active MITEs during wheat evolution. Indeed, our findings confirm a previous report of *Thalos* being the most abundant MITE family in wheat [9].

MITE insertions are distributed across the seven homologous chromosomes of the tetraploid and hexaploid species, with the B sub-genome of wild emmer wheat containing 58.4% of MITE insertions and the B sub-genome of *T*. *aestivum* containing 40.4% of the MITE insertions (Fig 3), reflected the previously described highly repetitive nature of the B sub-genome of polyploid wheat [2]. While the B sub-genome, comprising 6,274 Mbp, is the largest sub-genome of *T*. *aestivum* and thus correlating with a larger number of MITEs, the 4,937 Mbp-long D sub-genome is the smallest but contains more MITE elements than does tje A sub-genome (5,727 Mbp [9]). This indicates that different proliferation levels of MITEs exist in the different sub-genomes.

We noticed that *Stowaway* MITEs show a preference for insertion into dinucleotide TA target sites, while *Tourist* MITEs prefer non-specific target sites of 2–3 nucleotides and *Mutator* MITEs shows no preference in 9–10 nucleotide-long target sites where these are found (see S1 Text), in agreement with previous reports [10, 18, 55–57]. In addition, we showed that most MITE insertions preferably insert into genic or repetitive regions, with ~50% of insertions being found within or in close proximity to protein-coding genes, and 40% of insertions being found within both class I and II TEs. The strong association of MITEs with plant genes was reported in several studies [10, 21–24], although the presence of MITEs within other TEs was not reported previously. We found many insertions of different MITE families in class II elements, mostly in the currently non-autonomous *Jorge* family, a large derivative of the *CACTA* superfamily. In addition, we characterized a new MITE family named *Inbar* that is found, in most cases, within the retrotransposon sequences of members of the *Inga* and *Eugene* families (*Copia* superfamily). One possibility for the insertion of *Inbar* into retrotransposons is that during genomic rearrangements following allopolyploidization, transposable elements that are being transcribed become target sites for MITEs that usually insert into genes by an unknown mechanism. It is possible this is an alternative mechanism for MITE proliferation. Upon inserting into an active TE, the MITE element would copy itself, together with the active element (in the case of class I elements) or move with the active element (in the case of class II elements) to a different location in the genome. Alternatively, this could be a host defence mechanism whereby MITEs insert into the coding regions of active TEs, thus causing mutations and leading to de-activation of once-active TEs.

To further examine the association of MITEs with genes, we retrieved MITE elements from the *T*. *aestivum* transcriptome and found ~480 MITE-containing transcripts. Most insertions were located to the 3’ UTR of genes and only a few were found in coding sequences. In addition, in almost all cases of a gene with alternative splice variants, the MITE-containing transcript was longer than the other transcripts, even though the protein was not necessarily longer. This shows that MITEs are found not only within introns but also in the non-coding regions of genes, thus possibly playing a role in their regulation, as was reported [26–29]. In one case, insertion of a *Tourist* MITE insertion into the 3’UTR of a bread wheat heat shock protein gene (*TaHSP16*.*9-3A*) led to increased levels of the gene transcript [58]. In another case, MITE insertion into a regulatory element of *ZmRap2*.*7*, a flowering repressor, was found to affect the expression of this gene by affecting the insertion methylation status [59]. MITE insertions can also lead to allelic variation of plant genes, as we recently showed in emmer wheat [60]. It was previously reported that more than ~3,500 MITEs are transcribed with rice genes [53]. If so, our numbers may be underestimated, especially given how there are possibly other, as yet unrecognized MITE families.

In summary, our high-resolution analysis of MITEs in diploid and polyploid genome drafts sheds light on the proliferation of MITEs during genomic rearrangements, as well as insertion mechanisms, and on the role of TEs in shaping the wheat genome by creating allelic diversity. The high dynamics of TEs in polyploidy species might facilitate the rapid adaptation of newly emerged allopolyploid species.

## Supporting information

S1 Text Analysis of sequence conservation and target site preference. (DOCX)

S1 Table Plant accessions used in site-specific PCR analyses. (DOCX)

S2 Table Copy Number of MITEs insertions in *Triticum* and *Aegilops* species by sub-genome. (DOCX)

S3 Table Characterization of MITE flanking sequences in wild emmer and bread wheats. (XLSX)

S4 Table Characterization of MITE-containing transcripts in the *T*. *aestivum* transcriptome. (XLSX)

S5 Table Characterization of *Inbar* flanking sequences. (XLSX)

S6 Table Primer sequences for *Inbar* insertions in wheat genes. (DOCX)

S1 Fig Distribution of MITE families in the seven homologous chromosomes of *T*. *turgidum* ssp *dicoccoides* (AB). (PDF)

S2 Fig Distribution of MITE families in the seven homologous chromosomes of *T*. *aestivum* (ABD). (PDF)

S3 Fig Copy number of all MITE families in *Triticum* and *Aegilops* genomes. (PDF)
