# Supplementary material for: Genome-wide analyses of miniature inverted-repeat transposable elements reveals new insights into the evolution of the Triticum-Aegilops group
Source: PLoS One. 2018 Oct 24;13(10):e0204972. doi: 10.1371/journal.pone.0204972 (PMC6200218; doi:10.1371/journal.pone.0204972)
Supplement: S1 Text — (DOCX) [file pone.0204972.s001.docx]

**S1 Text. Analysis of sequence conservation and target site preference.**

**Sequence conservation.**

Using multiple sequence alignment and sequence polymorphism analysis (see "Materials and Methods”), we have observed a high sequence conservation for most MITE families in *Triticum* and *Aegilops* species (Table S1 text). The sequence similarity levels ranged from 22.2% to 100% in *T. aestivum*, 27.9% to 100% in *T. turgidum* ssp*. diccocoides*, 25% to 100% in *Ae. tauschii* and 37.17% to 100% in *T. urartu*. Sequence conservation levels are indicators of age and activity level, as a TE family with high sequence conservation is considered relatively young and recently active, while a TE family with low sequence conservation is considered "old" and inactive (fossil elements) as it has accumulated mutations [[1](#_ENREF_1)].

This analysis showed that most of MITE families (20 of the 35 families) are conserved as their sequence similarity levels ranged around 70-100% in all four species (Table S1 text). Families that presented high sequence similarities in all four species (~90% and more) were *Aison*, *Icarus, Xados, Minos, Belus, Stolos, Oleus, Antonio, Kerberos, Xenon, Gerald, Rhea* and *Keres,* indicating that those families are also relatively young and recently active (in evolutionary time). Conservation regions were found (using DNAsp software, see “Materials and Methods”) mostly at tandem inverted repeats (TIR) regions, such as 1-31 bp from the sequences of *Aison*, *Icarus*, *Stolos* and *Xenon*. It is important to mention that we have used an e-value of 1e-3 to retrieve sequences using MAK software, thus this analysis was unbiased toward highly conserved elements.

Some MITE families showed huge variation in sequence conservation among the four species. The different conservation levels for the same family in different *Triticum* and *Aegilops* species might indicate the different proliferation levels in these genomes. This phenomenon is very clear for *Athos*, *Pan*, and *Hades* families. *Athos* family showed 83.18% similarity level in *T. aestivum*, 68.6% in *T. turgidum* ssp*. diccocoides*, 83.17% in *Ae. tauschii* and 37.17% in *T. urartu*. This indicates that *Athos* elements were possibly active recently in *Ae. tauschii* and *T. aestivum,* perhaps in DD genome and they are inactive in *T. urartu* genome. *Pan* family presented a similarity level of 97.82% in *T. aestivum*, 87.1% in *T. turgidum* ssp*. diccocoides,* 58.8% in *Ae. tauschii* genome and 80.6% in *T. urartu* genome. This indicates *Pan* elements were possibly active recently in the polyploids, and are “old” and inactive in *Ae. tauschii* genome. *Hades* presented 78.9% similarity level in *T. aestivum*, 69.2% in *T. turgidum* ssp*. diccocoides*, 40.1% in *Ae. tauschii* and 92.6% in *T. urartu,* indicating a higher proliferation level in *T. urartu* and possibly also in *T. aestivum* genome, while they are inactive in *Ae. tauschii* genome.

**Target site preference.**

Superfamilies are characterized by their transposase sequences and by a similar length of target site duplications (TSDs) [[2](#_ENREF_2)]). Therefore, we have also analyzed the target site preference of different MITE families in all species examined, based on MAK data output of short duplicated target site sequences from the analysis of both flanking sequences of a MITE element (Table S1 text). We have used WebLogo 3.0 package [[3](#_ENREF_3)] and the MAK data output to calculate the relative frequency of nucleotides at certain positions and create a "logo" to represent the target site preference for each MITE family (Figure S1 text).

Most *Stowaway* MITE families (*Aison*, *Antonio*, *Athos*, *Hades*, *Icarus*, *Jason*, *Minimus*, *Minos*, *Marius*, *Oleus*, *Pan*, *Stolos* and *Thalos*) presented a target site preference of the dinucleotide TA (as expected, [[4](#_ENREF_4)]) and the rest (*Tantalos, Fortuna, Xados, Polyphemus*, *Eos* and *Phoebus*) presented a varying target site preference of CC, KT, TC, TH, MK and CG respectively (W= A/T base , Y = C/T base, M = A/C base, K = G/T base, H=A/C/T base). It has been reported that *Stowaway* MITEs have a target site preference of TA [[4-6](#_ENREF_4)] and we also found this to be true for most *Stowaway* MITEs, however 6 families presented a different target site preference. This approves *Stowaway* MITEs do usually prefer a dinucleotide target site, although not necessarily TA.

All *Tourist* MITE families presented a short target site preference of 2-3 nucleotides and each family presented a unique target site preference that sometimes varied between the *Triticum* and *Aegilops* species. It has been reported that *Tourist* MITEs prefer TNA as a target site, while N refers to A, T, C or G [[2](#_ENREF_2),[4](#_ENREF_4),[7](#_ENREF_7)], however we have found only two *Tourist* MITE families, *Orpheus* and *Coeus*, that presented a THA target site preference clearly. The other families presented a preference for TA, AG and GYA (*Kerberos*, *Xenon* and *Victor*, respectively). This indicates the previously reported TNA target site preference might not be absolute for all *Tourist* MITEs but rather for some *Tourist* families. Most *Mutator* MITE families (*Argus*, *Gerald*, *Spring,* *Rhea* and *Murray*) presented a long varying target site preference of 9-10 nucleotides, usually with a conserved beginning or end, and the rest (*Vacuna*, *Remus* and *Gabriel*) presented a short varying target site preference of 2-4 nucleotides. It was previously reported that *Mutator* MITEs have a target site preference of 9-11 nucleotides [[4](#_ENREF_4),[8](#_ENREF_8)] and indeed most *Mutator* MITEs families we have found presented this characteristic with no unique sequence for all families.

Each of the unknown superfamilies MITE family (*Belus*, *Gorgon* and *Keres*) presented target site preferences of varying lengths (2-7 nucleotides) - CATG, GC and CGGTCCG respectively. Overall, most of the MITE elements were inserted into sites containing mostly of AT dinucleotides, which makes sense as CG sites are more prone to methylations.

| **Table S1 text.** Target site preferences and sequence conservation levels of MITE families. | | | | | | | | | | | |
| --- | --- | --- | --- | --- | --- | --- | --- | --- | --- | --- | --- |
| **Group** | **Superfamily** | **Family** | **Target Site Preference (TSD)** | | | | **Conserved TSD motifs** | **Sequence Similarity (%)** | | | |
|  |  |  | ***T. aestivum*** | ***T. turgidum*** | ***Ae. tauschii*** | ***T. urartu*** |  | ***T. aestivum*** | ***T. turgidum*** | ***Ae. tauschii*** | ***T. urartu*** |
| MITE | *Stowaway* | *Thalos* | TA | TA | TA | TA | TA | 81.78 | 74.4 | 76.42 | 89.96 |
| MITE | *Stowaway* | *Athos* | TA | TA | TA | TA | TA | 83.18 | 68.6 | 83.17 | 37.17 |
| MITE | *Stowaway* | *Pan* | TA | TA | TA | TA | TA | 97.82 | 87.1 | 58.80 | 80.60 |
| MITE | *Stowaway* | *Icarus* | TA | TA | TA | TA | TA | 89.87 | 89.5 | 98.30 | 97.70 |
| MITE | *Stowaway* | *Hades* | TA | TA | TA | TA | TA | 78.90 | 69.2 | 40.10 | 92.60 |
| MITE | *Stowaway* | *Xados* | TC | TC | TC | TC | TC | 96.80 | 99.7 | 99.20 | 98.80 |
| MITE | *Stowaway* | *Eos* | AG | CT | AG | AG | MK | 96.70 | 84.8 | 98.70 | 99.70 |
| MITE | *Stowaway* | *Minos* | TA | TA | TA | TA | TA | 99.40 | 99.0 | 98.40 | 99.80 |
| MITE | *Stowaway* | *Aison* | TA | TA | TA | TA | TA | 98.20 | 98.8 | 96.00 | 97.20 |
| MITE | *Stowaway* | *Stolos* | TA | TA | TA | TA | TA | 98.80 | 97.8 | 98.80 | 98.70 |
| MITE | *Stowaway* | *Oleus* | TA | TA | TA | TA | TA | 97.40 | 92.2 | 97.10 | 94.20 |
| MITE | *Stowaway* | *Antonio* | TA | TA | TA | TA | TA | 94.60 | 91.1 | 92.90 | 89.90 |
| MITE | *Stowaway* | *Minimus* | TA | TA | TA | TA | TA | 51.00 | 59.9 | 55.50 | 74.10 |
| MITE | *Stowaway* | *Fortuna* | TA | GT | TA | TA | TW | 91.30 | 97.7 | 78.10 | 94.40 |
| MITE | *Stowaway* | *Tantalos* | CC | CC | CC | CC | YC | 75.40 | 74.5 | 72.70 | 59.50 |
| MITE | *Stowaway* | *Phoebus* | CG | CG | CG | CG | CG | 22.20 | 27.9 | 25.00 | 100.00 |
| MITE | *Stowaway* | *Polyphemus* | TC | TC | AC | CTA | TC | 34.50 | 100.0 | 50.00 | - |
| MITE | *Stowaway* | *Jason* | TA | GA | TA | TA | TA | 25.00 | 100.0 | 52.40 | 100.00 |
| MITE | *Tourist* | *Orpheus* | TAA | TAA | TAA | TCA | THA | 78.90 | 93.4 | 98.80 | 99.70 |
| MITE | *Tourist* | *Kerberos* | TA | TA | AA | TA | TA | 99.00 | 99.0 | 99.20 | 92.90 |
| MITE | *Tourist* | *Coeus* | TTA | TCA | TAA | TTA | THA | 97.30 | 97.3 | 82.40 | 96.90 |
| MITE | *Tourist* | *Xenon* | AG | AG | AG | AG | AG | 98.20 | 97.0 | 97.50 | 98.30 |
| MITE | *Tourist* | *Victor* | GCA | GCA | GTA | GCA | GYA | 78.80 | 68.9 | 88.90 | 84.50 |
| MITE | *Mutator* | *Gerald* | AAAAATTAA | AAAAAAAAA | AAAAATAAA | AAAAAAAAA | WWWWWWWWA | 99.10 | 96.1 | 98.50 | 99.50 |
| MITE | *Mutator* | *Rhea* | TACAAAAAA | TACAAAAAA | TACAAAAAA | TATATTAAA | TAHAAAAAA | 97.00 | 95.8 | 96.20 | 98.00 |
| MITE | *Mutator* | *Spring* | GGGGAACC | GGGACTATC | GGATTTTAG | CACGATCCC | GGGGAATAC | 94.30 | 95.0 | 79.40 | 42.60 |
| MITE | *Mutator* | *Argus* | TTTAATTAA | TATAATAAA | TTTATTCTA | TTAAAATAA | WWWWWWWWA | 94.30 | 96.1 | 28.60 | 100.00 |
| MITE | *Mutator* | *Vacuna* | TTT | TG | GG | GG | GGT | 87.60 | 89.0 | 70.40 | 73.10 |
| MITE | *Mutator* | *Gabriel* | CCTC | CT | CCTC | CC | CY | 33.30 | 50.5 | 100.00 | 100.00 |
| MITE | *unknown* | *Belus* | CATG | CATG | CATG | CATG | CATG | 99.78 | 99.3 | 94.80 | 92.20 |
| MITE | *unknown* | *Keres* | CGGTCCG | CGGTCCG | CGGTCCG | CGGTCCG | CGGTCCG | 92.30 | 95.1 | 89.50 | 92.20 |
| MITE | *unknown* | *Gorgon* | GC | GC | CC | GT | GC | 87.30 | 74.7 | 93.00 | 79.80 |
| unknown^1^ | *Mutator* | *Remus* | CG | CG | CG | CC | CG | 81.60 | 93.4 | 100.00 | 65.80 |
| unknown^1^ | *Stowaway* | *Marius* | TA | TA | TA | TA | TA | 100.00 | 100.0 | 66.70 | 100.00 |
| unknown^1^ | *Mutator* | *Murray* | TACTGCTCC | TA | - | - | TACTGCTCC | - | - | - | - |

^1^Short TIR families with no classification to MITE or LITE

**a.**


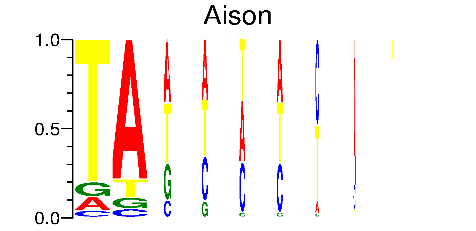

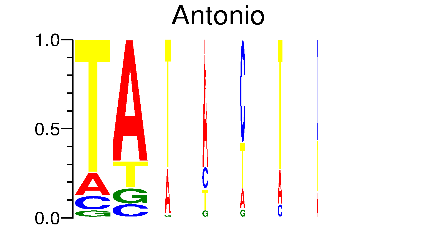

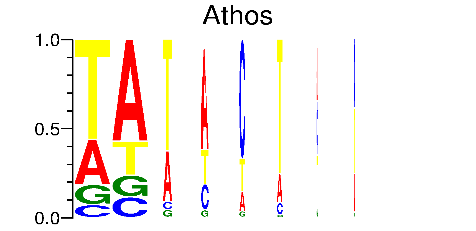

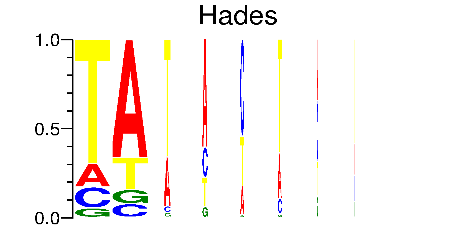

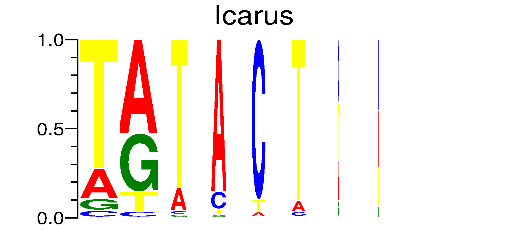

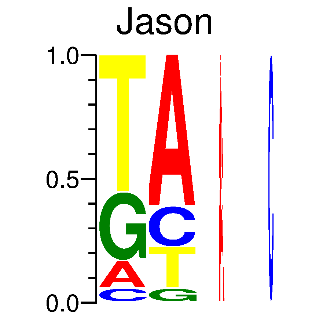

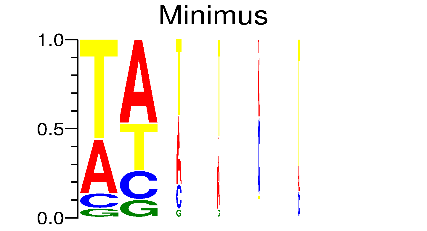

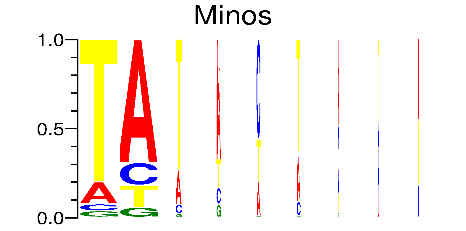

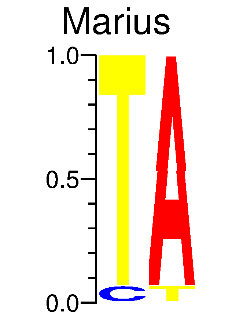

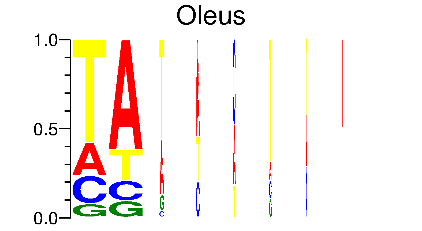

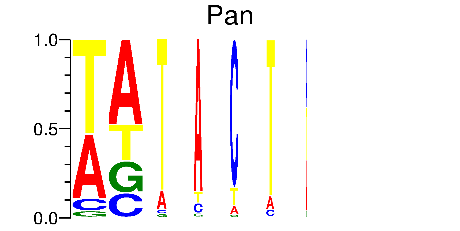

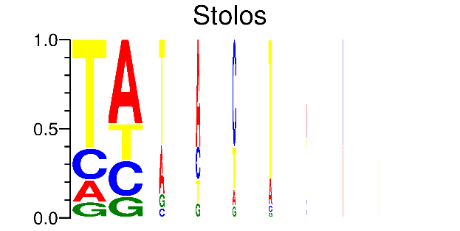

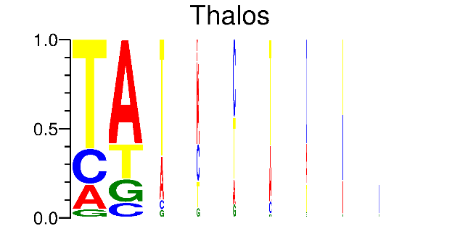

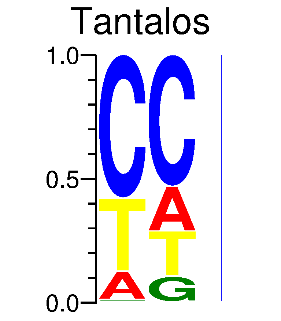

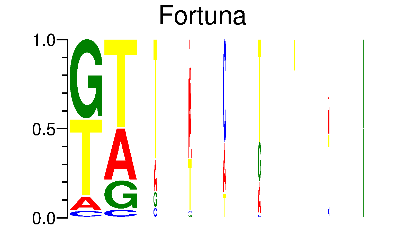

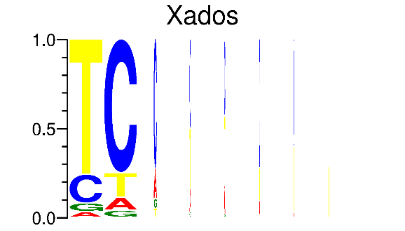

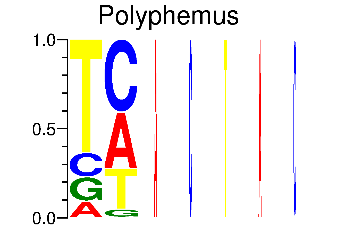

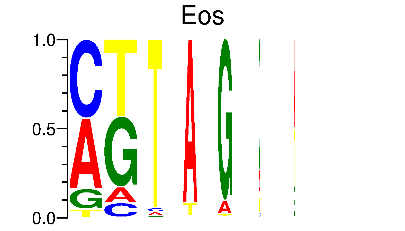

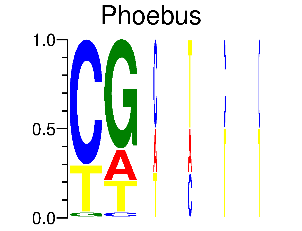


**b.**


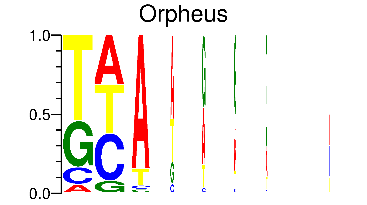

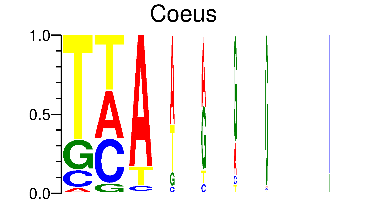

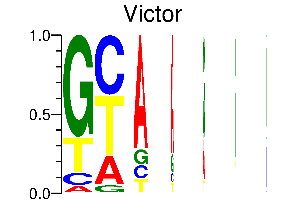

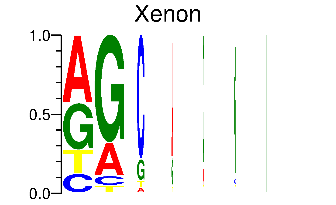

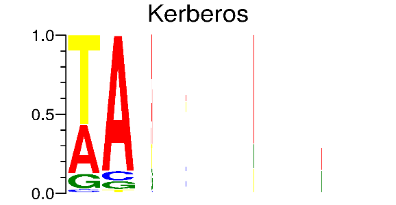


**c.**


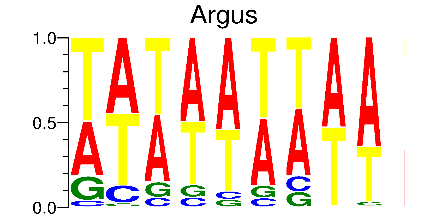

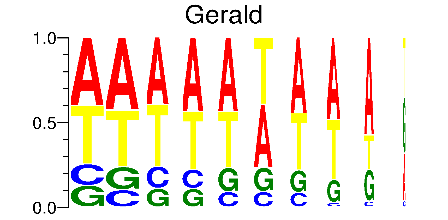

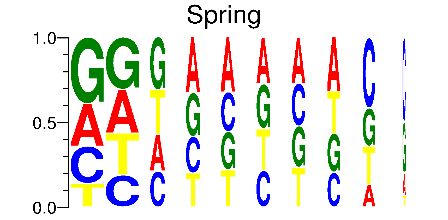

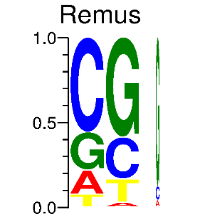

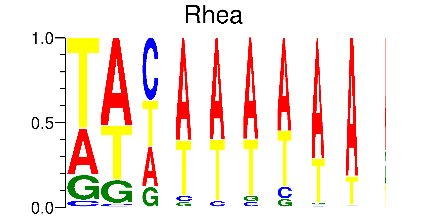

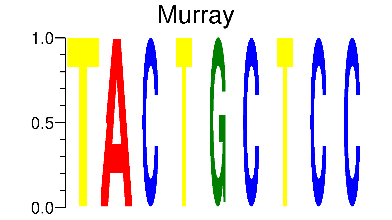

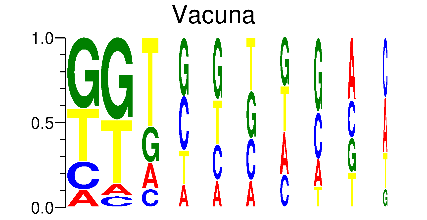

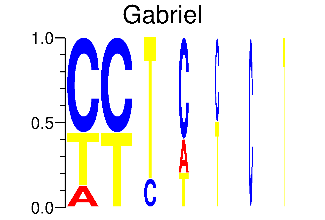


**d.**


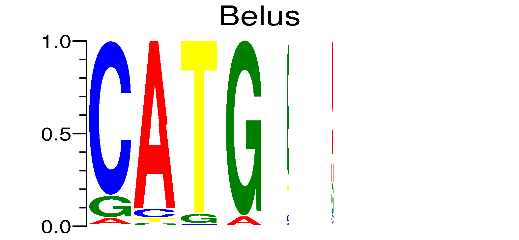

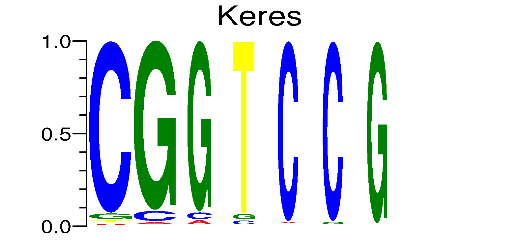

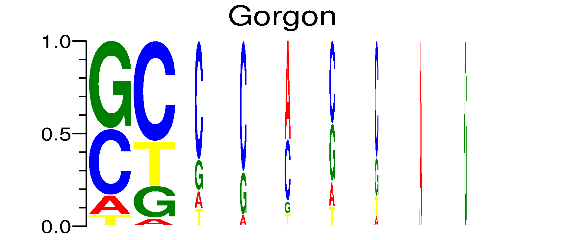


**Figure S1 text. Target site preferences of MITE families** created using WebLogo 3.0 package, based on MAK data output of target site duplications from *T. aestivum*. **a**. Target site preference of *Stowaway* MITE families. Most *Stowaway* MITE families (*Aison, Antonio, Athos, Hades, Icarus, Jason, Minimus, Minos, Marius, Oleus, Pan, Stolos* and *Thalos*) presented a target site preference of the dinucleotide TA, while the other *Stowaway* families (*Tantalos, Fortuna, Xados, Polyphemus, Eos* and *Phoebus*) presented a varying target site preference of CC, KT, TC, TH, MK and CG respectively (W= A/T base, Y = C/T base, M = A/C base, K = G/T base). **b**. Target site preference of *Tourist* MITE families. Two *Tourist* MITE families, *Orpheus* and *Coeus* presented a THA target site preference (H=A/C/T base). The other families presented a preference for GY, AG and TA (*Victor*, *Xenon* and *Kerberos*, respectively). **c**. Target site preference of *Mutator* MITE families. Most *Mutator* MITE families (*Argus, Gerald, Spring*, *Rhea*, *Murray* and *Vacuna*) presented a long varying target site preference of 9-10 nucleotides, usually with a conserved beginning or end, and the rest (*Remus and Gabriel*) presented a short varying target site preference of 2-4 nucleotides (CG and CYT, respectively). **d**. Target site preference of MITE families belonging to unknown superfamilies. Each family (*Belus*, *Keres* and *Gorgon*) presented a unique target site preference in varying lengths (2-7 nucleotides) - CATG, CGGTCCG and GC respectively.

1. Lenoir A, Lavie L, Prieto J-L, Goubely C, Cote J-C, et al. (2001) The evolutionary origin and genomic organization of SINEs in Arabidopsis thaliana. Molecular biology and evolution 18: 2315-2322.

2. Zhao D, Ferguson AA, Jiang N (2016) What makes up plant genomes: The vanishing line between transposable elements and genes. Biochimica et Biophysica Acta (BBA)-Gene Regulatory Mechanisms 1859: 366-380.

3. Crooks GE, Hon G, Chandonia J-M, Brenner SE (2004) WebLogo: a sequence logo generator. Genome research 14: 1188-1190.

4. Feschotte C, Zhang X, Wessler SR (2002) Miniature inverted-repeat transposable elements (MITEs) and their relationship with established DNA transposons. Mobile DNA II: 1147-1158.

5. Feschotte C, Swamy L, Wessler SR (2003) Genome-wide analysis of mariner-like transposable elements in rice reveals complex relationships with stowaway miniature inverted repeat transposable elements (MITEs). Genetics 163: 747-758.

6. Yang G, Nagel DH, Feschotte C, Hancock CN, Wessler SR (2009) Tuned for transposition: molecular determinants underlying the hyperactivity of a Stowaway MITE. science 325: 1391-1394.

7. Jiang N, Feschotte C, Zhang X, Wessler SR (2004) Using rice to understand the origin and amplification of miniature inverted repeat transposable elements (MITEs). Current opinion in plant biology 7: 115-119.

8. Wicker T, Sabot F, Hua-Van A, Bennetzen JL, Capy P, et al. (2007) A unified classification system for eukaryotic transposable elements. Nature Reviews Genetics 8: 973-982.
