# Supplementary material for: Genome-wide analyses of miniature inverted-repeat transposable elements reveals new insights into the evolution of the Triticum-Aegilops group
Source: PLoS One. 2018 Oct 24;13(10):e0204972. doi: 10.1371/journal.pone.0204972 (PMC6200218; doi:10.1371/journal.pone.0204972)
Supplement: S1 Table — (DOCX) [file pone.0204972.s002.docx]

**S1 Table: Wheat species and accessions used in this study.**

| **Species (genome composition)** | **Accession number** | **Origin** |
| --- | --- | --- |
| *Aegilops sharonensis* (BB) | TH02 | Israel |
| *Aegilops sharonensis* (BB) | TH01 | Israel |
| *Aegilops longissima* (BB) | TL10 | Jordan |
| *Aegilops searsii* (BB) | TE44 | Jordan |
| *Aegilops searsii* (BB) | 599140 | Jordan |
| *Aegilops searsii* (BB) | TE16 | Syria |
| *Aegilops speltoides* (BB) | TS118 | Syria |
| *Aegilops speltoides* (BB) | TS02 | Israel |
| *Aegilops speltoides* (BB) | 542274 | Turkey |
| *Triticum urartu* (AA) | TMU38 | Israel |
| *Triticum urartu* (AA) | TMU06 | Israel |
| *Triticum monococcum* ssp. *aegilopoides* (AA) | TMB02 | Israel |
| *Aegilops tauschii* (DD) | 574468 | Armenia |
| *Aegilops tauschii* (DD) | 603236 | Turkmenistan |
| *Aegilops tauschii* (DD) | TQ27 | Israel |
| *Triticum turgidum* ssp. *dicoccoides* (AABB) | Wild Emmer | Israel |
| *Triticum turgidum* ssp. *dicoccoides* (AABB) | TTD48 | Iran |
| *Triticum turgidum* ssp. *dicoccoides* (AABB) | TTD20 | Israel |
| *Triticum turgidum* ssp. *durum* (AABB) | TTR19 | Israel |
| *Triticum turgidum* ssp. *durum* (AABB) | Svevo | Italy |
| *Triticum turgidum* ssp. *durum* (AABB) | TTR16 | Israel |
| *Triticum aestivum* (AABBDD) | 377626 | Former Yugoslavia |
| *Triticum aestivum* (AABBDD) | 78809 | Georgia |
| *Triticum aestivum* (AABBDD) | 574497 | Canada |
| *Triticum aestivum* (AABBDD) | TTR19 x TQ27  Synthetic allohexaploids | Israel |
