# Supplementary material for: Genome-wide analyses of miniature inverted-repeat transposable elements reveals new insights into the evolution of the Triticum-Aegilops group
Source: PLoS One. 2018 Oct 24;13(10):e0204972. doi: 10.1371/journal.pone.0204972 (PMC6200218; doi:10.1371/journal.pone.0204972)
Supplement: S2 Table — (DOCX) [file pone.0204972.s003.docx]

**S2 Table.** **Copy Number of MITEs insertions in *Triticum* and *Aegilops* species by sub-genome (sorted by superfamily and copy number).**

| **Family** | **Group** | **Superfamily** |  | **Copy Number** | | | | | |  |
| --- | --- | --- | --- | --- | --- | --- | --- | --- | --- | --- |
|  |  |  | **A sub-genome** | | | **B sub-genome** | | **D sub-genome** | |  |
|  |  |  | ***T. urartu*** | ***T. turgidum*** | ***T. aestivum*** | ***T. turgidum*** | ***T. aestivum*** | ***Ae. tauschii*** | ***T. aestivum*** |  |
| *Thalos* | MITE | *Stowaway* | 5249 | 9689 | 9699 | 17522 | 17202 | 12557 | 14600 |  |
| *Athos* | MITE | *Stowaway* | 2314 | 5231 | 6430 | 4979 | 6138 | 5297 | 6186 |  |
| *Pan* | MITE | *Stowaway* | 1407 | 3721 | 3668 | 7132 | 7167 | 3838 | 3061 |  |
| *Icarus* | MITE | *Stowaway* | 694 | 1274 | 1365 | 3093 | 3437 | 1649 | 1857 |  |
| *Hades* | MITE | *Stowaway* | 643 | 861 | 848 | 1637 | 1683 | 898 | 1032 |  |
| *Eos* | MITE | *Stowaway* | 465 | 1433 | 1431 | 670 | 647 | 974 | 1140 |  |
| *Xados* | MITE | *Stowaway* | 344 | 455 | 475 | 924 | 918 | 445 | 488 |  |
| *Minos* | MITE | *Stowaway* | 636 | 827 | 871 | 167 | 151 | 164 | 194 |  |
| *Aison* | MITE | *Stowaway* | 166 | 265 | 279 | 400 | 414 | 115 | 142 |  |
| *Stolos* | MITE | *Stowaway* | 232 | 290 | 287 | 201 | 185 | 197 | 220 |  |
| *Fortuna* | MITE | *Stowaway* | 112 | 342 | 351 | 93 | 108 | 35 | 47 |  |
| *Oleus* | MITE | *Stowaway* | 106 | 128 | 142 | 116 | 114 | 130 | 137 |  |
| *Antonio* | MITE | *Stowaway* | 84 | 103 | 107 | 112 | 114 | 84 | 83 |  |
| *Minimus* | MITE | *Stowaway* | 76 | 76 | 75 | 123 | 141 | 86 | 98 |  |
| *Tantalos* | MITE | *Stowaway* | 28 | 27 | 27 | 42 | 38 | 31 | 34 |  |
| *Phoebus* | MITE | *Stowaway* | 3 | 6 | 10 | 20 | 16 | 7 | 8 |  |
| *Polyphemus* | MITE | *Stowaway* | 1 | 2 | 2 | 9 | 12 | 8 | 8 |  |
| *Jason* | MITE | *Stowaway* | 4 | 5 | 4 | 5 | 4 | 7 | 10 |  |
| *Orpheus* | MITE | *Tourist* | 546 | 679 | 713 | 754 | 811 | 299 | 333 |  |
| *Kerberos* | MITE | *Tourist* | 100 | 243 | 244 | 542 | 550 | 689 | 774 |  |
| *Coeus* | MITE | *Tourist* | 163 | 257 | 322 | 333 | 375 | 53 | 55 |  |
| *Xenon* | MITE | *Tourist* | 221 | 244 | 240 | 170 | 160 | 122 | 141 |  |
| *Victor* | MITE | *Tourist* | 45 | 47 | 53 | 55 | 62 | 57 | 78 |  |
| *Gerald* | MITE | *Mutator* | 401 | 525 | 524 | 381 | 353 | 191 | 218 |  |
| *Rhea* | MITE | *Mutator* | 194 | 249 | 251 | 87 | 90 | 124 | 125 |  |
| *Spring* | MITE | *Mutator* | 17 | 53 | 79 | 208 | 194 | 40 | 43 |  |
| *Argus* | MITE | *Mutator* | 10 | 27 | 25 | 250 | 265 | 6 | 9 |  |
| *Vacuna* | MITE | *Mutator* | 40 | 63 | 67 | 70 | 82 | 29 | 36 |  |
| *Gabriel* | MITE | *Mutator* | 7 | 12 | 11 | 2 | 1 | 5 | 4 |  |
| *Belus* | MITE | *unknown* | 929 | 2086 | 2148 | 3986 | 3855 | 2014 | 2421 |  |
| *Keres* | MITE | *unknown* | 100 | 209 | 236 | 251 | 279 | 93 | 92 |  |
| *Gorgon* | MITE | *unknown* | 53 | 56 | 56 | 66 | 76 | 64 | 83 |  |
| *Inbar* | MITE | *unknown* | 93 | 319 | 345 | 1497 | 1510 | 24 | 20 |  |
| *Remus* | unknown | *Mutator* | 27 | 36 | 34 | 169 | 164 | 24 | 7 |  |
| *Marius* | unknown | *Stowaway* | 3 | 4 | 5 | 7 | 7 | 7 | 3 |  |
| *Murray* | unknown | *Mutator* | 0 | 0 | 0 | 1 | 1 | 3 | 0 |  |
| **Sum** |  |  | 15513 | 29844 | 31424 | 46074 | 47324 | 30366 | 33787 |  |
