# Supplementary material for: Genome-wide analyses of miniature inverted-repeat transposable elements reveals new insights into the evolution of the Triticum-Aegilops group
Source: PLoS One. 2018 Oct 24;13(10):e0204972. doi: 10.1371/journal.pone.0204972 (PMC6200218; doi:10.1371/journal.pone.0204972)
Supplement: S6 Table — (DOCX) [file pone.0204972.s007.docx]

**S6 Table. Primer sequences for *Inbar* insertions in wheat genes.**

| **Gene accession *(based on EnsemblPlants)*** | **Gene product (protein)** | **Forward primer** | **Reverse Primer** | **PCR product** |
| --- | --- | --- | --- | --- |
| TRIAE_CS42_2BL_TGACv1_129533_AA0387420 | uncharacterized protein | TGTGGTCTTCGGCCGTAGTA | CTACCGTCCTTAGTACCGCAAA | 101 |
| TRIAE_CS42_6AL_TGACv1_471379_AA1507990 | uncharacterized protein | CTCAAGACGGTGTAGCTTCCTT | GCTAGTACCGTAAAACCCGACA | 512 bp |
| TRIAE_CS42_2AS_TGACv1_113196_AA0352860 | Ribulose bisphosphate carboxylase small chain | GGTACTACCGCTCCTGGAAC | CTCTCCTCTCTTCCTTGTCCTG | 426 bp |
