# Supplementary material for: Genome-wide analyses of miniature inverted-repeat transposable elements reveals new insights into the evolution of the Triticum-Aegilops group
Source: PLoS One. 2018 Oct 24;13(10):e0204972. doi: 10.1371/journal.pone.0204972 (PMC6200218; doi:10.1371/journal.pone.0204972)
Supplement: S1 Fig — (PDF) [file pone.0204972.s008.pdf]

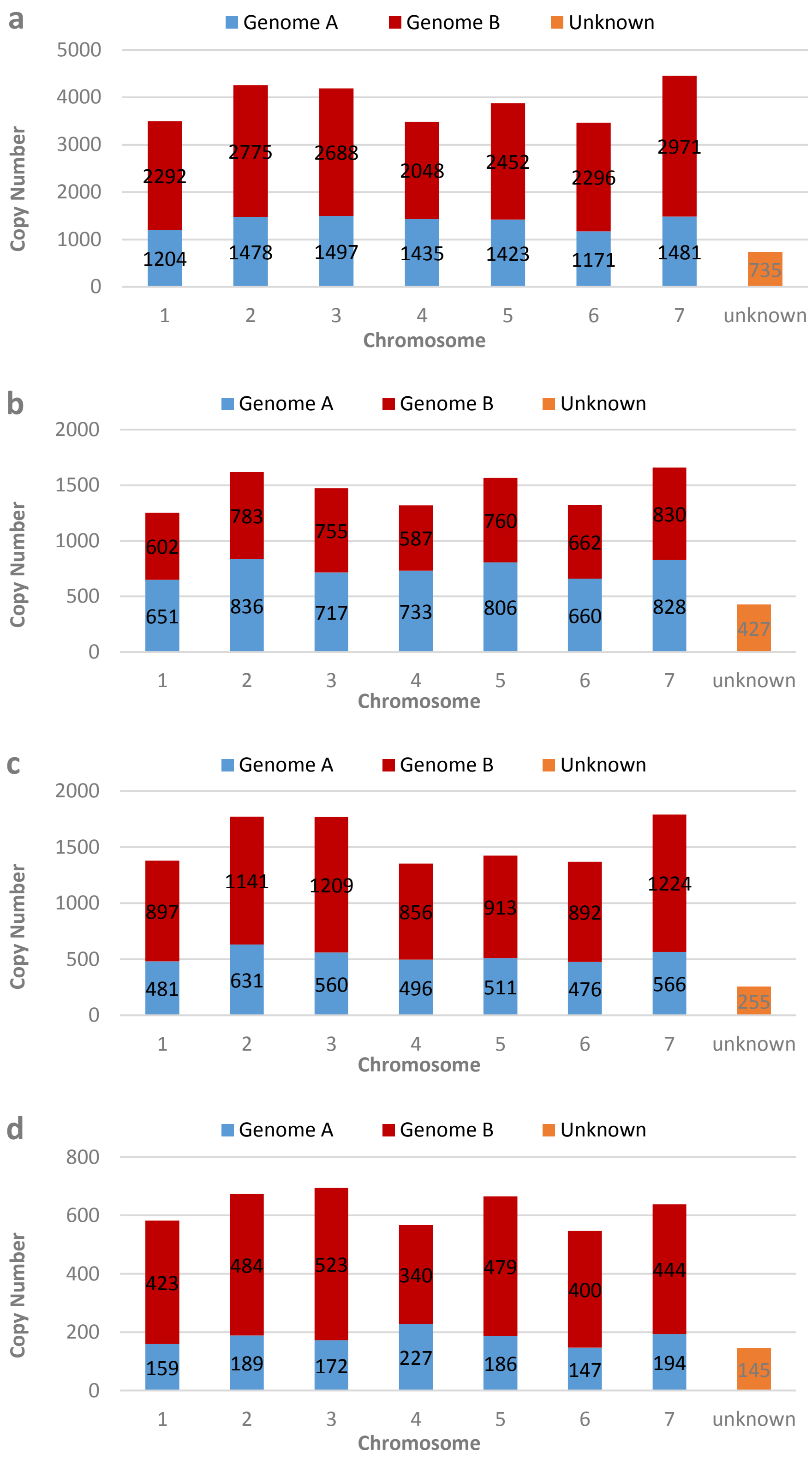

a
Genome A
Genome B
Unknown
5000
4000
3000
2000
1000
0
Copy Number
2292
2775
2688
2048
2452
2296
2971
1204
1478
1497
1435
1423
1171
1481
735
1
2
3
4
5
6
7
unknown
Chromosome
b
Genome A
Genome B
Unknown
2000
1500
1000
500
0
Copy Number
602
783
755
587
760
662
830
651
836
717
733
806
660
828
427
1
2
3
4
5
6
7
unknown
Chromosome
c
Genome A
Genome B
Unknown
2000
1500
1000
500
0
Copy Number
897
1141
1209
856
913
892
1224
481
631
560
496
511
476
566
255
1
2
3
4
5
6
7
unknown
Chromosome
d
Genome A
Genome B
Unknown
800
600
400
200
0
Copy Number
423
484
523
340
479
400
444
159
189
172
227
186
147
194
145
1
2
3
4
5
6
7
unknown
Chromosome

e

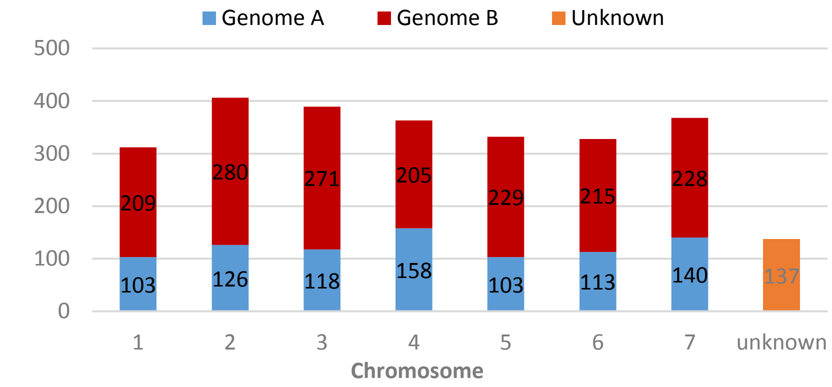
Genome A
Genome B
Unknown
Copy Number
Chromosome
1
2
3
4
5
6
7
unknown
209
280
271
205
229
215
228
103
126
118
158
103
113
140
137

f

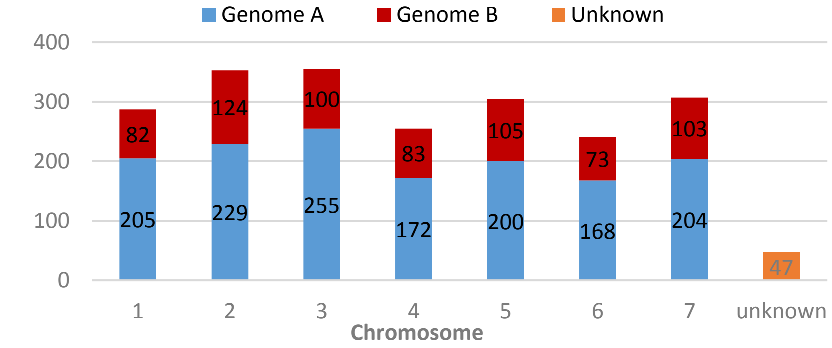
Genome A
Genome B
Unknown
Copy Number
Chromosome
82
124
100
83
105
73
103
205
229
255
172
200
168
204
47

g

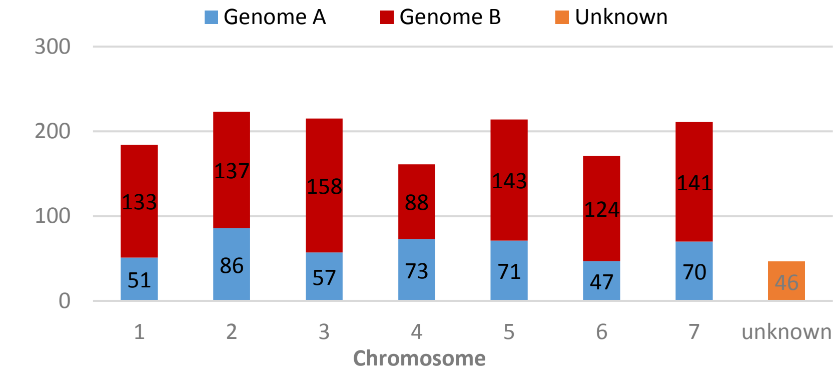
Genome A
Genome B
Unknown
Copy Number
Chromosome
133
137
158
88
143
124
141
51
86
57
73
71
47
70
46

h

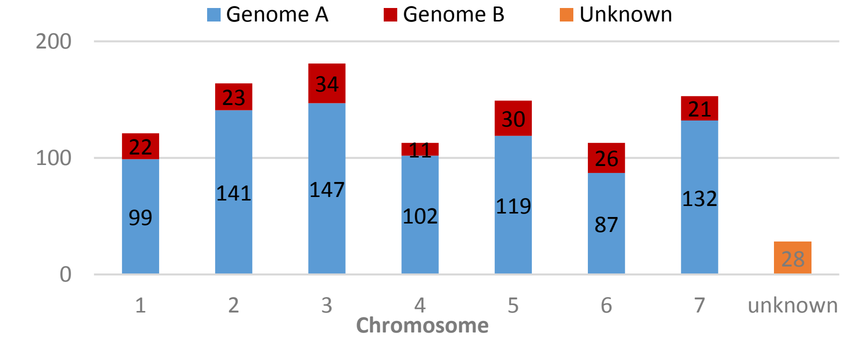
Genome A
Genome B
Unknown
Copy Number
Chromosome
22
23
34
11
30
26
21
99
141
147
102
119
87
132
28

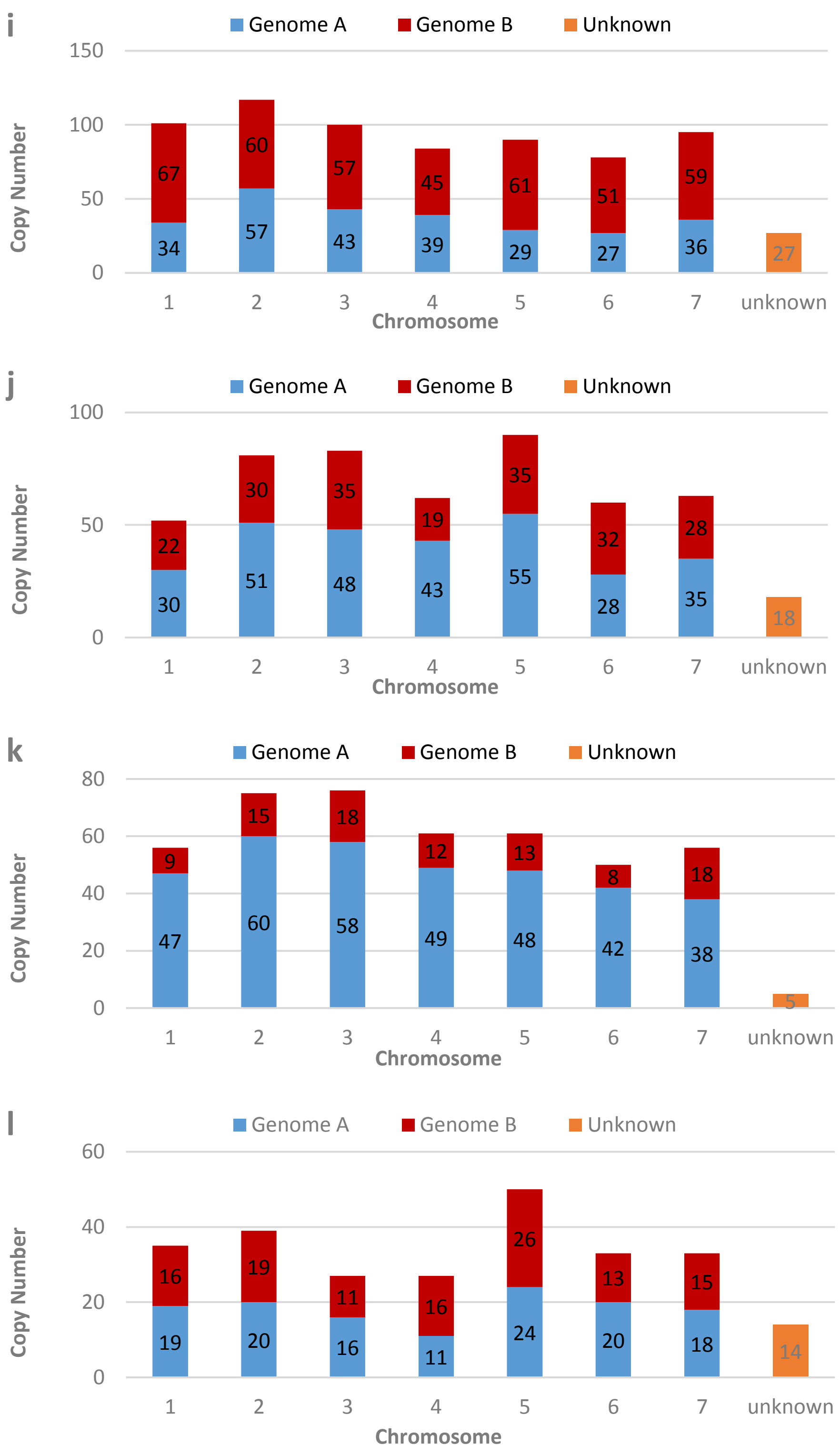
i
Genome A
Genome B
Unknown
150
100
50
0
Copy Number
67
60
57
45
61
51
59
34
57
43
39
29
27
36
27
1
2
3
4
5
6
7
unknown
Chromosome
j
Genome A
Genome B
Unknown
100
50
0
Copy Number
22
30
35
19
35
32
28
30
51
48
43
55
28
35
18
1
2
3
4
5
6
7
unknown
Chromosome
k
Genome A
Genome B
Unknown
80
60
40
20
0
Copy Number
9
15
18
12
13
8
18
47
60
58
49
48
42
38
5
1
2
3
4
5
6
7
unknown
Chromosome
l
Genome A
Genome B
Unknown
60
40
20
0
Copy Number
16
19
11
16
26
13
15
19
20
16
11
24
20
18
14
1
2
3
4
5
6
7
unknown
Chromosome

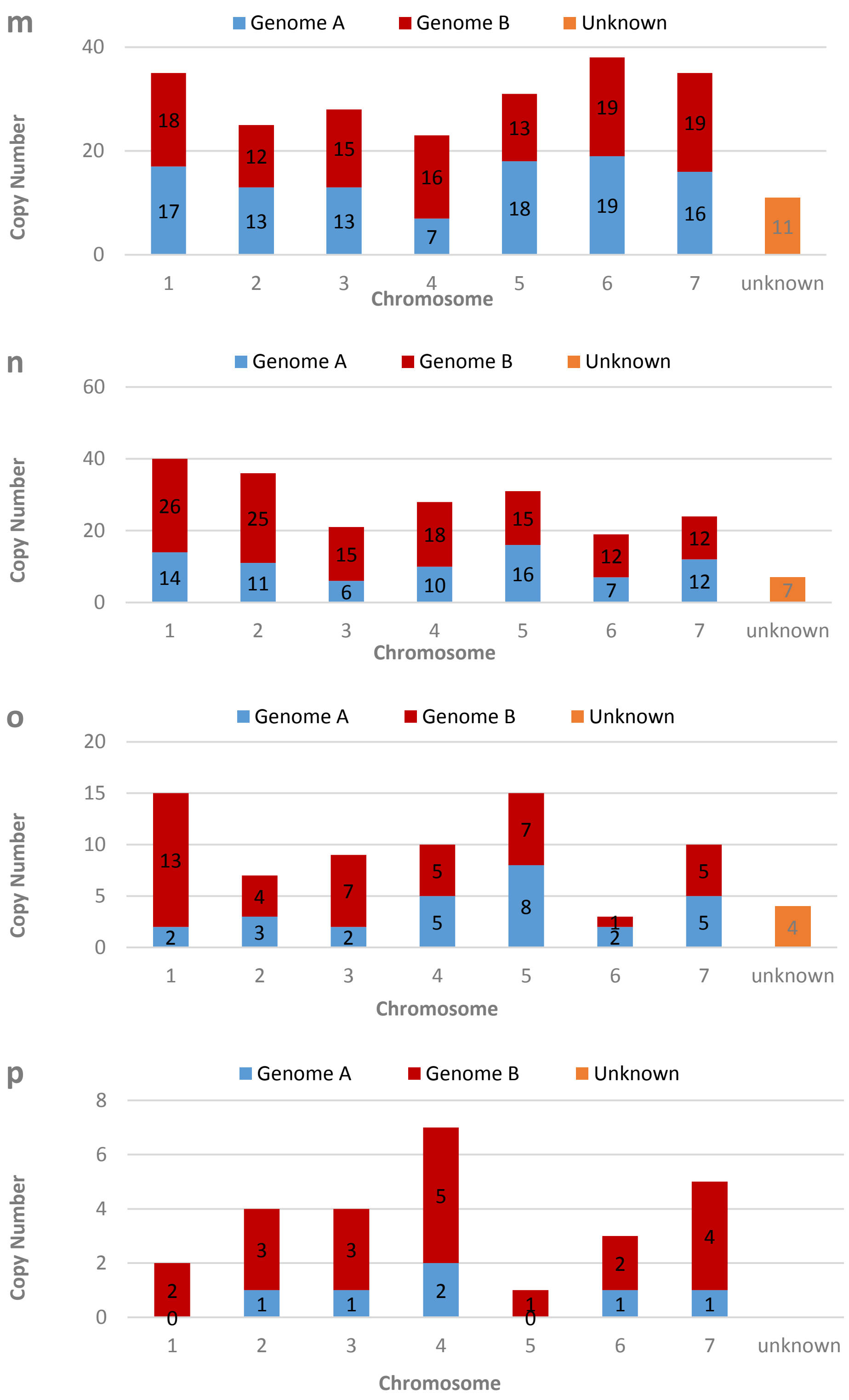
m
Genome A
Genome B
Unknown
Copy Number
40
20
0
18
17
12
13
15
13
16
7
13
18
19
19
19
16
11
1
2
3
4
5
6
7
unknown
Chromosome
n
Genome A
Genome B
Unknown
Copy Number
60
40
20
0
26
14
25
11
15
6
18
10
15
16
12
7
12
12
7
1
2
3
4
5
6
7
unknown
Chromosome
o
Genome A
Genome B
Unknown
Copy Number
20
15
10
5
0
13
2
4
3
7
2
5
5
7
8
1
2
5
5
4
1
2
3
4
5
6
7
unknown
Chromosome
p
Genome A
Genome B
Unknown
Copy Number
8
6
4
2
0
2
0
3
1
3
1
5
2
1
0
2
1
4
1
1
2
3
4
5
6
7
unknown
Chromosome

q

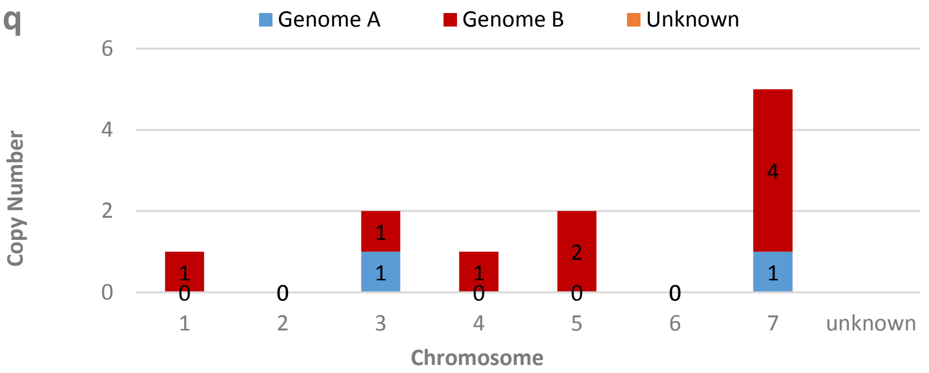
Genome A
Genome B
Unknown
Copy Number
0
2
4
6
1
0
0
1
1
1
0
2
0
0
4
1
1
2
3
4
5
6
7
unknown
Chromosome

r

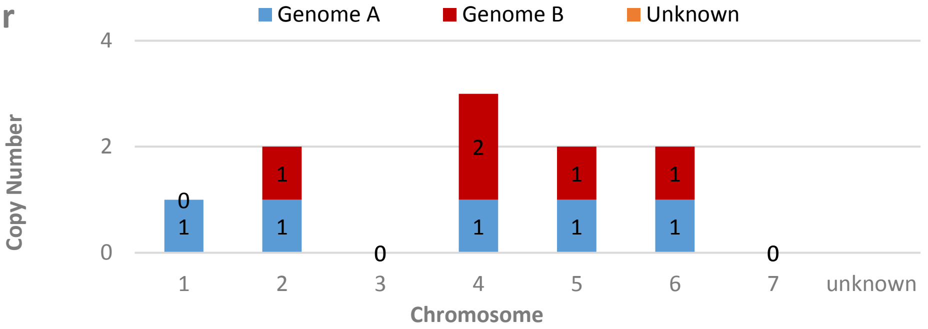
Genome A
Genome B
Unknown
Copy Number
0
2
4
0
1
1
1
0
2
1
1
1
1
1
0
1
2
3
4
5
6
7
unknown
Chromosome

s

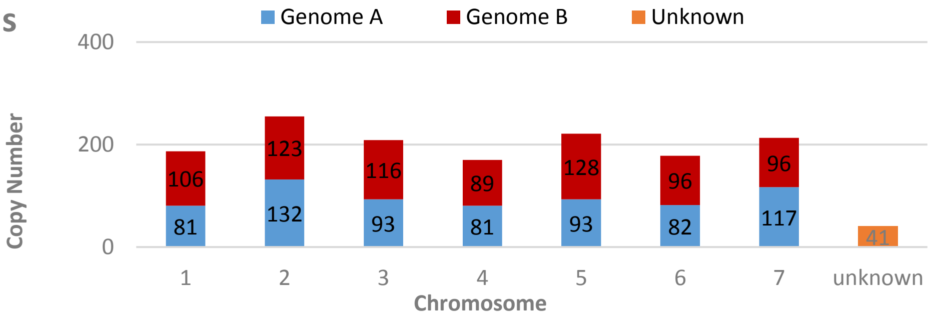
Genome A
Genome B
Unknown
Copy Number
0
200
400
106
81
123
132
116
93
89
81
128
93
96
82
96
117
41
1
2
3
4
5
6
7
unknown
Chromosome

t

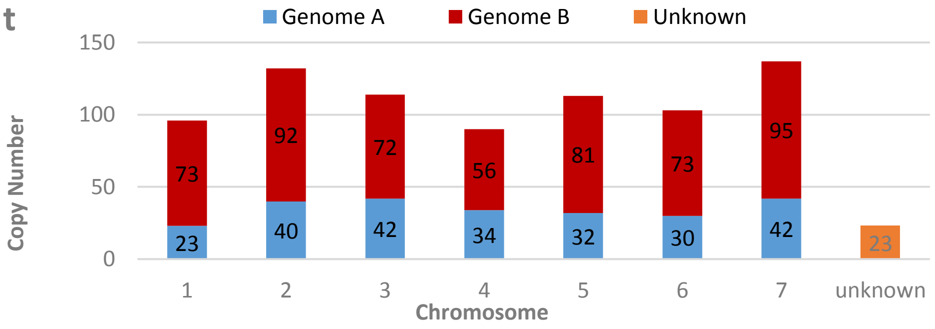
Genome A
Genome B
Unknown
Copy Number
0
50
100
150
73
23
92
40
72
42
56
34
81
32
73
30
95
42
23
1
2
3
4
5
6
7
unknown
Chromosome

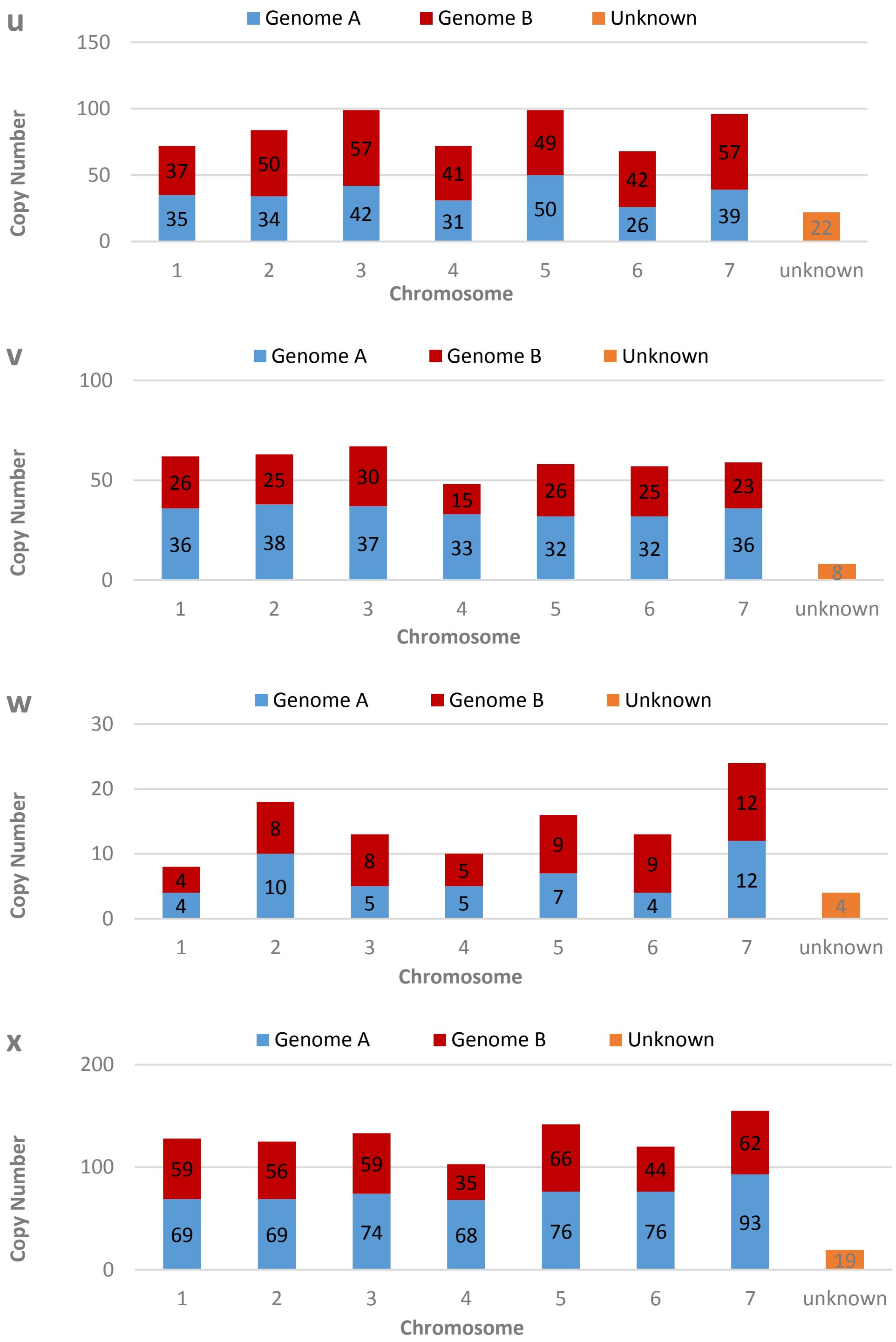
u
Genome A
Genome B
Unknown
150
100
50
0
Copy Number
37
35
50
34
57
42
41
31
49
50
42
26
57
39
22
1
2
3
4
5
6
7
unknown
Chromosome
v
Genome A
Genome B
Unknown
100
50
0
Copy Number
26
36
25
38
30
37
15
33
26
32
25
32
23
36
8
1
2
3
4
5
6
7
unknown
Chromosome
w
Genome A
Genome B
Unknown
30
20
10
0
Copy Number
4
4
8
10
8
5
5
5
9
7
9
4
12
12
4
1
2
3
4
5
6
7
unknown
Chromosome
x
Genome A
Genome B
Unknown
200
100
0
Copy Number
59
69
56
69
59
74
35
68
66
76
44
76
62
93
19
1
2
3
4
5
6
7
unknown
Chromosome

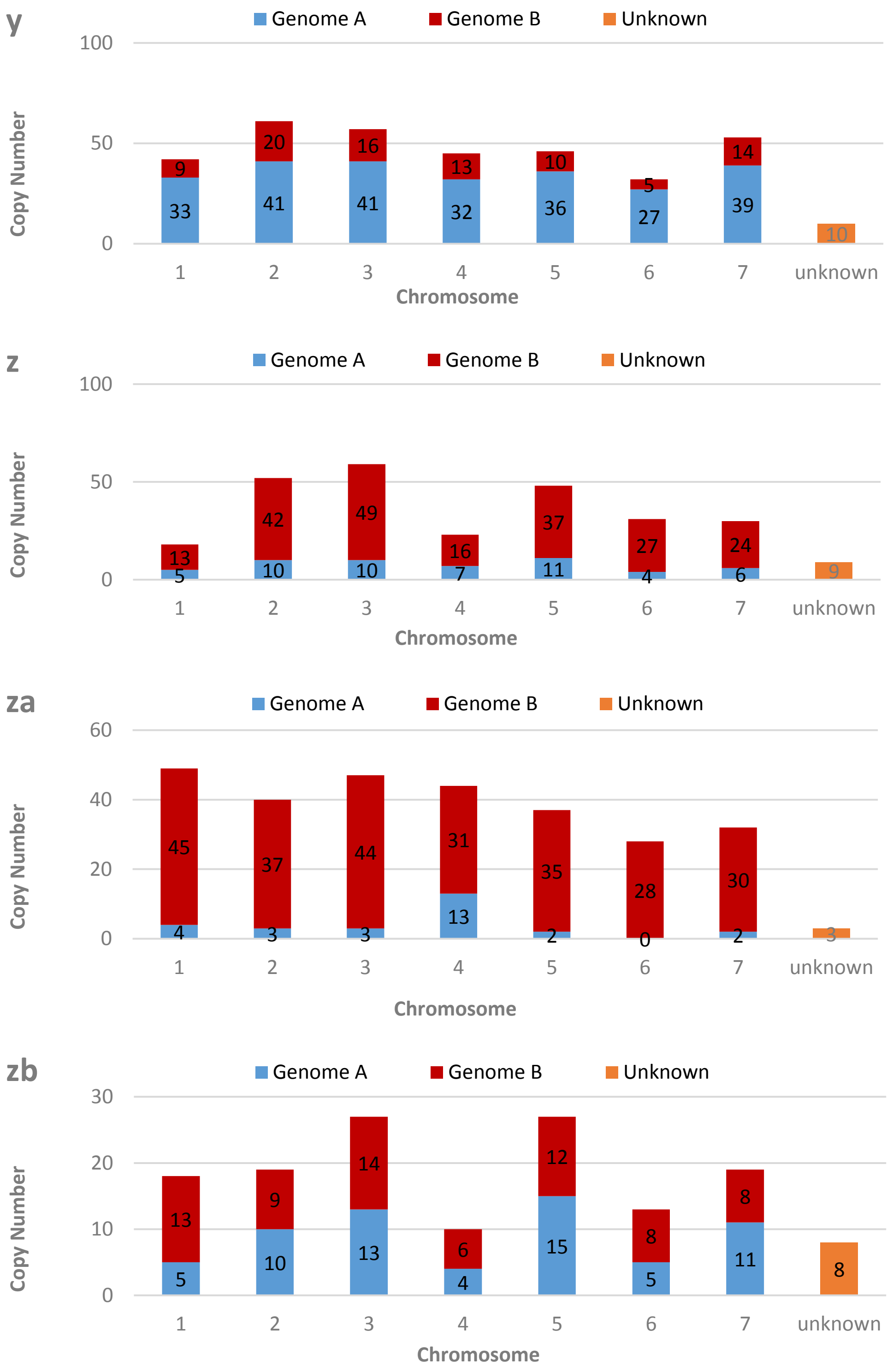
y
Genome A
Genome B
Unknown
100
50
0
Copy Number
9
33
20
41
16
41
13
32
10
36
5
27
14
39
10
1
2
3
4
5
6
7
unknown
Chromosome
z
Genome A
Genome B
Unknown
100
50
0
Copy Number
13
5
42
10
49
10
16
7
37
11
27
4
24
6
9
1
2
3
4
5
6
7
unknown
Chromosome
za
Genome A
Genome B
Unknown
60
40
20
0
Copy Number
45
4
37
3
44
3
31
13
35
2
28
0
30
2
3
1
2
3
4
5
6
7
unknown
Chromosome
zb
Genome A
Genome B
Unknown
30
20
10
0
Copy Number
13
5
9
10
14
13
6
4
12
15
8
5
8
11
8
1
2
3
4
5
6
7
unknown
Chromosome

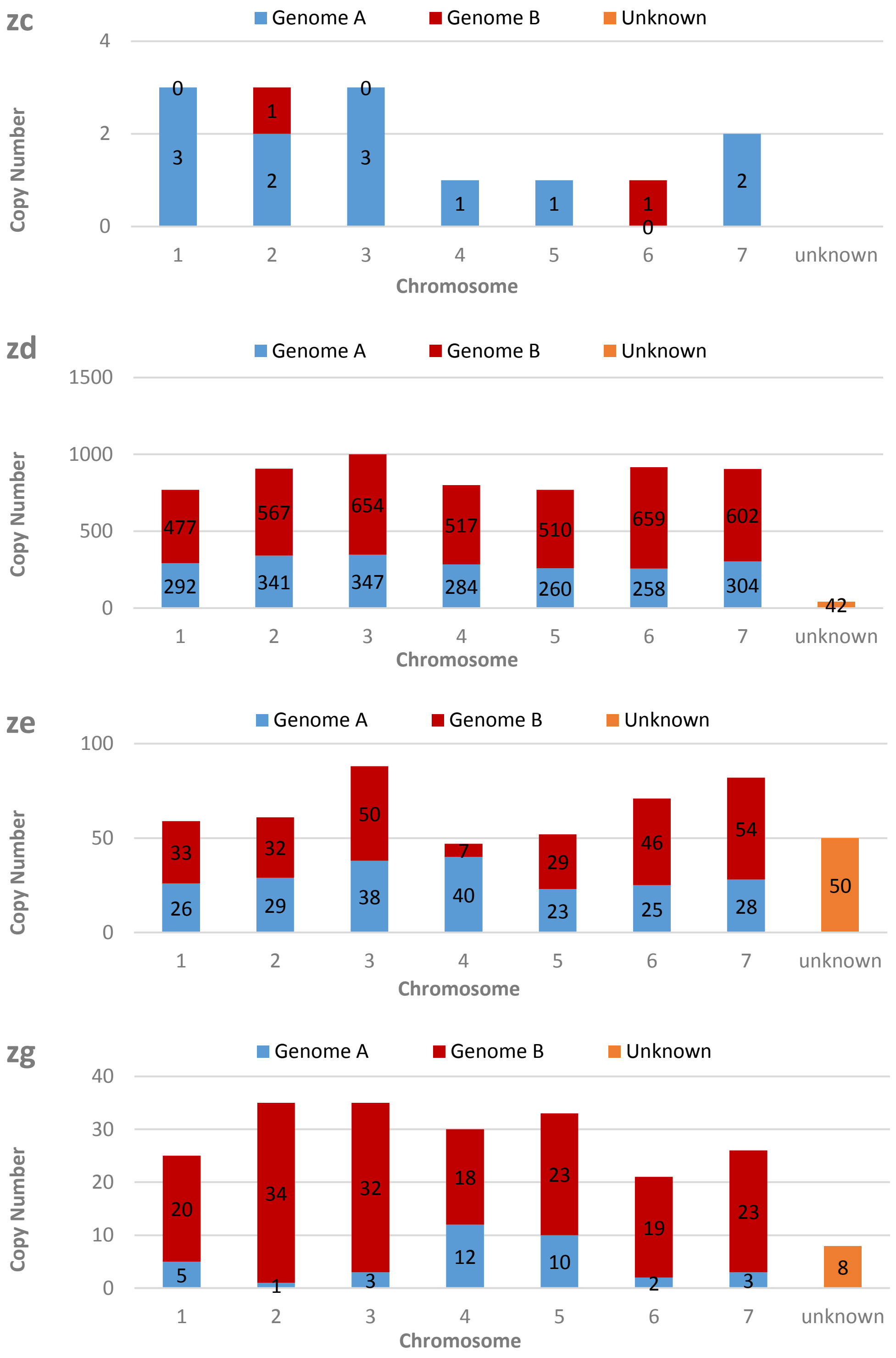
zc
Genome A
Genome B
Unknown
Copy Number
0
2
4
3
0
2
1
3
0
1
1
1
0
2
1
2
3
4
5
6
7
unknown
Chromosome
zd
Genome A
Genome B
Unknown
Copy Number
0
500
1000
1500
292
477
341
567
347
654
284
517
260
510
258
659
304
602
42
1
2
3
4
5
6
7
unknown
Chromosome
ze
Genome A
Genome B
Unknown
Copy Number
0
50
100
26
33
29
32
38
50
40
7
23
29
25
46
28
54
50
1
2
3
4
5
6
7
unknown
Chromosome
zg
Genome A
Genome B
Unknown
Copy Number
0
10
20
30
40
5
20
1
34
3
32
12
18
10
23
2
19
3
23
8
1
2
3
4
5
6
7
unknown
Chromosome

zh

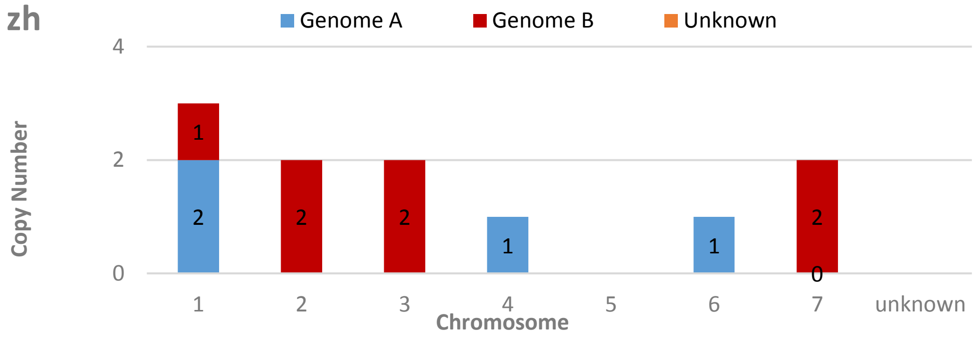

zi

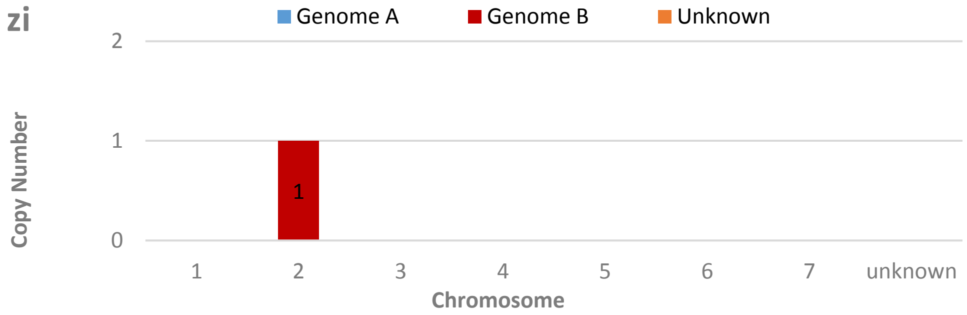

S1 Figure. Distribution of MITE families in the seven homoelogous chromosomes of *T. turgidum* ssp *diccocoides* (AABB).

a). *Thalos*. **b).** *Athos*. **c).** *Pan*. **d).** *Icarus*. **e).** *Hades*. **f).** *Eos*. **g).** *Xados*. **h).** *Minos*. **i).** *Aison*. **j).** *Stolos*. **k).** *Fortuna*. **l).** *Oleus*. **m).** *Antonio*. **n).** *Minimus*. **o).** *Tantalos*. **p).** *Phoebus*. **q).** *Polyphemus*. **r).** *Jason*. **s).** *Orpheus*. **t).** *Kerberos*. **u).** *Coeus*. **v).** *Xenon*. **w).** *Victor*. **x).** *Gerald*. **y).** *Rhea*. **z).** *Spring*. **za).** *Argus*. **zb).** *Vacuna*. **zc).** *Gabriel*. **zd).** *Belus*. **ze).** *Keres*. **zf).** *Gorgon*. **zg).** *Remus*. **zh).** *Marius* **zi).** *Murray*.
