# Supplementary material for: Genome-wide analyses of miniature inverted-repeat transposable elements reveals new insights into the evolution of the Triticum-Aegilops group
Source: PLoS One. 2018 Oct 24;13(10):e0204972. doi: 10.1371/journal.pone.0204972 (PMC6200218; doi:10.1371/journal.pone.0204972)
Supplement: S2 Fig — (PDF) [file pone.0204972.s009.pdf]

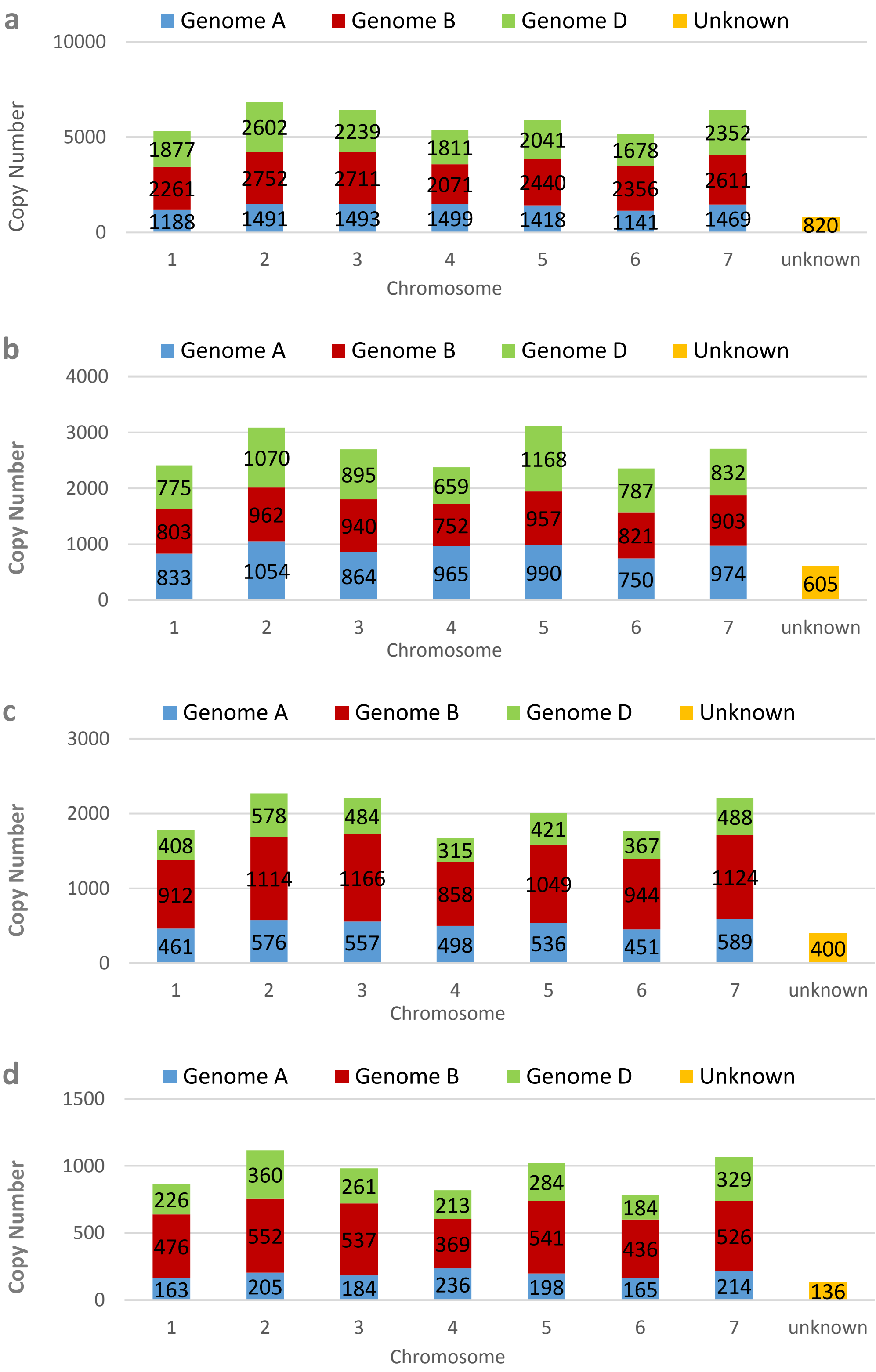
a
Genome A
Genome B
Genome D
Unknown
10000
5000
0
Copy Number
1877 2602 2239 1811 2041 1678 2352
2261 2752 2711 2071 2440 2356 2611
1188 1491 1493 1499 1418 1141 1469
820
1 2 3 4 5 6 7 unknown
Chromosome
b
Genome A
Genome B
Genome D
Unknown
4000
3000
2000
1000
0
Copy Number
775 1070 895 659 1168 787 832
803 962 940 752 957 821 903
833 1054 864 965 990 750 974
605
1 2 3 4 5 6 7 unknown
Chromosome
c
Genome A
Genome B
Genome D
Unknown
3000
2000
1000
0
Copy Number
408 578 484 315 421 367 488
912 1114 1166 858 1049 944 1124
461 576 557 498 536 451 589
400
1 2 3 4 5 6 7 unknown
Chromosome
d
Genome A
Genome B
Genome D
Unknown
1500
1000
500
0
Copy Number
226 360 261 213 284 184 329
476 552 537 369 541 436 526
163 205 184 236 198 165 214
136
1 2 3 4 5 6 7 unknown
Chromosome

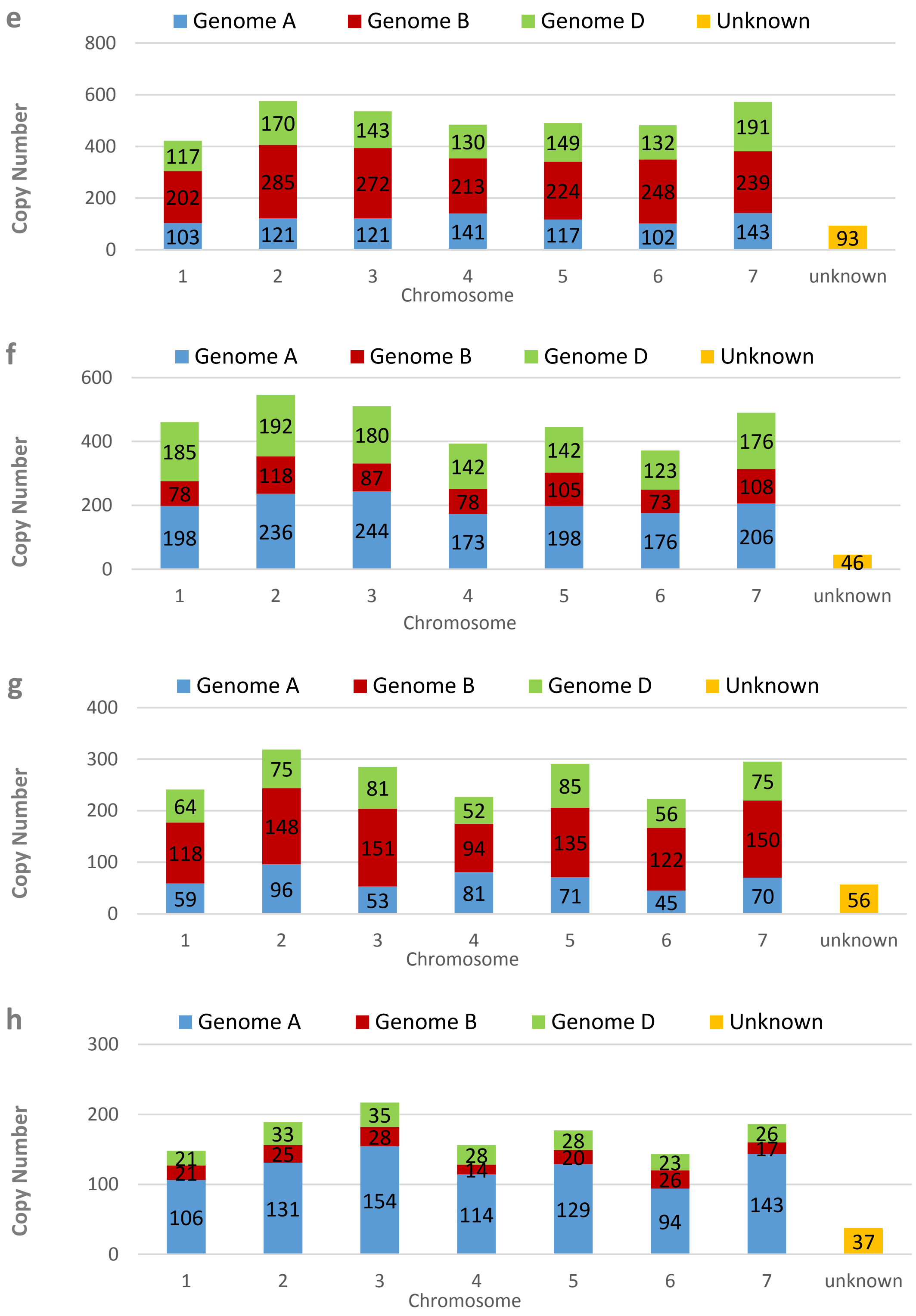
e
Genome A
Genome B
Genome D
Unknown
Copy Number
800
600
400
200
0
117 202 103
170 285 121
143 272 121
130 213 141
149 224 117
132 248 102
191 239 143
93
1 2 3 4 5 6 7 unknown
Chromosome
f
Genome A
Genome B
Genome D
Unknown
Copy Number
600
400
200
0
185 78 198
192 118 236
180 87 244
142 78 173
142 105 198
123 73 176
176 108 206
46
1 2 3 4 5 6 7 unknown
Chromosome
g
Genome A
Genome B
Genome D
Unknown
Copy Number
400
300
200
100
0
64 118 59
75 148 96
81 151 53
52 94 81
85 135 71
56 122 45
75 150 70
56
1 2 3 4 5 6 7 unknown
Chromosome
h
Genome A
Genome B
Genome D
Unknown
Copy Number
300
200
100
0
21 21 106
33 25 131
35 28 154
28 14 114
28 20 129
23 26 94
26 17 143
37
1 2 3 4 5 6 7 unknown
Chromosome

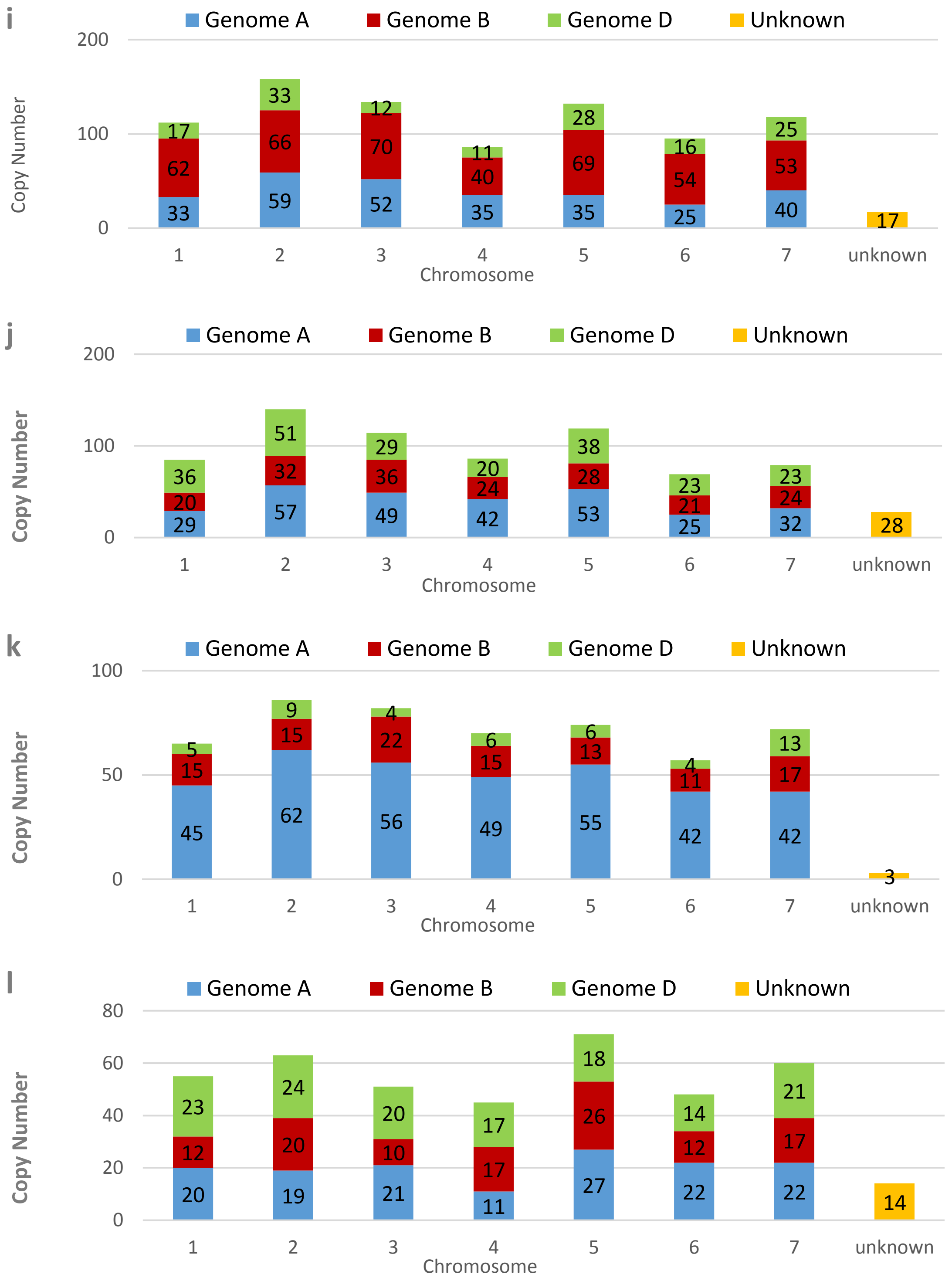
i
Genome A
Genome B
Genome D
Unknown
Copy Number
200
100
0
17
62
33
33
66
59
12
70
52
11
40
35
28
69
35
16
54
25
25
53
40
17
1
2
3
4
5
6
7
unknown
Chromosome
j
Genome A
Genome B
Genome D
Unknown
Copy Number
200
100
0
36
20
29
51
32
57
29
36
49
20
24
42
38
28
53
23
21
25
23
24
32
28
1
2
3
4
5
6
7
unknown
Chromosome
k
Genome A
Genome B
Genome D
Unknown
Copy Number
100
50
0
5
15
45
9
15
62
4
22
56
6
15
49
6
13
55
4
11
42
13
17
42
3
1
2
3
4
5
6
7
unknown
Chromosome
l
Genome A
Genome B
Genome D
Unknown
Copy Number
80
60
40
20
0
23
12
20
24
20
19
20
10
21
17
17
11
18
26
27
14
12
22
21
17
22
14
1
2
3
4
5
6
7
unknown
Chromosome

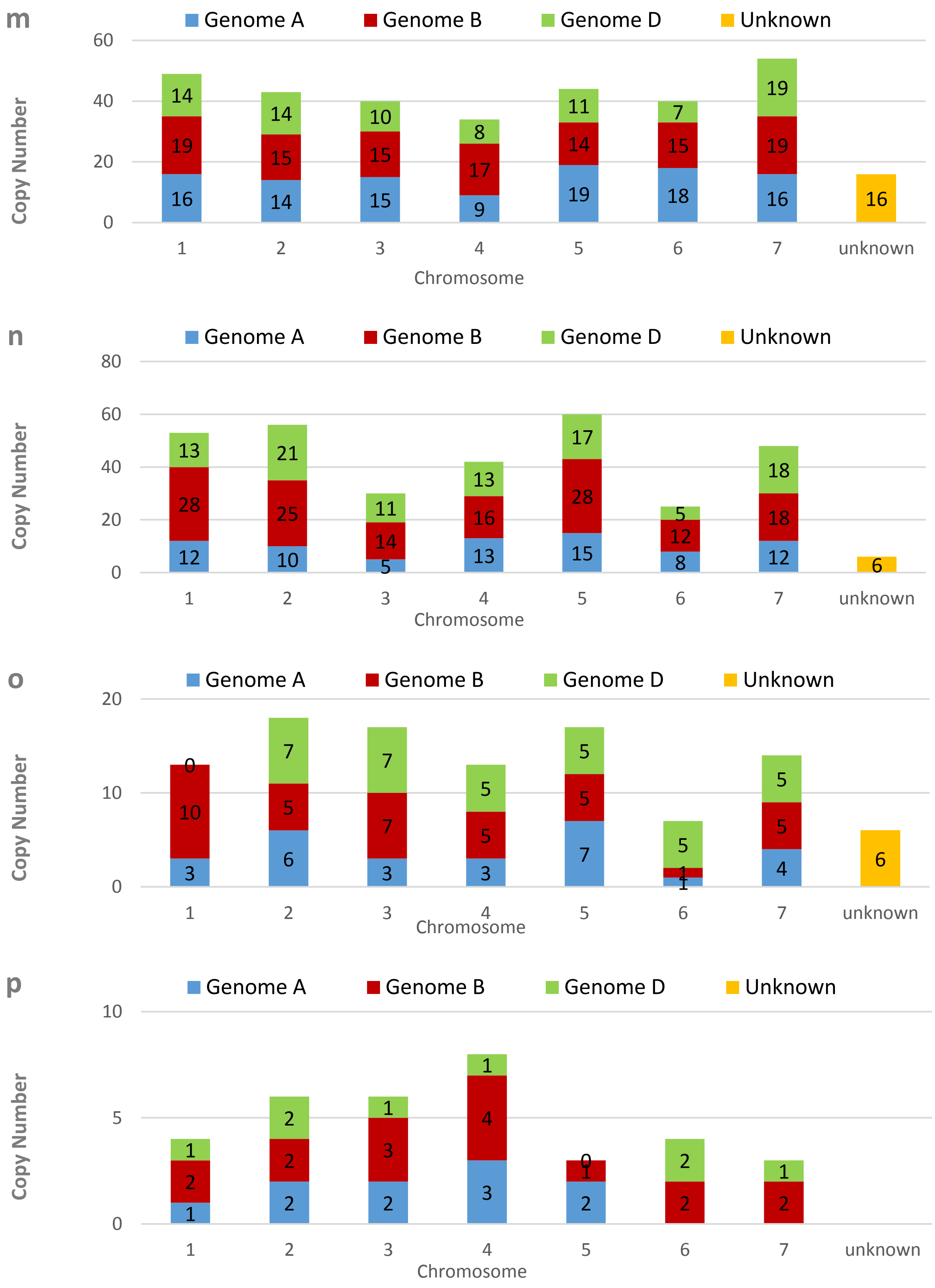
m
Genome A
Genome B
Genome D
Unknown
Copy Number
60
40
20
0
14 19 16
14 15 14
10 15 15
8 17 9
11 14 19
7 15 18
19 19 16
16
1 2 3 4 5 6 7 unknown
Chromosome
n
Genome A
Genome B
Genome D
Unknown
Copy Number
80
60
40
20
0
13 28 12
21 25 10
11 14 5
13 16 13
17 28 15
5 12 8
18 18 12
6
1 2 3 4 5 6 7 unknown
Chromosome
o
Genome A
Genome B
Genome D
Unknown
Copy Number
20
10
0
0 10 3
7 5 6
7 7 3
5 5 3
5 5 7
5 1 1
5 5 4
6
1 2 3 4 5 6 7 unknown
Chromosome
p
Genome A
Genome B
Genome D
Unknown
Copy Number
10
5
0
1 2 1
2 2 2
1 3 2
1 4 3
0 1 2
2 2
1 2
1 2 3 4 5 6 7 unknown
Chromosome

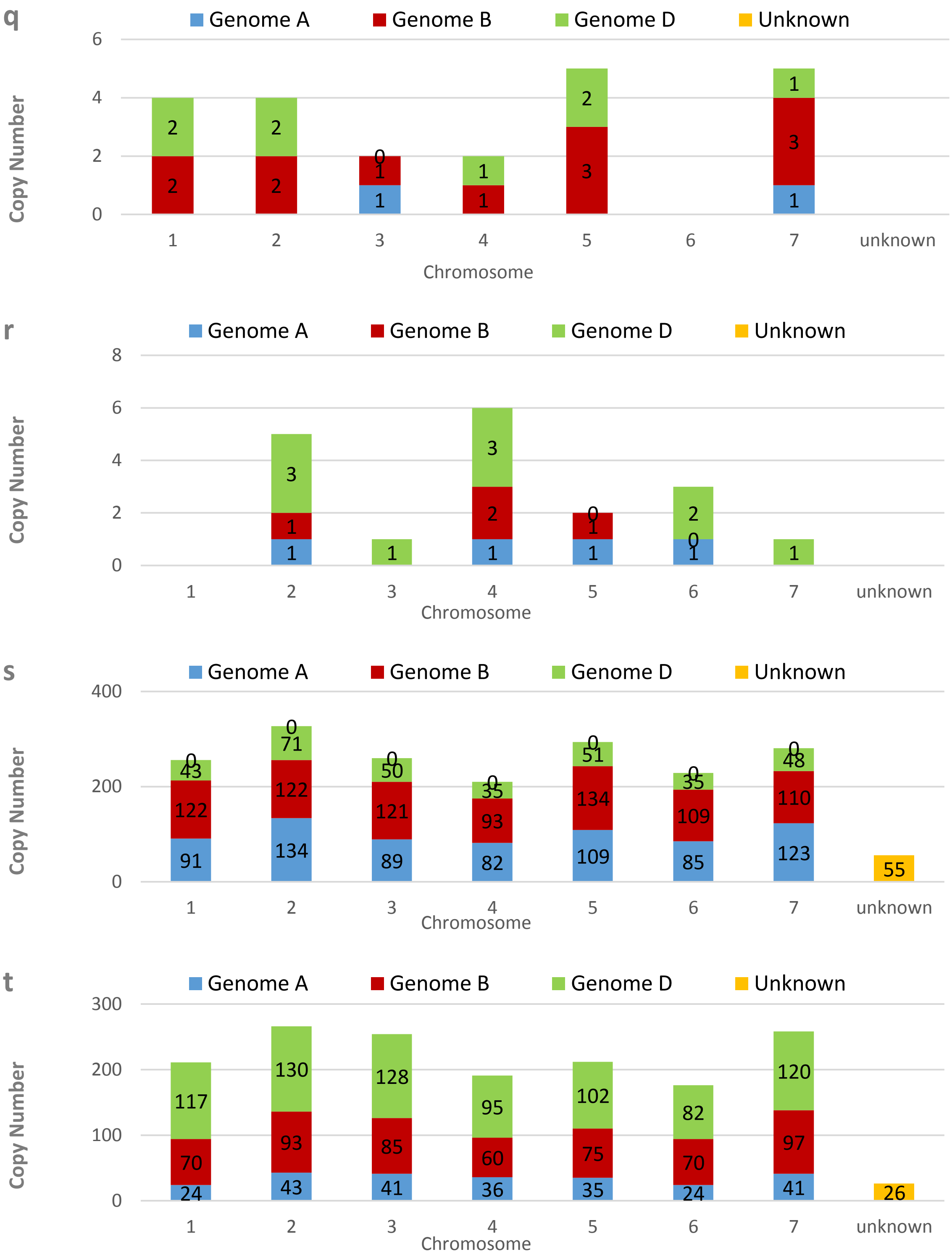
q
Genome A
Genome B
Genome D
Unknown
Copy Number
Chromosome
1
2
3
4
5
6
7
unknown
r
Genome A
Genome B
Genome D
Unknown
Copy Number
Chromosome
s
Genome A
Genome B
Genome D
Unknown
Copy Number
Chromosome
t
Genome A
Genome B
Genome D
Unknown
Copy Number
Chromosome

u

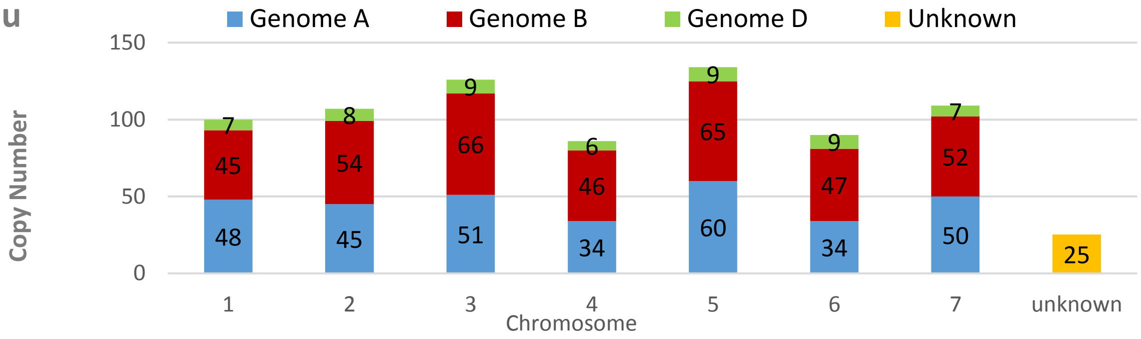

Genome A
Genome B
Genome D
Unknown
Copy Number
150
100
50
0
48 45 7
45 54 8
51 66 9
34 46 6
60 65 9
34 47 9
50 52 7
25
1
2
3
4
5
6
7
unknown
Chromosome

v

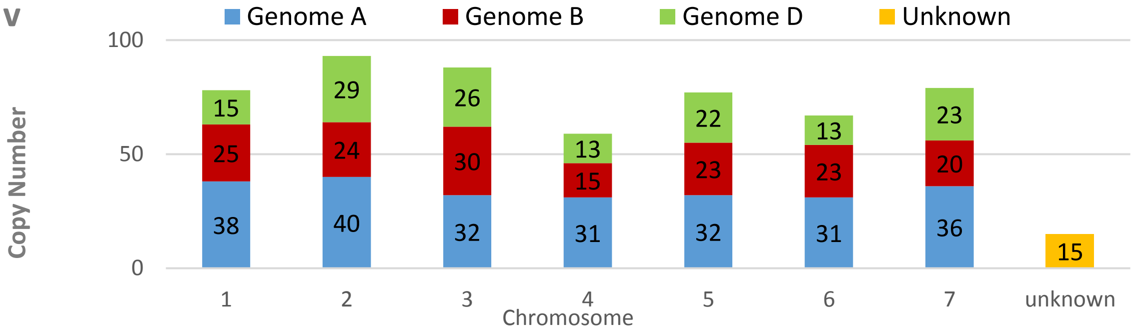

Genome A
Genome B
Genome D
Unknown
Copy Number
100
50
0
38 25 15
40 24 29
32 30 26
31 15 13
32 23 22
31 23 13
36 20 23
15
1
2
3
4
5
6
7
unknown
Chromosome

w

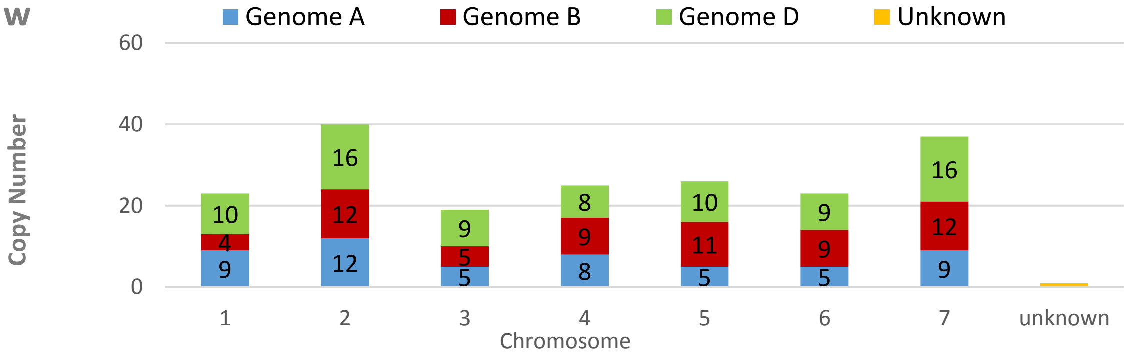

Genome A
Genome B
Genome D
Unknown
Copy Number
60
40
20
0
9 4 10
12 12 16
5 5 9
8 9 8
5 11 10
5 9 9
9 12 16
1
2
3
4
5
6
7
unknown
Chromosome

x

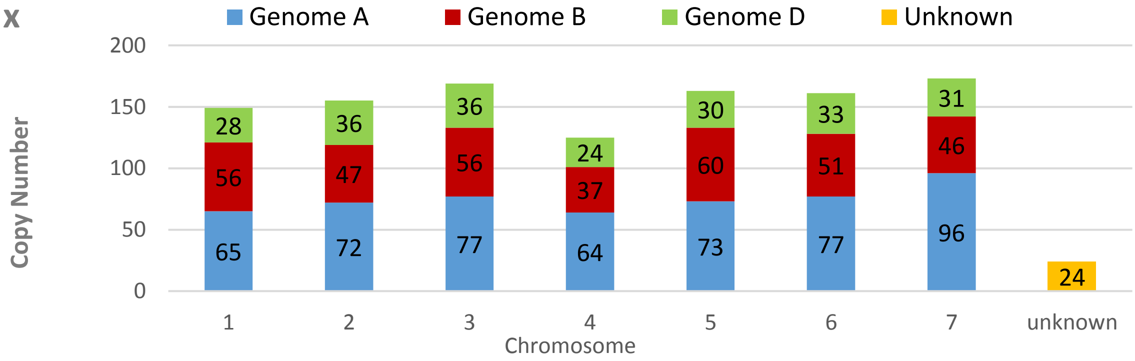

Genome A
Genome B
Genome D
Unknown
Copy Number
200
150
100
50
0
65 56 28
72 47 36
77 56 36
64 37 24
73 60 30
77 51 33
96 46 31
24
1
2
3
4
5
6
7
unknown
Chromosome

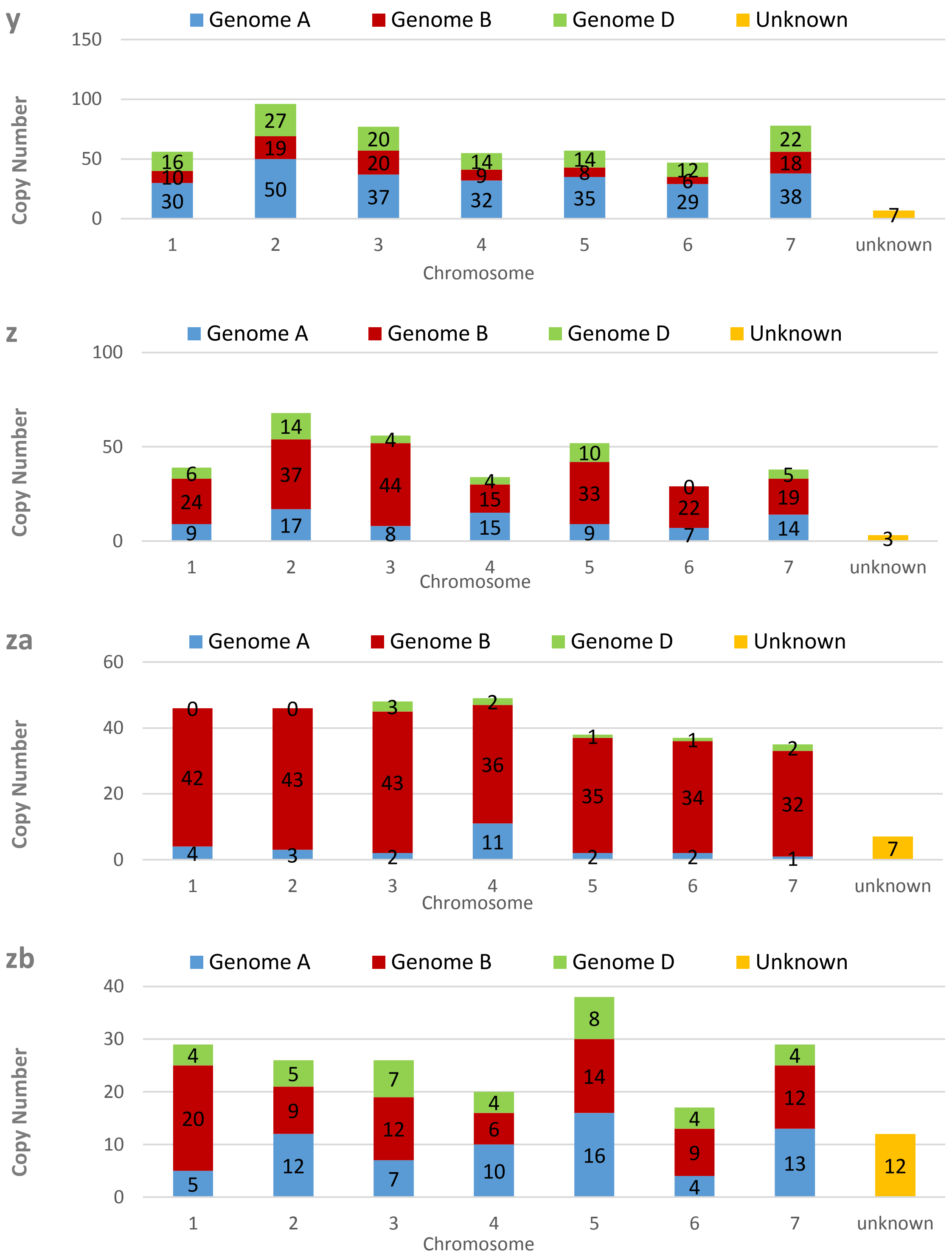
y
Genome A
Genome B
Genome D
Unknown
Copy Number
150
100
50
0
1
2
3
4
5
6
7
unknown
Chromosome
30 10 16
50 19 27
37 20 20
32 9 14
35 8 14
29 6 12
38 18 22
7
z
Genome A
Genome B
Genome D
Unknown
Copy Number
100
50
0
1
2
3
4
5
6
7
unknown
Chromosome
9 24 6
17 37 14
8 44 4
15 15 4
9 33 10
7 22 0
14 19 5
3
za
Genome A
Genome B
Genome D
Unknown
Copy Number
60
40
20
0
1
2
3
4
5
6
7
unknown
Chromosome
4 42 0
3 43 0
2 43 3
11 36 2
2 35 1
2 34 1
1 32 2
7
zb
Genome A
Genome B
Genome D
Unknown
Copy Number
40
30
20
10
0
1
2
3
4
5
6
7
unknown
Chromosome
5 20 4
12 9 5
7 12 7
10 6 4
16 14 8
4 9 4
13 12 4
12

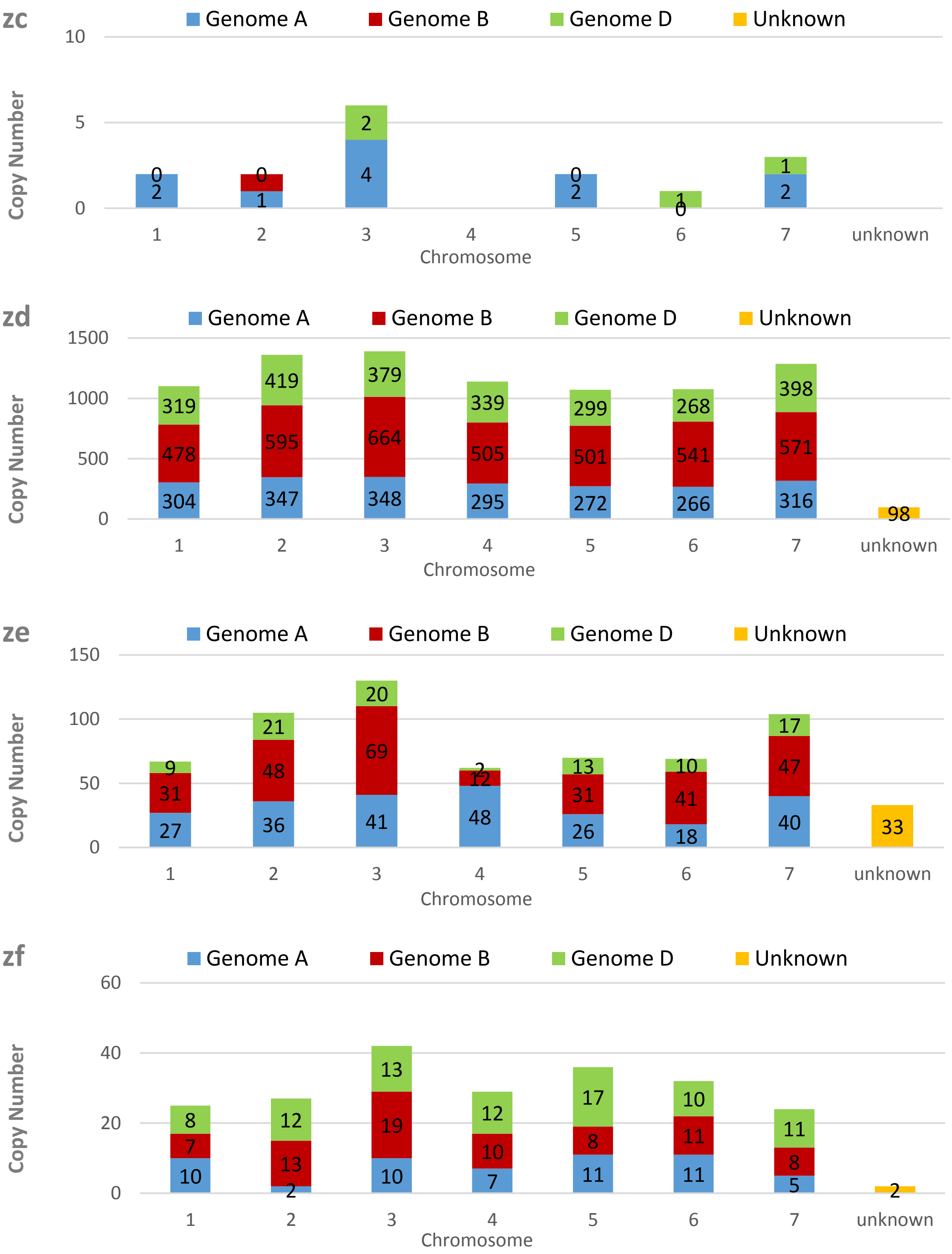
zc
Genome A
Genome B
Genome D
Unknown
Copy Number
10
5
0
0
2
0
1
2
4
0
2
1
0
1
2
1
2
3
4
5
6
7
unknown
Chromosome
zd
Genome A
Genome B
Genome D
Unknown
Copy Number
1500
1000
500
0
319
478
304
419
595
347
379
664
348
339
505
295
299
501
272
268
541
266
398
571
316
98
1
2
3
4
5
6
7
unknown
Chromosome
ze
Genome A
Genome B
Genome D
Unknown
Copy Number
150
100
50
0
9
31
27
21
48
36
20
69
41
2
12
48
13
31
26
10
41
18
17
47
40
33
1
2
3
4
5
6
7
unknown
Chromosome
zf
Genome A
Genome B
Genome D
Unknown
Copy Number
60
40
20
0
8
7
10
12
13
2
13
19
10
12
10
7
17
8
11
10
11
11
11
8
5
2
1
2
3
4
5
6
7
unknown
Chromosome

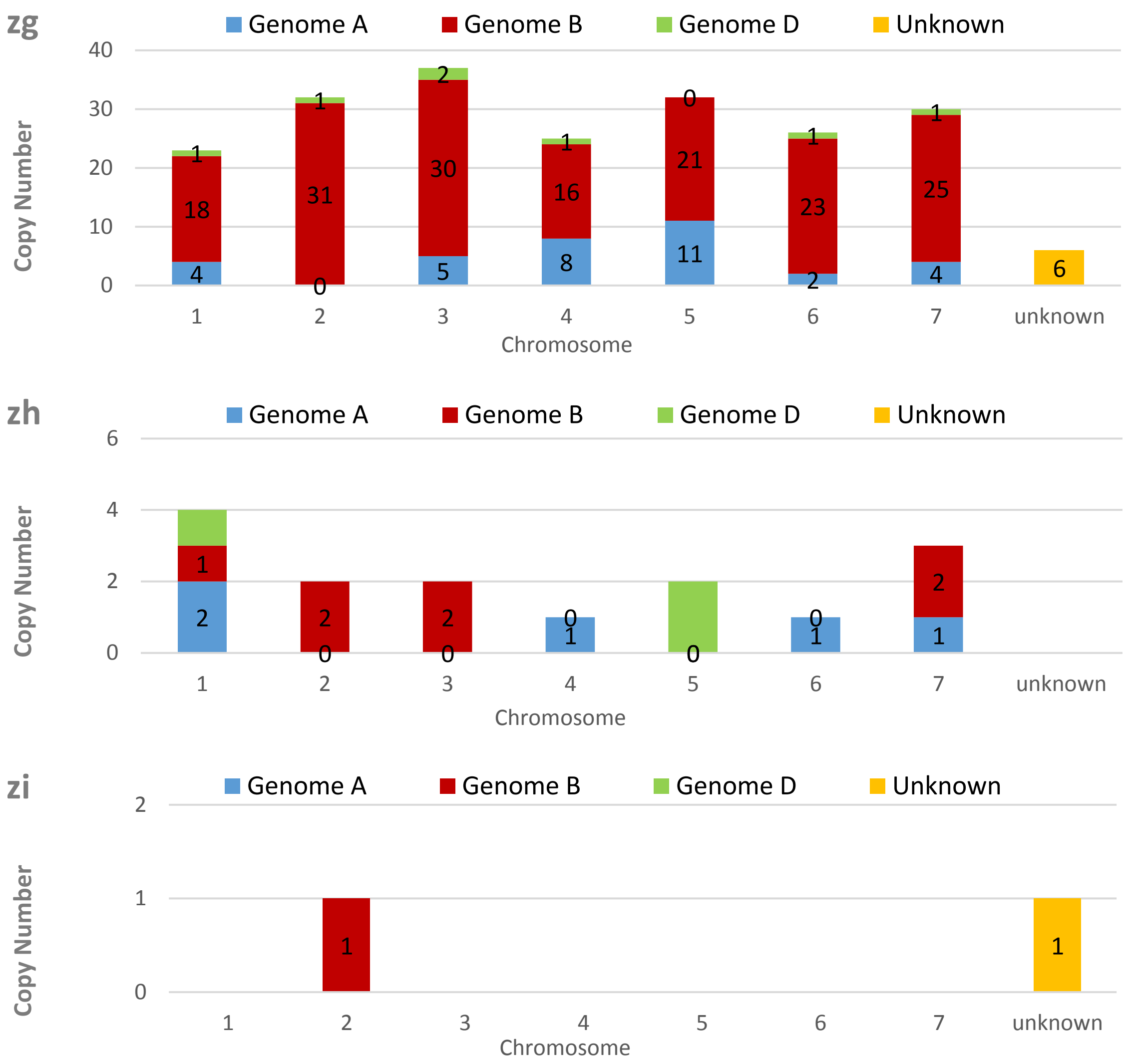

S2 Figure. Distribution of MITE families in the seven homoelogous chromosomes of *T aestivum* (AABBDD).

a). *Thalos*. **b).** *Athos*. **c).** *Pan*. **d).** *Icarus*. **e).** *Hades*. **f).** *Eos*. **g).** *Xados*. **h).** *Minos*. **i).** *Aison*. **j).** *Stolos*. **k).** *Fortuna*. **l).** *Oleus*. **m).** *Antonio*. **n).** *Minimus*. **o).** *Tantalos*. **p).** *Phoebus*. **q).** *Polyphemus*. **r).** *Jason*. **s).** *Orpheus*. **t).** *Kerberos*. **u).** *Coeus*. **v).** *Xenon*. **w).** *Victor*. **x).** *Gerald*. **y).** *Rhea*. **z).** *Spring*. **za).** *Argus*. **zb).** *Vacuna*. **zc).** *Gabriel*. **zd).** *Belus*. **ze).** *Keres*. **zf).** *Gorgon*. **zg).** *Remus*. **zh).** *Marius* **zi).** *Murray*.
