# Supplementary material for: Genome-wide analyses of miniature inverted-repeat transposable elements reveals new insights into the evolution of the Triticum-Aegilops group
Source: PLoS One. 2018 Oct 24;13(10):e0204972. doi: 10.1371/journal.pone.0204972 (PMC6200218; doi:10.1371/journal.pone.0204972)
Supplement: S3 Fig — (PDF) [file pone.0204972.s010.pdf]

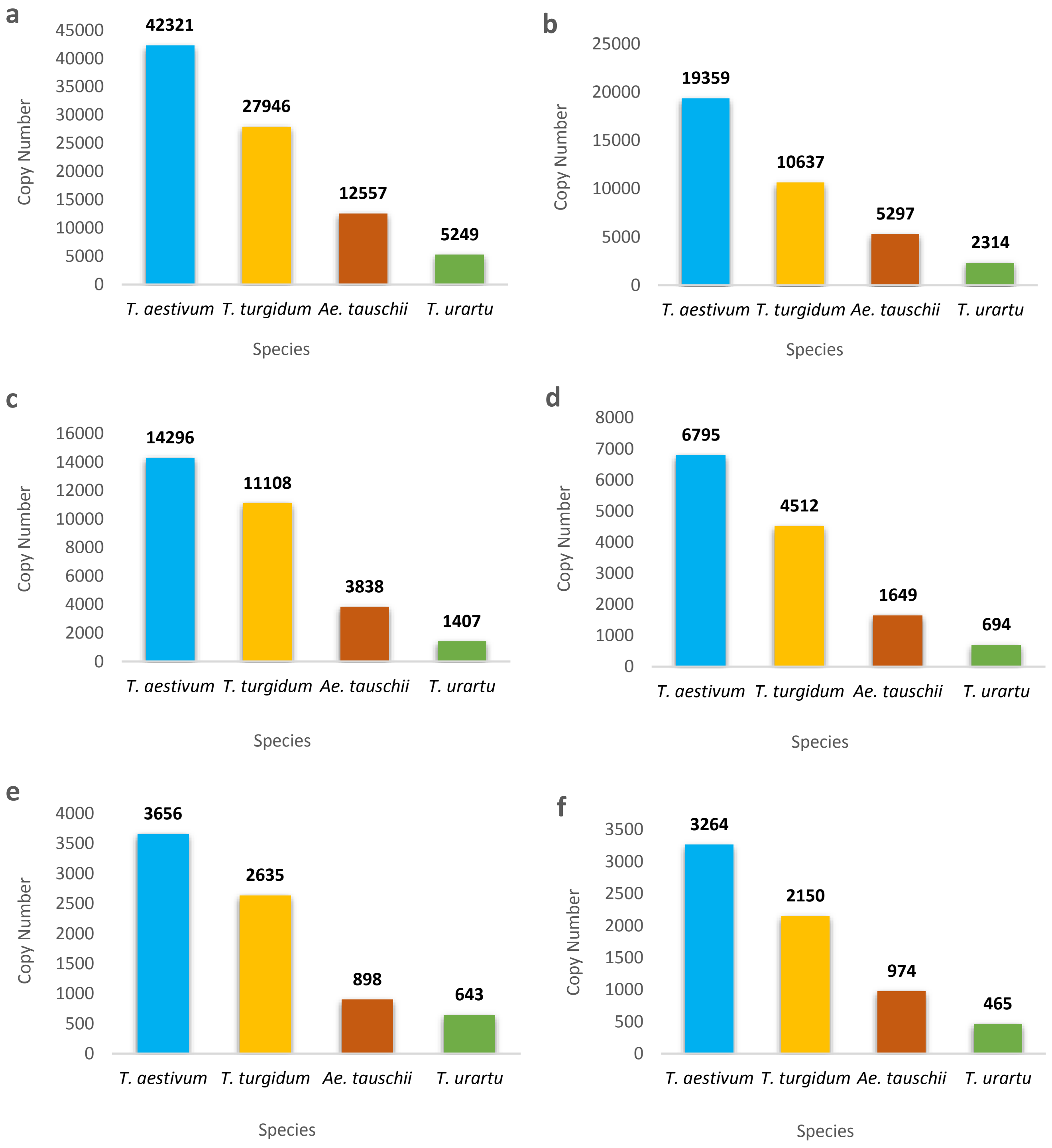
a
Copy Number
45000
40000
35000
30000
25000
20000
15000
10000
5000
0
42321
27946
12557
5249
T. aestivum
T. turgidum
Ae. tauschii
T. urartu
Species
b
Copy Number
25000
20000
15000
10000
5000
0
19359
10637
5297
2314
T. aestivum
T. turgidum
Ae. tauschii
T. urartu
Species
c
Copy Number
16000
14000
12000
10000
8000
6000
4000
2000
0
14296
11108
3838
1407
T. aestivum
T. turgidum
Ae. tauschii
T. urartu
Species
d
Copy Number
8000
7000
6000
5000
4000
3000
2000
1000
0
6795
4512
1649
694
T. aestivum
T. turgidum
Ae. tauschii
T. urartu
Species
e
Copy Number
4000
3500
3000
2500
2000
1500
1000
500
0
3656
2635
898
643
T. aestivum
T. turgidum
Ae. tauschii
T. urartu
Species
f
Copy Number
3500
3000
2500
2000
1500
1000
500
0
3264
2150
974
465
T. aestivum
T. turgidum
Ae. tauschii
T. urartu
Species

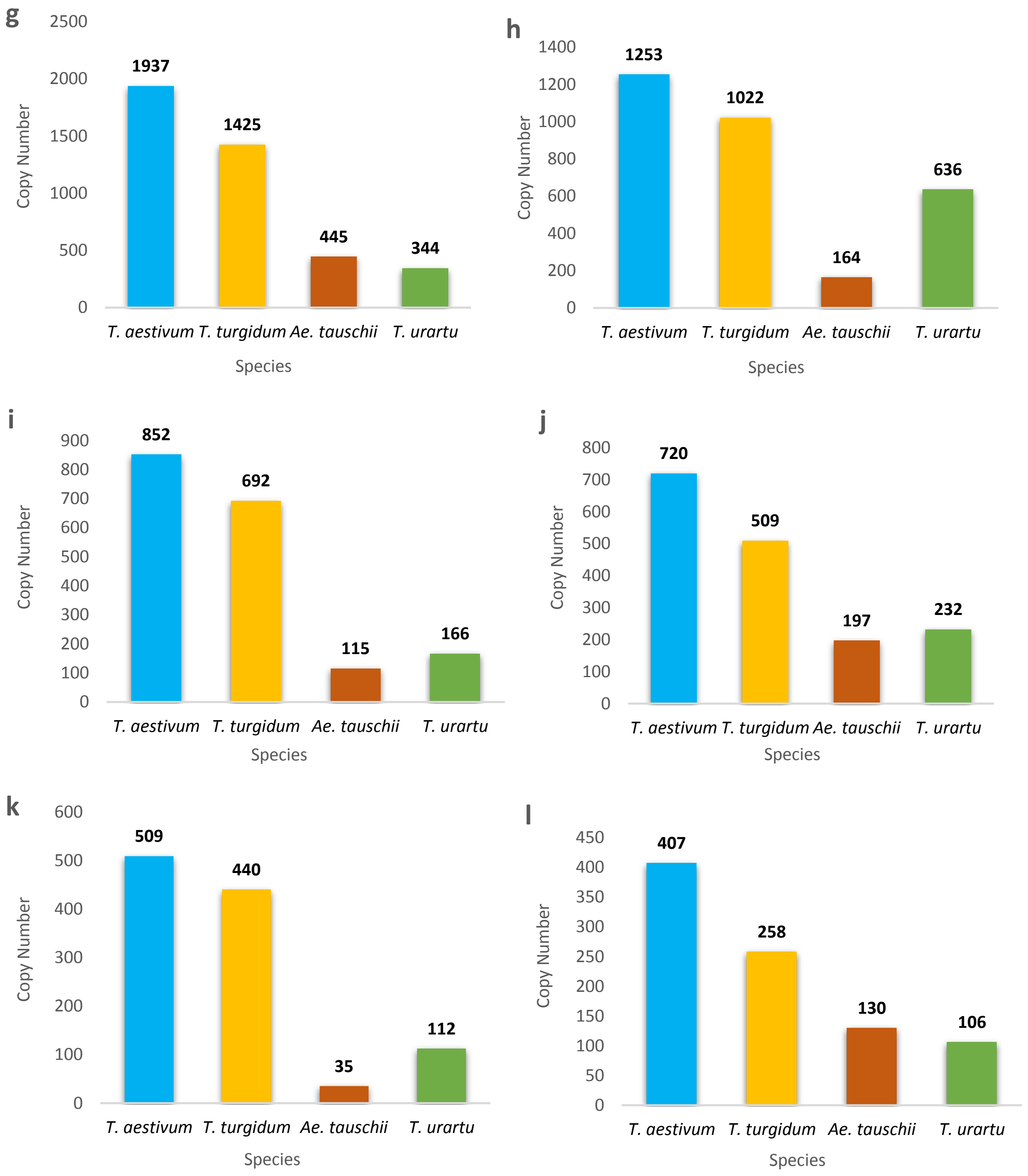
g
Copy Number
2500
2000
1500
1000
500
0
1937
1425
445
344
T. aestivum
T. turgidum
Ae. tauschii
T. urartu
Species
h
Copy Number
1400
1200
1000
800
600
400
200
0
1253
1022
164
636
T. aestivum
T. turgidum
Ae. tauschii
T. urartu
Species
i
Copy Number
900
800
700
600
500
400
300
200
100
0
852
692
115
166
T. aestivum
T. turgidum
Ae. tauschii
T. urartu
Species
j
Copy Number
800
700
600
500
400
300
200
100
0
720
509
197
232
T. aestivum
T. turgidum
Ae. tauschii
T. urartu
Species
k
Copy Number
600
500
400
300
200
100
0
509
440
35
112
T. aestivum
T. turgidum
Ae. tauschii
T. urartu
Species
l
Copy Number
450
400
350
300
250
200
150
100
50
0
407
258
130
106
T. aestivum
T. turgidum
Ae. tauschii
T. urartu
Species

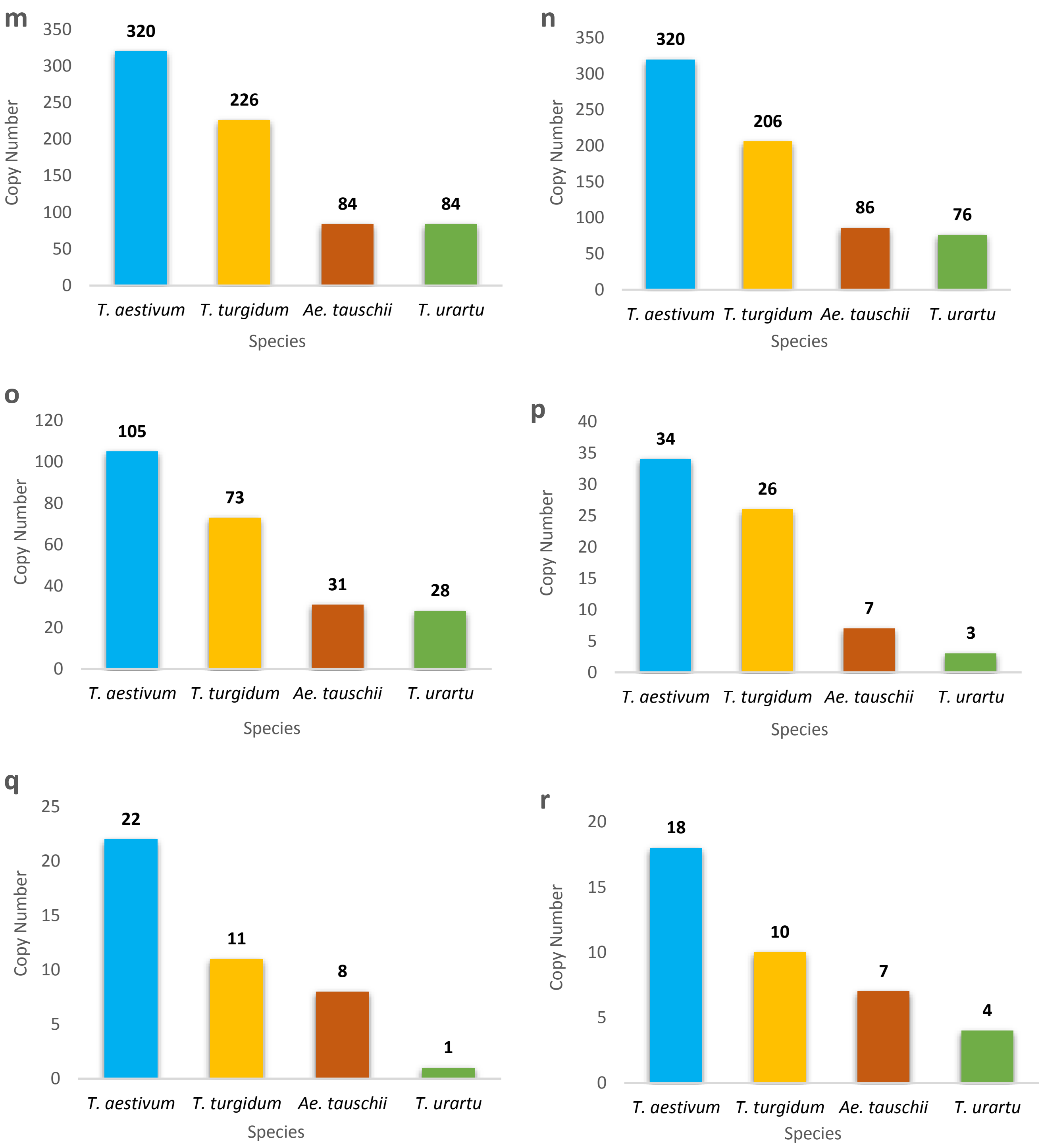
m
Copy Number
Species
T. aestivum
T. turgidum
Ae. tauschii
T. urartu
320
226
84
84
n
Copy Number
Species
320
206
86
76
o
Copy Number
Species
105
73
31
28
p
Copy Number
Species
34
26
7
3
q
Copy Number
Species
22
11
8
1
r
Copy Number
Species
18
10
7
4

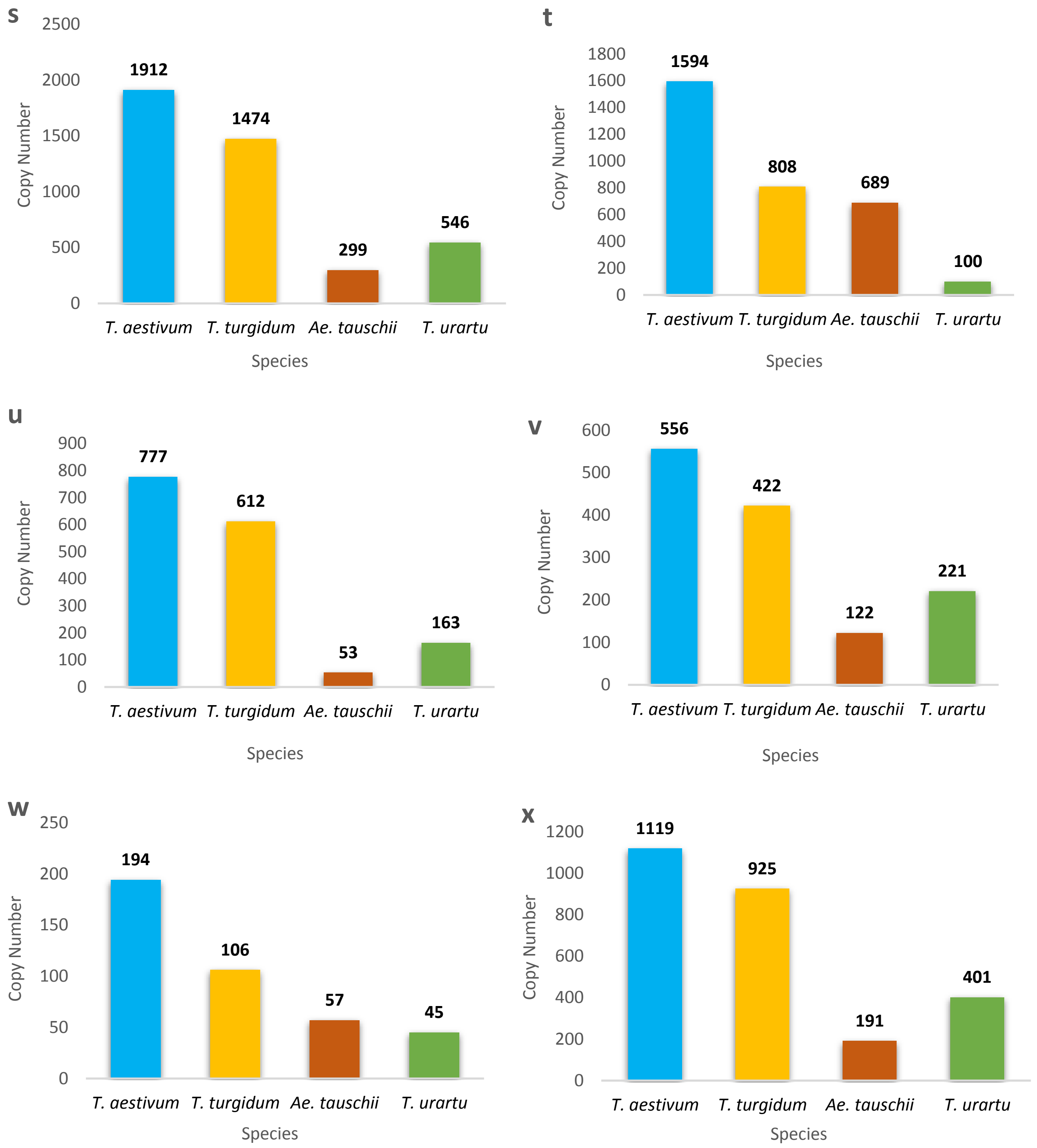
s
Copy Number
2500
2000
1500
1000
500
0
1912
1474
299
546
T. aestivum
T. turgidum
Ae. tauschii
T. urartu
Species
t
Copy Number
1800
1600
1400
1200
1000
800
600
400
200
0
1594
808
689
100
T. aestivum
T. turgidum
Ae. tauschii
T. urartu
Species
u
Copy Number
900
800
700
600
500
400
300
200
100
0
777
612
53
163
T. aestivum
T. turgidum
Ae. tauschii
T. urartu
Species
v
Copy Number
600
500
400
300
200
100
0
556
422
122
221
T. aestivum
T. turgidum
Ae. tauschii
T. urartu
Species
w
Copy Number
250
200
150
100
50
0
194
106
57
45
T. aestivum
T. turgidum
Ae. tauschii
T. urartu
Species
x
Copy Number
1200
1000
800
600
400
200
0
1119
925
191
401
T. aestivum
T. turgidum
Ae. tauschii
T. urartu
Species

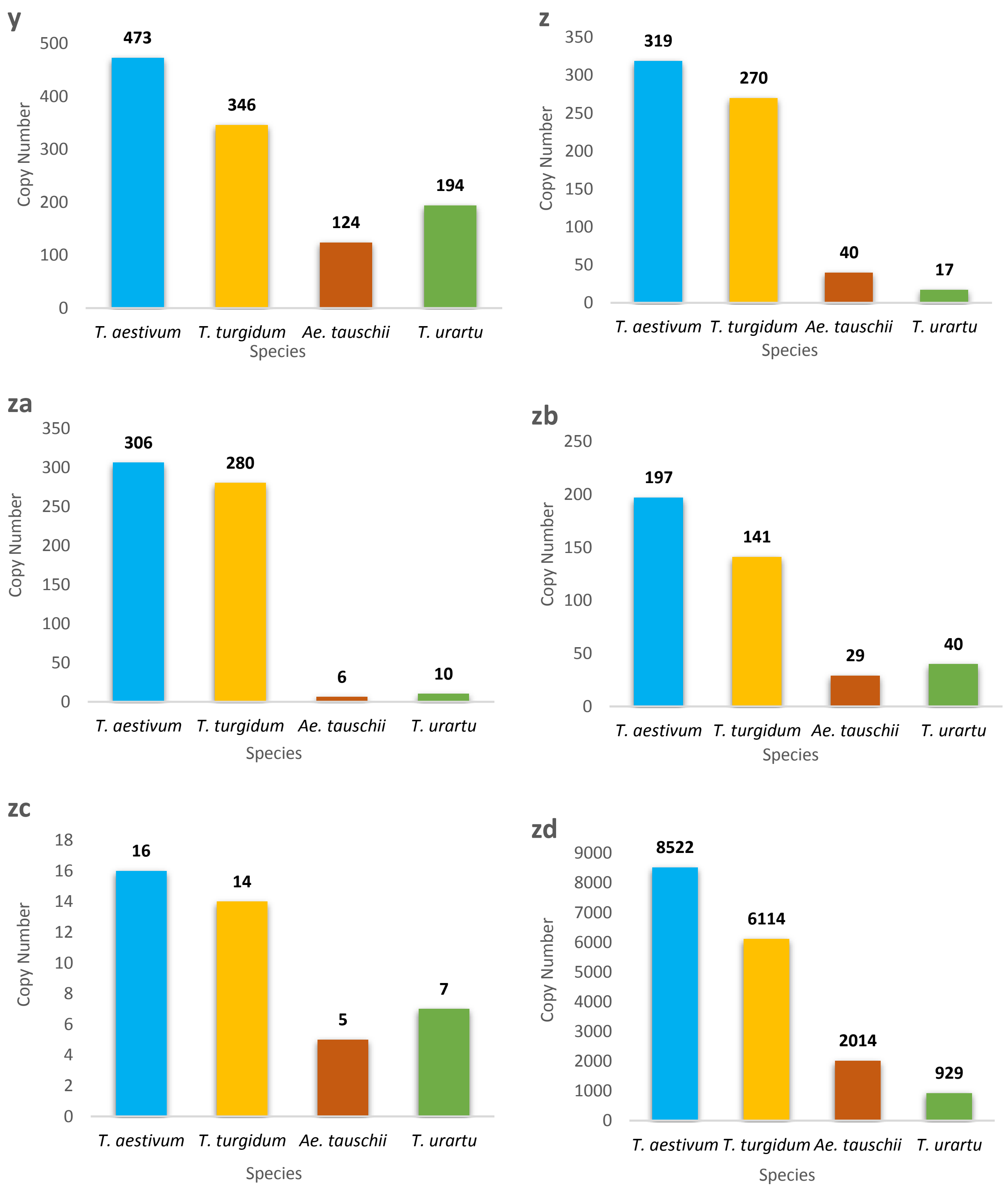

y
Copy Number
500
400
300
200
100
0
473
346
124
194
T. aestivum
T. turgidum
Ae. tauschii
T. urartu
Species
z
Copy Number
350
300
250
200
150
100
50
0
319
270
40
17
T. aestivum
T. turgidum
Ae. tauschii
T. urartu
Species
za
Copy Number
350
300
250
200
150
100
50
0
306
280
6
10
T. aestivum
T. turgidum
Ae. tauschii
T. urartu
Species
zb
Copy Number
250
200
150
100
50
0
197
141
29
40
T. aestivum
T. turgidum
Ae. tauschii
T. urartu
Species
zc
Copy Number
18
16
14
12
10
8
6
4
2
0
16
14
5
7
T. aestivum
T. turgidum
Ae. tauschii
T. urartu
Species
zd
Copy Number
9000
8000
7000
6000
5000
4000
3000
2000
1000
0
8522
6114
2014
929
T. aestivum
T. turgidum
Ae. tauschii
T. urartu
Species

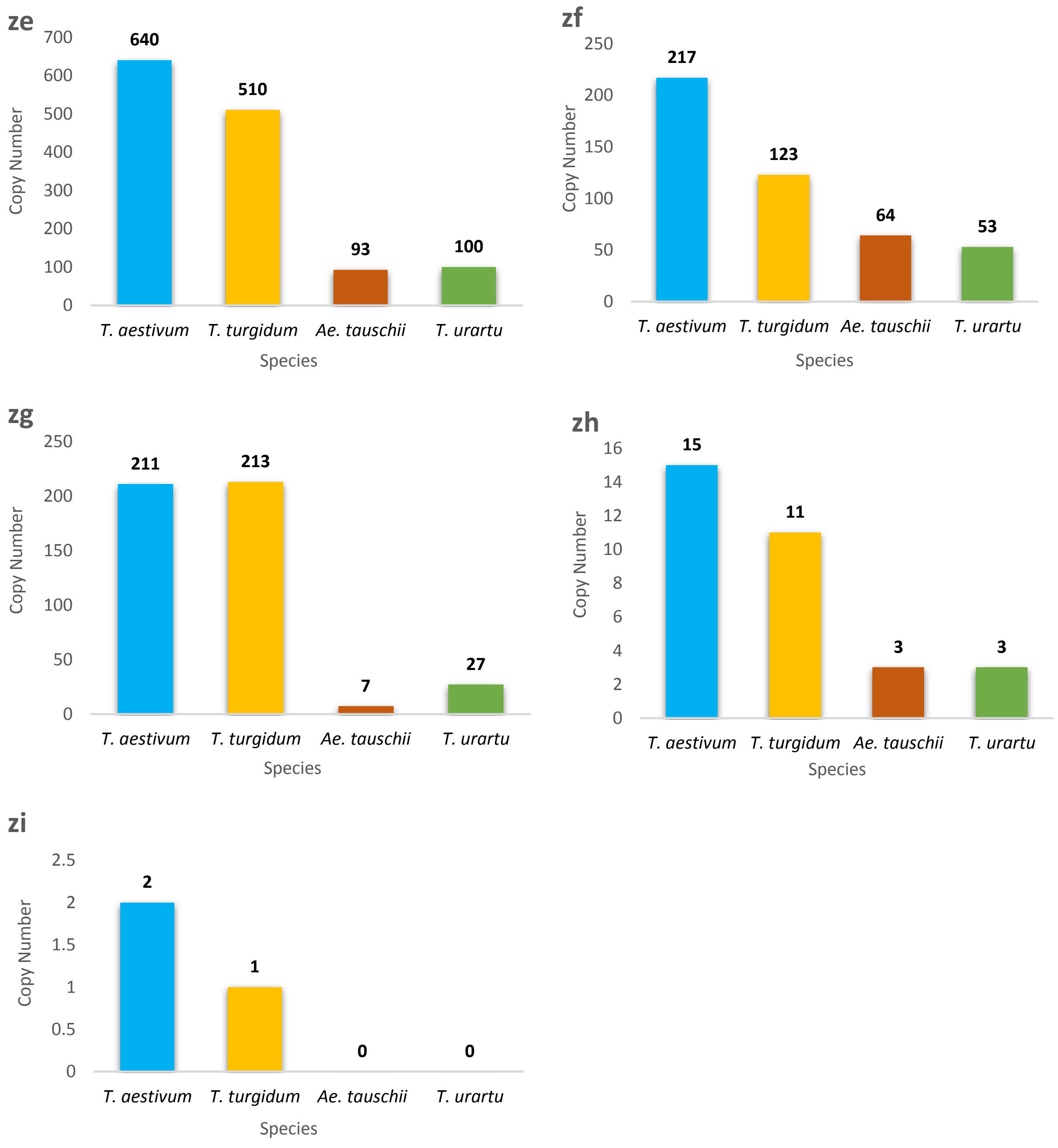

S3 Figure. Copy number of all MITE families in *Triticum* and *Aegilops* genomes.

a). *Thalos*. **b).** *Athos*. **c).** *Pan*. **d).** *Icarus*. **e).** *Hades*. **f).** *Eos*. **g).** *Xados*. **h).** *Minos*. **i).** *Aison*. **j).** *Stolos*. **k).** *Fortuna*. **l).** *Oleus*. **m).** *Antonio*. **n).** *Minimus*. **o).** *Tantalos*. **p).** *Phoebus*. **q).** *Polyphemus*. **r).** *Jason*. **s).** *Orpheus*. **t).** *Kerberos*. **u).** *Coeus*. **v).** *Xenon*. **w).** *Victor*. **x).** *Gerald*. **y).** *Rhea*. **z).** *Spring*. **za).** *Argus*. **zb).** *Vacuna*. **zc).** *Gabriel*. **zd).** *Belus*. **ze).** *Keres*. **zf).** *Gorgon*. **zg).** *Remus*. **zh).** *Marius* **zi).** *Murray*.
